# New Deoxycholic Acid Derived Tyrosyl-DNA Phosphodiesterase 1 Inhibitors Also Inhibit Tyrosyl-DNA Phosphodiesterase 2

**DOI:** 10.3390/molecules27010072

**Published:** 2021-12-23

**Authors:** Oksana V. Salomatina, Nadezhda S. Dyrkheeva, Irina I. Popadyuk, Alexandra L. Zakharenko, Ekaterina S. Ilina, Nina I. Komarova, Jóhannes Reynisson, Nariman F. Salakhutdinov, Olga I. Lavrik, Konstantin P. Volcho

**Affiliations:** 1 N.N. Vorozhtsov Novosibirsk Institute of Organic Chemistry, SB RAS, 9, Lavrent’ev Ave., 630090 Novosibirsk, Russia; ana@nioch.nsc.ru (O.V.S.); popadyuk@nioch.nsc.ru (I.I.P.); komar@nioch.nsc.ru (N.I.K.); anvar@nioch.nsc.ru (N.F.S.); 2 Institute of Chemical Biology and Fundamental Medicine, SB RAS, 8, Lavrent’ev Ave., 630090 Novosibirsk, Russia; elpida80@mail.ru (N.S.D.); sashaz@niboch.nsc.ru (A.L.Z.); katya.plekhanova@gmail.com (E.S.I.); lavrik@niboch.nsc.ru (O.I.L.); 3 School of Pharmacy and Bioengineering, Keele University, Staffordshire ST5 5BG, UK; j.reynisson@keele.ac.uk

**Keywords:** deoxycholic acid, amide, oxadiazoles, TDP1 inhibitor, TDP2 inhibitor, cancer, tumor, molecular modeling

## Abstract

A series of deoxycholic acid (DCA) amides containing benzyl ether groups on the steroid core were tested against the tyrosyl-DNA phosphodiesterase 1 (TDP1) and 2 (TDP2) enzymes. In addition, 1,2,4- and 1,3,4-oxadiazole derivatives were synthesized to study the linker influence between a *para*-bromophenyl moiety and the steroid scaffold. The DCA derivatives demonstrated promising inhibitory activity against TDP1 with IC_50_ in the submicromolar range. Furthermore, the amides and the 1,3,4-oxadiazole derivatives inhibited the TDP2 enzyme but at substantially higher concentration. Tryptamide **5** and *para*-bromoanilide **8** derivatives containing benzyloxy substituent at the C-3 position and non-substituted hydroxy group at C-12 on the DCA scaffold inhibited both TDP1 and TDP2 as well as enhanced the cytotoxicity of topotecan in non-toxic concentration in vitro. According to molecular modeling, ligand **5** is anchored into the catalytic pocket of TDP1 by one hydrogen bond to the backbone of Gly458 as well as by π–π stacking between the indolyl rings of the ligand and Tyr590, resulting in excellent activity. It can therefore be concluded that these derivatives contribute to the development of specific TDP1 and TDP2 inhibitors for adjuvant therapy against cancer in combination with topoisomerase poisons.

## 1. Introduction

Traditional chemo- and radiotherapy of oncological diseases are aimed at damaging DNA in malignant cells. An effective DNA repair system can lead to resistance to therapeutic agents of cancer cells, making DNA repair enzymes promising targets for adjunct anti-cancer drugs [1]. Topoisomerase inhibitors (TOP1: topotecan and irinotecan, TOP2: etoposide and doxorubicin) stabilize covalent topoisomerase/DNA complexes, resulting in the accumulation of DNA breaks and consequently cell death. DNA reparation of such covalent complexes is aided by tyrosyl-DNA phosphodiesterases 1 and 2 (TDP1 and TDP2), which play a significant role in the development of drug resistance [2]. Thus, TDP1 and TDP2 are considered as potential targets for adjunct therapy in combination with topoisomerase inhibitors. TDP1 catalyzes the hydrolysis of adducts covalently bound to DNA via the 3′-phosphate moiety including the products of proteolysis of TOP1–inhibitor complexes (TOP1 peptides), while TDP2 hydrolyzes 5’-phosphothyrosine and TOP2 adducts to DNA including TOP2 peptides [2]. It should be noted that the TDP1 and TDP2 enzymes have overlapping functions, albeit with less efficiency [3,4,5]. Consequently, the discovery of dual inhibitors of TDP1 and TDP2 is a promising strategy to be pursued.

**Figure 1:**
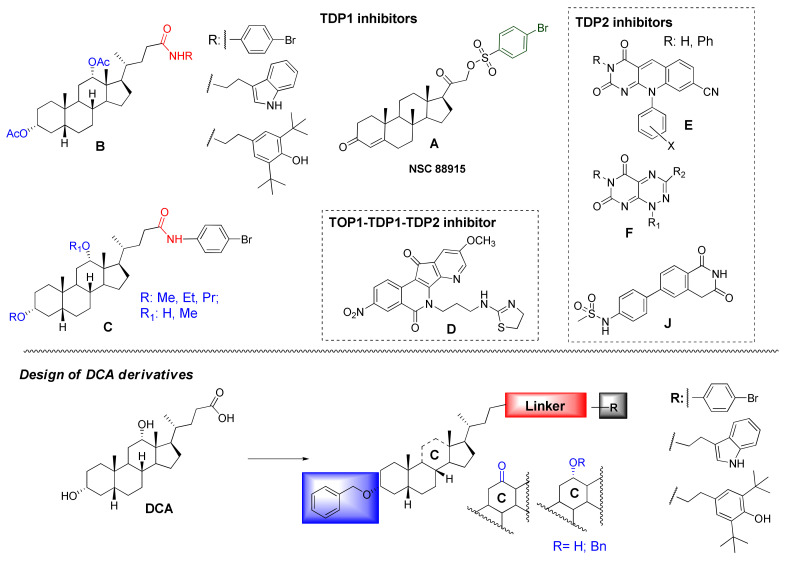
Structures of reported TDP1 and TDP2 inhibitors and design of DCA derivatives as inhibitors of these enzymes.

Several studies have reported the development of TDP1 inhibitors of organic chemical matter [6,7,8,9] as well as natural product derivatives: steroids (**A**–**C**) (Figure 1) [10,11,12], coumarins [13,14], usnic acids [15,16,17,18,19], and aminoadamantanes containing monoterpene-derived fragments [20,21,22,23]. Known TDP2 inhibitors are represented by isoquinoline-1,3-diones (**J**) [24], deazaflavins (**E**), and toxoflavins (**F**) [25]. Additionally, the indenoisoquinoline derivatives were discovered as a triple TOP1–TDP1–TDP2 inhibitor (**D**) [26] (Figure 1).

Previously, we discovered a new class of semisynthetic TDP1 inhibitors based on the bile acid scaffold [11,12]. It has been shown that 3,12-diacetoxy deoxycholic amides obtained using *para*-bromoaniline, 4-(2-aminoethyl)-2,6-bis-*t*-butylphenol, and tryptamine (**B**, Figure 1) as an amine component demonstrated the ability to inhibit TDP1 with IC_50_ values in the 0.29–0.47 μM range [11]. Additionally, it was found that *para*-bromoanilide derivatives of deoxycholic acid containing ether groups (methoxy, ethoxy and propyloxy) on the steroid framework demonstrated promising TDP1 inhibitory activity (IC_50_ = 0.27–0.65 μM) (**C**, Figure 1) [12].

Here, we have developed a set of deoxycholic acid derivatives as inhibitors of TDP1 and TDP2 containing benzyl ether groups instead of aliphatic ones on the steroid core and previously selected aromatic fragment (*para*-bromobenzene, indole, and 2,6-bis-*tert*-butylphenol) attached to the steroid via different linkers (amide, thioamide as well as 1,2,4- and 1,3,4-oxadiazole). The compounds were synthesized and tested against the TDP1 and TDP2 enzymes (Figure 1). Thus, we tried to establish the effect of bulk substituent attachment to both hydroxy groups of DCA as well as the influence of bioisosteric substitution of the amide moiety in linkers on inhibitory activity.

## 2. Results and Discussion

### 2.1. Chemistry

**Scheme 1:**
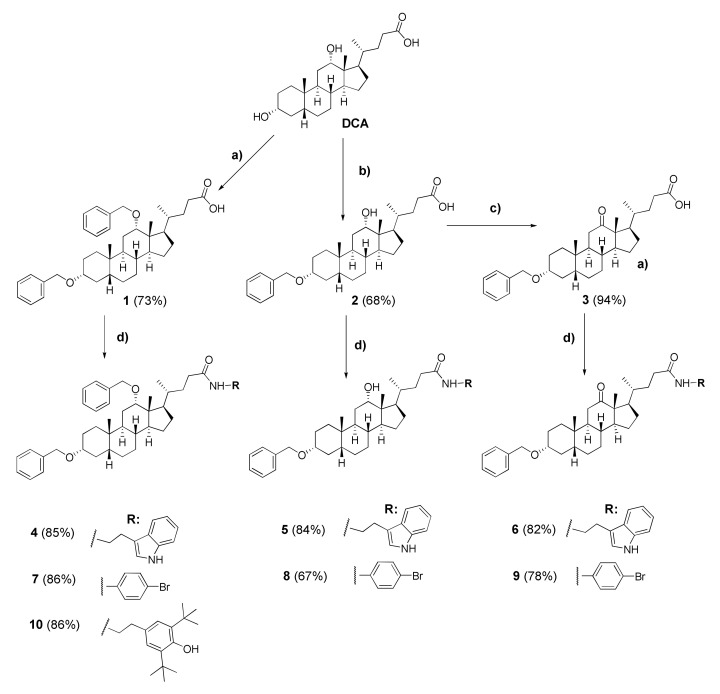
Synthesis of deoxycholic acid amides with the benzyloxy-group at C-3 and benzyloxy, hydroxy, and oxo-groups at C-12 on the steroid framework (**4**–**10**). Reagent and conditions: (**a**) benzyl bromide (4.5 eq.), NaH (~60% in oil), THF (reflux); (**b**) benzyl bromide (3.0 eq.), NaH (~60% in oil), THF (reflux); (**c**) Jones reagent, acetone (0 °C→RT); (**d**) (1) CDI, CH_2_Cl_2_, 2 h (RT), (2) corresponding amine, CH_2_Cl_2_, (RT→35 °C).

We synthesized a set of tryptamides (**4**–**6**) and *para*-bromoanilides (**7**–**9**) of DCA with the benzyloxy-group at C-3 and benzyloxy, hydroxy, and oxo-groups at C-12 on the steroid core (Scheme 1). At first, we modified the steroid framework by attaching the benzyloxy-group using DCA as the starting material for the synthesis. 3,12-*Bis*-benzyloxy derivative **1** was obtained by the reaction of DCA (1 mol eq.) with benzyl bromide (4.5 mol eq.) in THF under reflux with 73% yield. 3-Benzyloxy derivative **2** was obtained in a similar condition using 3-fold excess of benzyl bromide in THF under reflux with 68% yield after purification by column chromatography. 3-Benzyloxy-12-oxo derivative **3** was obtained by Jones oxidation of 12-hydroxy group in compound **2** with 94% yield. Subsequent carboxyl group transformation in compounds **1**–**3** into the amide group using preliminary activation with *N*,*N*′-carbonyldiimidazole (CDI) and subsequent reaction with appropriate amine led to the formation of tryptamides **4**–**6**, *para*-bromoanilides **7**–**9**, and compound **10** containing 2,6-di*-tert-*butylphenol moiety (yields 67–86%).

**Scheme 2:**
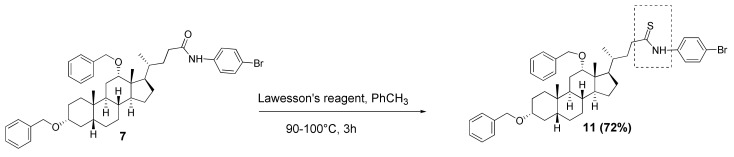
Synthesis of compound **11**.

**Scheme 3:**
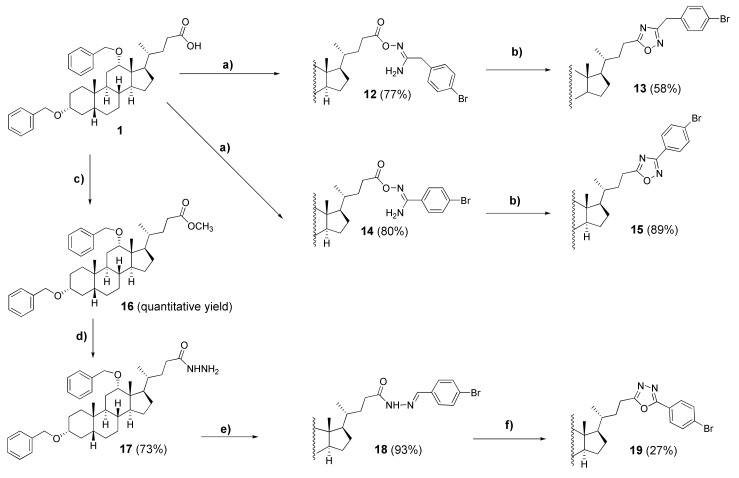
Synthesis of *bis*-benzyloxy derivatives of deoxycholic acid (**12**–**18**). Reagent and conditions: (**a**) (1) CDI, CH_2_Cl_2_ (RT); (2) corresponding amidoxime, overnight (RT); (**b**) *n-*Bu_4_NF, THF, 1h (reflux); (**c**) Me_2_SO_4_, acetone, K_2_CO_3_, KI, overnight (RT); (**d**) N_2_H_4_∙H_2_O, EtOH, 8 h (reflux); (**e**) *para*-bromobenzaldehyde, EtOH, 3 h (RT); (**f**) DMSO, I_2_, K_2_CO_3_, 3 h (70 °C).

To study the effect on activity of a bioisosteric substitution of the amide moiety in linkers between the *para*-bromophenyl moiety and the steroid core, we synthesized thioamide (**11**) **(Scheme 2**) as well as 1,2,4- (**13** and **15**) and 1,3,4-oxadiazole (**18**) derivatives (Scheme 3).

Thioamide **11** was obtained by reaction of compound **7** with the Lawesson’s reagent in dry toluene at 90 °C with 72% yield. 1,2,4-Oxadiazoles (**13** and **15**) were obtained using the following strategy: (1) acylation of corresponding amidoximes with activated carboxylic group of steroid compound **1**; (2) cyclization of *O*-acylated amidoximes. The amidoximes (4-bromo-*N’*-hydroxybenzimidamide and (2-(4-bromophenyl)-*N*’-hydroxyacetimidamide) were synthesized according to the literature [27] from corresponding nitriles and hydroxylamine. Activation of the carboxyl group in compound **1** with CDI and subsequent reaction with amidoximes containing the *para*-bromophenyl moiety led to the formation of intermediates **12** and **14** with 77% and 80% yields, respectively. 1,2,4-Oxadiazole derivatives **13** and **15** were formed by intermediates **12** and **14** cyclization in the presence of TBAF, an effective catalyst for the preparation of 1,2,4-oxadiazoles [28], under reflux in THF with 58% and 89% yields after purification by column chromatography.

1,3,4-Oxadiazole derivative **19** was synthesized by oxidative cyclization of hydrazone derivative **18** by iodine in the presence of potassium carbonate with 27% yield (Scheme 3). Intermediate **18** was obtained by reaction of 3,12-*bis*-benzyloxy hydrazide **17** with *para*-bromobenzaldehyde (yield 93%).

### 2.2. Biology

#### 2.2.1. Inhibition of Recombinant Enzymes TDP1 and TDP2

For real-time detection of TDP1 activity, we used the oligonucleotide biosensor based on the ability of TDP1 to remove fluorophore quenchers from the 3′-end of DNA [29]. The hexadecameric oligonucleotide carried 5(6)-carboxyfluorescein (FAM) at the 5′-end and fluorophore quencher BHQ1 (Black Hole Quencher-1) at the 3′-end. TDP1 inhibitors prevent the removal of fluorophore quenchers, thus reducing fluorescence intensity. The obtained IC_50_ values of compounds **4**–**18** are summarized in Table 1. We were unable to study the effect of replacing the amide group with a thioamide due to the instability of **11**. Most of the new DCA derivatives demonstrated good inhibitory activity with IC_50_ in the submicromolar range. These compounds suppressed the TDP1 activity slightly better than the reference compound furamidine [30].

We investigated the activity against the TDP2 enzyme for compounds **4–10**, **12–15**, and **17–19** (Table 1). TDP2 was tested for its ability to eliminate tyrosine residue from the 5’-end of the oligonucleotide substrate. The TDP2 reaction products were separated under denaturing conditions in polyacrylamide gel (PAGE). It was shown that the amides **4**–**10**, *O*-acylated amidoximes with modified steroid scaffold **12** and **14**, a hydrazone derivative **18** as well as a 1,3,4-oxadiazole derivative **19** inhibited the TDP2 enzyme with residual activity less than 50% at 1 mM concentration (Table 1). The 1,2,4-oxadiazole containing derivatives **13** and **15** were inactive. Thus, comparing the activity of oxadiazole derivatives **13**, **15**, and **19**, it is clear that the positioning of the *para*-bromanilide group is a determining factor, which can be used for the further development of dual and/or selective inhibitors. Note that as different assays formats were used for TDP1 and TDP2 inhibition, the value of these assays cannot be directly compared. A paper on derivatives of usnic acid as effective TDP1 inhibitors and mild inhibitors of TDP2 was recently published [31]. In this case, the same assay was used and these usnic acid derivatives demonstrated activity against TDP2, similar to those observed for DCA derivatives. More inhibitors of this enzyme have also been reported in the literature [24,25,26]. Obviously, further work for increasing activity against TDP2 is needed.

#### 2.2.2. The Intrinsic Cytotoxicity of the Compounds and Their Effect on the Cytotoxicity of Topotecan and Etoposide

**Figure 2:**
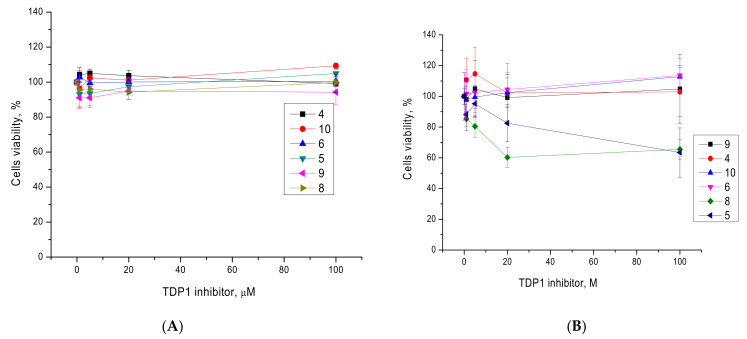
The influence of the TDP1 inhibitors on HEK293A (**A**) and HeLa (**B**) cell viability. The metabolic activity of the cells after 72 h of treatment according to the EZ4U assay.

Next, we examined the intrinsic cytotoxicity of the compounds on HeLa cervical cancer cells and human embryonic kidney derived cells HEK293A. Since the ligands are used to sensitize the action of topoisomerase inhibitors, their low intrinsic toxicity is vital. In this paper, we decided to concentrate on studying the cytotoxicity of compounds with the amide linker. Thus, the cytotoxicity of **4**–**6**, **8**–**10** was studied and they were non-toxic for HEK293A cells. Furthermore, **4**, **6**, **9**, and **10** were also non-toxic on HeLa cells (Figure 2). Compound **5** was non-toxic for non-cancerous HEK293A cells (cyan graph, Figure 2A), but caused 40% HeLa cell death at 100 μM (navy graph, Figure 2B). Compound **8** was also non-toxic for HEK293A cells (khaki, Figure 2A), but caused 40% HeLa cell death already at 20 μM while maintaining this level of toxicity at 100 μM (green, Figure 2B). This lack of correlation with concentration is probably due to aggregation, which is known to occur for bile acids [32]. Compared to the well-known cytostatics topotecan and etoposide, deoxycholic acid derivatives are relatively low-toxic: semi-toxic concentrations for the latter are from 40 μM and higher, while etoposide works at micromolar concentrations, and topotecan at submicromolar ones. Since the synthesized compounds are supposed to be used as tumor sensitizers to the action of known chemotherapy drugs, their low intrinsic cytotoxicity is a serious advantage, since it allows us to hope for the absence of additional side effects.

**Figure 3:**
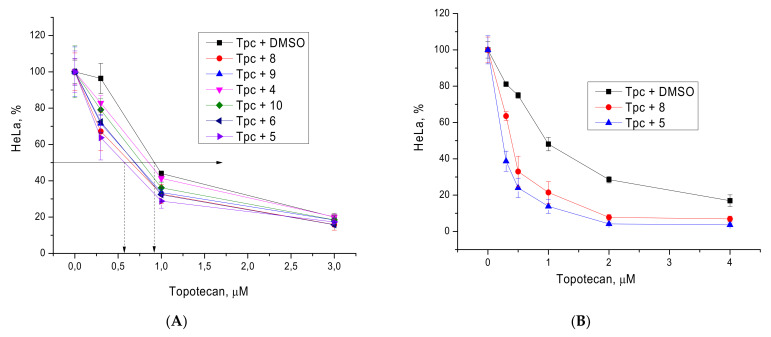
(**A**) The influence of derivatives **4**–**6** and **8**–**10** (5 μM) on topotecan cytotoxicity against HeLa cells. The error bars show standard deviations. Dotted lines show an increase in the cytotoxicity of topotecan in the presence of the ligands. (**B**) The influence of compounds **5** and **8** at 10 μM on topotecan cytotoxicity.

The inhibitors were studied, using their non-toxic concentration, to determine the cytotoxicity of topotecan, which is widely used in the treatment of cancer [33]. We used non-toxic concentrations of DCA derivatives **4**–**6** and **8**–**10** (up to 10 μM) at different concentrations of topotecan and assessed the cytotoxicity of the latter in the presence and absence of TDP1 inhibitors. The results are shown in Figure 3.

Compounds **5** and **8** had the greatest sensitizing effect at 5 μM, although it was not pronounced (Figure 3A). The concentration of these two compounds was increased to 10 μM and a more distinct effect was observed (Figure 3B). The combination index (CI) for different concentrations of topotecan and **5** at 10 μM was in the range 0.26–0.44, and for **8** at 10 μM, it was 0.18–0.30 (Supplementary Materials, Tables S1 and S2). CI value <1 indicates a synergistic effect of topotecan and compounds **5** and **8** [34].

Next, we studied the effect of the compounds on the cytotoxicity of TOP2 poison in the etoposide. In non-toxic concentrations, deoxycholic acid derivatives did not have a sensitizing effect (Figure S2). Obviously, to obtain sensitization requires the development of more effective inhibitors of TDP2.

Thus, DCA derivatives **5** and **8** containing the benzyloxy substituent at the C-3 position and non-substituted hydroxyl group at C-12 appeared to be inhibitors of both TDP1 and TDP2 enzymes and enhanced the cytotoxicity of topotecan at non-toxic concentration. Both compounds are promising for further development.

### 2.3. Molecular Modeling

DCA derivatives **1**–**18** were docked into the binding site of the TDP1 (PDB ID: 6W7K, resolution 1.70 Å) [35] and TDP2 (PDB ID: 5J3S, resolution 3.40 Å) [36] enzymes. The robustness of the TDP1 docking scaffold has been previously established [19]; the robustness of the TDP2 docking scaffold was confirmed and further discussion (see Supplementary Materials, Molecular modeling section). 

**Figure 4:**
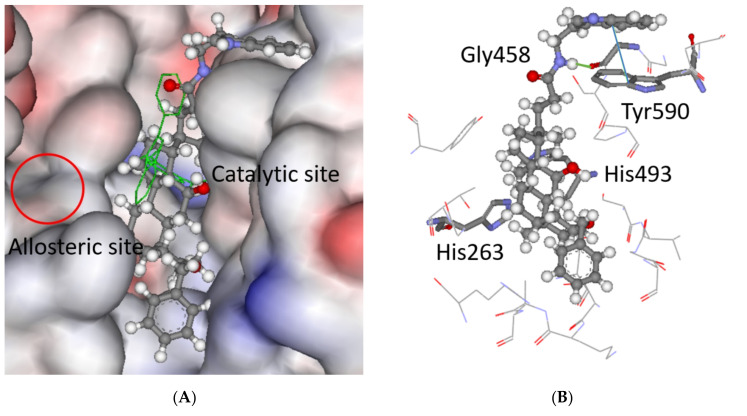
The docked pose of compound **5** in the catalytic site of TDP1 as predicted by the ChemScore scoring function. (**A**) The ligand is shown in the ball-and-stick format. The catalytic pocket is to the right-hand site, the co-crystallized ligand is depicted as green lines. The allosteric pocket is not predicted to be occupied (see circled area). The protein surface is rendered; blue depicts regions with a partial positive charge on the surface; red depicts regions with a partial negative charge and grey shows neutral areas. (**B**) The predicted configuration depicted as balls-and-sticks with hydrogen bonding to the backbone carbonyl of Gly458 (1.8 Å—green line) and π–π stacking between the indolyl rings in the ligand and Tyr590 (4.5 Å—blue line). These amino acid residues are shown as sticks as well as the catalytic His263 and His493. The adjacent amino acids (<5 Å), buttressing the ligand, are shown as lines. The hydrogens on the amino acids are not shown for clarity.

The binding mode of the most TDP1 active compound **5** was investigated. The steroid core is placed in the catalytic binding site with the indolyl and benzyloxy moieties pointing out of it. Molecular dynamics simulations have suggested that the TDP1 inhibitors occupy an allosteric binding pocket next to the catalytic site, as shown in Figure 4A [18]. The existence of the allosteric site was further supported by combined molecular modeling and structural activity relationship study of usnic acid derivatives, combined with monoterpenoids [19]. The occupancy of the allosteric site was shown to be very advantageous to the overall binding efficacy. As can be seen in Figure 4A, the modeling does not predict the occupancy of the allosteric site, and the spatial arrangement of the indolyl and benzyloxy rings on the steroid scaffold does not facilitate it. Rather, the ring systems are placed in positions that do not facilitate the optimal. Ligand **5** is anchored into the catalytic pocket by one hydrogen bond to the backbone of Gly458; furthermore π–π stacking between the indolyl rings of the ligand and Tyr590 is predicted (Figure 4B). In general, the other ligands follow this binding mode. Interestingly, derivatives with a benzyloxy ring on the steroid C ring (e.g., ligand **16**) have this moiety consistently docked into the same groove, but without any specific interactions with the adjacent amino acids.

An interesting consideration is the addition of the benzyloxy moieties compared to their acetoxy counterparts previously reported [11,12]. Three sets can be easily identified (see Table S7) (i.e., ligand **4** compared to **S7-1**, **7** compared to **S7-2**, and **10** compared to **S7-3**). According to the modeling reported by Salomatina et al. [11], the tethered conjugated ring systems of DCA derivatives **S7-1**, **S7-2**, and **S7-3** reach the allosteric site, resulting in good efficacy. As shown here, the addition of the benzyloxy moieties prevents the binding to the allosteric site. This deficiency is partially remedied by very high log *P* values of the ligands reported here (**4**—10.2 vs. **S7-1**—5.3; **7**—10.8 vs. **S7-2**—6.5; **10**—12.5 vs. **S7-3**—9.0). However, excessive lipophilicity cannot be recommended due to unspecific modulation of various enzymes, lack of water solubility, etc. Another example is the substitution of methoxy groups of ligand **S8-1** previously reported [12] with benzyloxy moieties on ligand **7** presented in this paper (Table S8). According to the modeling by Salomatina et al. [12], the bromobenzyl ring occupies the same cleft as the benzyloxy system on the A ring in the steroid scaffold (Figure 4A) (i.e., the resulting substitution changes the binding mode of the ligand). Furthermore, when Figure 2A in Salomatina et al. [12] is considered, the tethered bromobenzyl can easily reach the allosteric site. Again, the very high log *P* of derivative **7** (10.8) reported here increases its binding potency compared to **S8-1** (log *P*—7.4).

**Figure 5:**
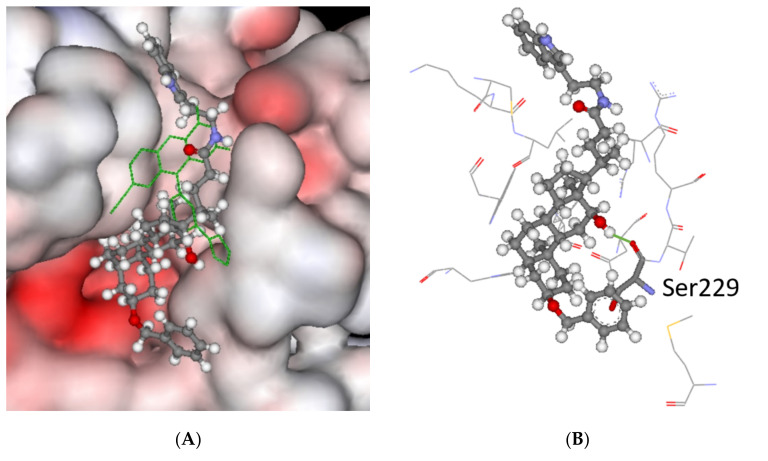
The docked pose of **5** in the catalytic site of TDP2 as predicted by the ChemPLP scoring function. (**A**) The ligand is shown in the ball-and-stick format. The co-crystallized ligand is shown (green lines). The protein surface is rendered; blue depicts regions with a partial positive charge on the surface; red depicts regions with a partial negative charge and grey shows neutral areas. (**B**) The predicted configuration depicted as balls-and-sticks with hydrogen bonding to Ser229 (1.9 Å—green line) shown as sticks. The amino acids (<5 Å) making up the binding pocket are shown as lines. The hydrogens on the amino acids are not shown for clarity.

The docking of compound **5** into the binding site of TDP2 can be seen in Figure 5A, where the predicted pose completely overlaps the co-crystalized ligand 6FQ occupying the same binding pocket. One hydrogen bond is predicted between **5** and the enzyme as shown in Figure 2B. The backbone carbonyl group of Ser229 is bound to the hydroxyl in **5**. The same binding mode is predicted for the other ligands irrespective of them being active or inactive (e.g., ligand **16**).

### 2.4. Prediction of Physicochemical and Drug-Like Properties

The calculated molecular descriptors MW (molecular weight), log *P* (water-octanol partition coefficient), HD (hydrogen bond donors), HA (hydrogen bond acceptors), PSA (polar surface area), and RB (rotatable bonds)) are given in Table S5. The values of the molecular descriptors lie within lead-like chemical space for HD, in drug-like space for HA and PSA, RB lies in drug-like and Known Drug Space (KDS). Finally, MW and log *P* span drug-like, KDS, and beyond, in other words, some of these values are very high (for the definition of lead-like, drug-like and KDS regions see [37] and Table S6). When the descriptor values were correlated with their TDP1 IC_50_ counterparts, modest negative correlation was seen with RB, HD, HA, and PSA (i.e., molecular descriptors linked to improved water solubility are negatively affected by larger values) (Table S3). Interestingly, no correlation was seen for log *P*. Correlations with HA, PSA, and RB have been previously observed for TDP1 inhibitors [12,20]. The averages of the descriptors were derived for the TDP2 active (+) and inactive (−) ligands; no statistical difference was seen between the two cohorts.

The Known Drug Indexes (KDIs) for the ligands were calculated to gauge the balance of the molecular descriptors (MW, log P, HD, HA, PSA, and RB). This method is based on the analysis of drugs in clinical use (i.e., the statistical distribution of each descriptor is fitted to a Gaussian function and normalized to 1, resulting in a weighted index). Both the summation of the indexes (KDI_2a_) and multiplication (KDI_2b_) methods were used [38] as shown for KDI_2a_ in Equation (1) and for KDI_2b_ in Equation (2); the numerical results are given in Table S5.

KDI_2a_ = I_MW_ + I_log P_ + I_HD_+ I_HA_ + I_RB_ + I_PSA_ (1)

KDI_2b_ = I_MW_ × I_log P_ × I_HD_× I_HA_ × I_RB_ × I_PSA_ (2)

The KDI_2a_ values for the ligands range from 2.48 to 5.57 with a theoretical maximum of 6 and the average of 4.08 (±1.27) for known drugs. The KDI_2b_ range is from 0.00 to 0.63, with a theoretical maximum of 1 and with KDS average of 0.18 (±0.20). The KDI_2a/2b_ were quite low, only the DCA reference compound had good values; almost all the ligands had a KDI_2b_ of 0 due to their large MW and log *P* values.

## 3. Materials and Methods

### 3.1. Chemistry. General Experimental Procedures

Melting points were determined on a METTLER TOLEDO FP900 thermosystem and uncorrected. The elemental composition of the products was determined from high-resolution mass spectra recorded on a DFS (double focusing sector) Thermo Electron Corporation instrument. Optical rotations were measured with a PolAAr 3005 polarimeter. Elemental analyses were determined with a EURO EA3000 automated CHNS-analyzer. Analyses indicated by the symbols of the elements were within ±0.4% of theoretical values. ^1^H and ^13^C NMR spectra were measured on Bruker spectrometers: AV-600 (operating frequency 600.30 MHz for ^1^H and 150.95 MHz for ^13^C), DRX-500 (500.13 MHz for ^1^H and 125.76 MHz for ^13^C), AV-400 (400.13 MHz for ^1^H and 100.61 MHz for ^13^C), and AV-300 (300.13 MHz for ^1^H and 75.47 MHz for ^13^C). Solutions of each compound were prepared in CDCl_3_. Chemical shifts were recorded in *δ* (ppm) using *δ* 7.24 (^1^H NMR) and *δ* 76.90 (^13^C NMR) of CHCl_3_ as internal standards. Chemical shift measurements were given in ppm and the coupling constants (*J*) in hertz (Hz). The hydrogen or carbon atom assignments marked with the same symbols *, ^#^, ^§^, ^‡^, ^†^, ^∞^, or ^¢^ are interchangeable. The purity of the final compounds and intermediates for biological testing was >98% as determined by HPLC analysis. HPLC analyses were carried out on a MilichromA-02 using a ProntoSIL 120-5-C18 AQ column (BISCHOFF, 2.0 × 75 mm column, grain size 5.0 µm). The mobile phase was Millipore purified water with 0.1% trifluoroacetic acid at a flow rate of 150 µL/min at 35 °C with UV detection at 210, 220, 240, 260, and 280 nm. A typical run time was 25 min with a linear gradient of 0–100% methanol. Flash column chromatography was performed with silica gel (Merck, 60–200 mesh). All courses of all reactions were monitored by TLC analysis using Merck 60 F254 silica gel (Merck KGaA, Darmstadt, Germany) on aluminum sheets with the eluent CHCl_3_; CHCl_3_–MeOH (25:1.5). The carbon atom numbering of all compounds is given in the Supplementary Materials. The carbon atom numbering for compound 12 was different for the compound name and NMR assignments in the experimental section.

### 3.2. Reagents

*N*,*N*′-Carbonyldiimidazole (CDI), tryptamine, NaH (60% in mineral oil), Lawesson’s reagent, and 4-bromophenylacetonitrile were purchased from Acros Organics (Acros Organics B.V.B.A., Geel, Belgium). Benzyl bromide was purchased from Alfa Aesar. 3-Acetylaniline and *n*-Bu_4_NF were purchased from Sigma Aldrich. 4-Acetylaniline was purchased from J&K. Deoxycholic acid (DCA) was purchased from Abcr GmbH & Co. KG. Dimethyl sulfate was purchased from VEKTON. 4-Bromobenzonitrile and 4-bromobenzaldehyde were purchased from Fluorochem. All solvents used in the reactions were purified and dried according to previously reported procedures. 4-Bromo*-N′*-hydroxybenzimidamide and 2-(4-bromophenyl)-*N′*-hydroxyacetimidamide were prepared according to the method in the literature [27].

#### 3.2.1. 3α,12α-Dibenzyloxy-5β-cholan-24-oic acid (**1**)

Mixture of DCA (0.6 g, 1.53 mmol) and NaH (57–63% in oil; 0.37 g, 9.18 mmol) in THF (10 mL) was heated at 50 °C for 1 h. Then, benzyl bromide (0.56 mL, 4.59 mmol) was added, and the reaction mixture was refluxed for 24 h. Next, NaH (57–63% in oil; 0.18 g, 4.60 mmol) and benzyl bromide (0.27 mL, 2.30 mmol) were added to the reaction mixture, and the reaction mixture was refluxed until full conversion was reached. The reaction course was monitored by TLC. The reaction mixture was cooled to room temperature, concentrated under vacuum, diluted with AcOEt–CHCl_3_ mixture, washed with aqueous NH_4_Cl, and dried over MgSO_4_. Crude product (1.27 g) was purified by flash column chromatography (SiO_2_, CH_2_Cl_2_ then CHCl_3_) to give a pure sample of compound **1** (0.64 g, 73%) as a white amorphous solid. Mp 181.2 °C [decomposition]. [$\alpha_{D}^{23}$] +60 (*c* 0.20 g/100 mL; CHCl_3_). HRMS: Calc. for (C_38_H_52_O_4_)^+^ *m*/*z* = 572.3860; found *m*/*z* = 481.3314; calc. for (C_31_H_45_O_4_)^+^ *m*/*z* = 481.3312 [M–PhCH_2_]^+^. ^1^H NMR (CDCl_3_, 500 MHz): *δ* = 7.38–7.20 (m, 10H, aromatic protons), 4.59 (d, 1H, *J =* 11.4, H-26), 4.52 (s, 2H, CH_2_-25), 4.59 (d, 1H, *J =* 11.4, H-26′), 3.66 (s, 1H, H-12), 3.35 (m, 1H, H-3), 2.38 (m, 1H, H-23), 2.21 (m, 1H, H-23′), 2.02 (ddd, 1H, *J = J = J =* 9.6, H-17), 1.89–1.66 (m, 9H; H-4, H-9, H-20, H-6, H-22, H-14, 3H), 1.64–1.50 (m, 2H, H-4, H-15), 1.47–1.18 (m, 9H,H-8, H-5, H-22′, H-6′, H-7, 4H), 1.13 (m, 1H, H-7′), 1.03 (m, 1H, H-15′), 0.93 (m, 1H, H-1′), 0.91 (s, 3H, CH_3_-19), 0.87 (d, 3H, *J_21,20_* = 6.3, CH_3_-21), 0.69 (s, 3H, CH_3_-18). ^13^C NMR (CDCl_3_, 125 MHz): δ = 179.51 (s, C-24), 139.24 (s, C-1^Ph^ *), 139.17 (s, C-1^Ph′^ *), 128.16 (d, C-3^Ph^, C-5^Ph^, C-3^Ph′^, C-5^Ph′^), 127.45 (d, C-2^Ph^, C-6^Ph^, C-2^Ph′^, C-6^Ph′^), 127.37 (d, C-4^Ph #^), 127.14 (d, C-4^Ph′ #^), 80.91 (d, C-12), 78.54 (d, C-3), 70.20 (t, C-26), 69.50 (t, C-25), 48.64 (d, C-14), 46.54 (s, C-13), 46.13 (d, C-17), 42.17 (d, C-5), 36.06 (d, C-8), 34.29 (s, C-10^§^), 35.15 (d, C-20), 34.47 (t, C-1^§^), 33.74 (d, C-9), 33.20 (t, C-4), 30.75 (t, C-23), 30.70 (t, C-22), 27.46, (t, C-11^‡^), 27.31 (t, C-16^‡^), 27.21 (t, C-2^‡^), 25.97 (t, C-7), 23.64 (t, C-15), 23.26 (q, C-19), 23.02 (t, C-6), 17.37 (q, C-21), 12.66 (q, C-18).

#### 3.2.2. 3α-Benzyloxy-12α-hydroxy-5β-cholan-24-oic Acid (**2**) 

Mixture of DCA (1.0 g, 2.55 mmol) and NaH (57–63% in oil; 0.5 g, 11.9 mmol) in THF (20 mL) was heated at 50 °C for 3 h. Then, benzyl bromide (0.92 mL, 7.65 mmol) was added, and the reaction mixture was refluxed until full conversion was reached. The reaction course was monitored by TLC. The reaction mixture was cooled to room temperature, concentrated under vacuum, diluted with AcOEt–CHCl_3_ mixture, washed with aqueous NH_4_Cl, and dried over MgSO_4_. The crude product (1.9 g) was purified by flash column chromatography (SiO_2_, CHCl_3_) to give a pure sample of compound **2** (0.84 g, 68%) as a white amorphous solid. Mp 86.0 °C [decomposition]. [$\alpha_{D}^{21.5}$] +47 (*c* 0.20 g/100 mL; CHCl_3_). HRMS: *m*/*z* calcd. for (C_31_H_46_O_4_)^+^ 482.3391; found 482.3379. ^1^H NMR (CDCl_3_, 500 MHz): *δ* = 7.34–7.28 (m, 4H, H-2^Ph^, H-3^Ph^, H-5^Ph^, H-6^Ph^), 7.25 (m, 1H, H-4^Ph^), 4.55 (s, 2H, CH_2_-25), 3.92 (s, 1H, H-12), 3.39 (m, 1H, H-3), 2.40 (m, 1H, H-23), 2.18 (m, 1H, H-23′), 2.00–1.89 (m, 2H; H-4, H-9), 1.89–1.68 (m, 6H, H-16, H-6, H-17, H-22, H-1, H-2), 1.64–1.46 (m, 6H, H-4′, H-15, H-2′, H-14, CH_2_-11), 1.43–1.27 (m, 5H, H-8, H-7, H-22′, H-20, H-5), 1.27–1.00 (m, 4H, H-6′, H-16′, H-7′, H-15′), 0.96 (d, 3H, *J_21,20_* = 6.2, CH_3_-21), 0.87 (s, 3H, CH_3_-19), 0.87 (m, 1H, H-1′), 0.66 (s, 3H, CH_3_-18). ^13^C NMR (CDCl_3_, 125 MHz): δ = 178.43 (s, C-24), 138.63 (s, C-1^Ph^), 128.22 (d, C-3^Ph^, C-5^Ph^), 127.52 (d, C-2^Ph^, C-6^Ph^), 127.36 (d, C-4^Ph^), 78.85 (d, C-3), 73.17 (d, C-12), 69.80 (t, C-25), 48.30 (d, C-14), 47.50 (d, C-17), 46.52 (s, C-13), 42.04 (d, C-5), 35.85 (d, C-8), 35.46 (d, C-20), 35.19 (t, C-1), 34.30 (s, C-10), 33.14 (d, C-9), 32.69 (t, C-4), 31.07 (t, C-23), 30.67 (t, C-22), 28.46, (t, C-11), 27.56 (t, C-16), 27.05 (t, C-6), 26.60 (t, C-2), 25.94 (t, C-7), 23.60 (t, C-15), 22.81 (q, C-19), 17.04 (q, C-21), 12.60 (q, C-18).

#### 3.2.3. 3α-Benzyloxy-12α-oxo-5β-cholan-24-oic Acid (**3**)

To a solution of compound **2** (4.6 g, 9.5 mmol) in acetone (150 mL) Jones reagent (3 mL) was added dropwise. The reaction mixture was stirred for 2 h at room temperature. The reaction course was monitored by TLC. Then, EtOH (10 mL) was added and the reaction mixture was stirred for an extra 30 min. Precipitate was filtered, organic phase was concentrated under vacuum, diluted with water, extracted with CH_2_Cl_2_–Et_2_O, and dried over anhydrous MgSO_4_. Crude product (4.47 g, 98%) was purified by flash column chromatography (SiO_2_, 0–1% MeOH gradient in CHCl_3_) to give a pure sample of **3** (4.3 g, 94%). Mp 147.9°C (AcOEt) [decomposition]. [$\alpha_{D}^{26.5}$] +90 (*c* 0.20 g/100 mL; CHCl_3_). HRMS: *m*/*z* calcd. for (C_31_H_44_O_4_)^+^ 480.3234; found 480.3231. ^1^H NMR (CDCl_3_, 500 MHz): *δ* = 7.32–7.28 (m, 4H, H-2^Ph^, H-3^Ph^, H-5^Ph^, H-6^Ph^), 7.26–7.21 (m, 1H, H-4^Ph^), 4.52 (ddd, 2H, *J = J = J =* 10.7, CH_2_-25), 3.34 (m, 1H, H-3), 2.43 (dd, 1H, *J* = *J* = 12.5, H-11β), 2.41 (m, 1H, H-23), 2.27 (m, 1H, H-23′), 2.07–1.22 (m, 21H), 1.11 (m, 1H, H-7), 1.02–0.96 (m, 7H; H-1, 0.99 (s, 3H, CH_3_-18*), 0.98 (s, 3H, CH_3_-19*)), 0.84 (d, 3H, *J =* 6.6, CH_3_-21). ^13^C NMR (CDCl_3_, 125 MHz): δ = 214.49 (s, C-12), 179.30 (s, C-24), 138.94 (s, C-1^Ph^), 128.22 (d, C-3^Ph^, C-5^Ph^), 127.41 (d, C-2^Ph^, C-6^Ph^), 127.25 (d, C-4^Ph^), 77.95 (d, C-3), 69.69 (t, C-25), 58.44 (d, C-14), 57.38 (s, C-13), 46.35 (d, C-17), 43.88 (d, C-9), 41.52 (d, C-5), 37.97 (t, C-11), 35.62, 35.57, 35.50, 35.16, 33.16, 31.09, 30.20, 27.41, 27.13, 26.77, 25.94 (t, C-7), 24.2 (t, C-15), 22.71 (q, C-19), 18.44 (q, C-21), 11.59 (q, C-18).

#### 3.2.4. *N*-(2″-(1*H*-Indol-3′-yl)ethyl)-3α,12α-dibenzyloxy-5β-cholane-24-amide (**4**) 

Compound **1** (0.30 g, 0.52 mmol) and CDI (0.10 g, 0.62 mmol) was dissolved in CH_2_Cl_2_ (10 mL) and stirred at room temperature for 2 h. Then, tryptamine (0.10 g, 0.62 mmol) was added and the reaction mixture was stirred at 30–35 °C for 8 h. The reaction course was monitored by TLC (CHCl_3_). Reaction mixture was diluted with CH_2_Cl_2_ and Et_2_O, washed sequentially with H_2_O and brine, and dried over anhydrous MgSO_4_. Solvent was evaporated to dryness; crude product (0.40 g, quantitative yield) was purified by flash column chromatography (SiO_2_, *n*-hexane with gradient 10–50% AcOEt) to give a pure sample of compound **4** (0.32 g, 85%) as a white amorphous solid. Mp 72.0 °C [decomposition]. [$\alpha_{D}^{25.7}$] +52 (*c* 0.10 g/100 mL; CHCl_3_). HRMS: Calc. for (C_48_H_62_O_3_N_2_)^+^ *m*/*z* = 714.4755; found *m*/*z* = 714.4748. ^1^H-NMR (CDCl_3_, 500 MHz): *δ* = 8.16 (s, NH, indole), 7.59 (d, 1H, *J* = 7.7, H-5′), 7.41–7.06 (m, 13H, aromatic protons of Ph and Ph′, H-8′, H-7′, H-6′), 6.99 (br.s., 1H, H-2′), 5.43 (m, NH, amide), 4.59 (d, 1H, *J =* 11.4, H-26), 4.52 (m, CH_2_-25), 4.25 (d, 1H, *J =* 11.4, H-26′), 3.65 (s, 1H, H-12), 3.56 (m, 2H, CH_2_-1″), 3.33 (m, 1H, H-3), 2.94 (m, CH_2_-2″), 2.13 (m, 1H, H-23), 2.06–0.78 (m, 31H; 0.91 (s, 3H, CH_3_-19), 0.84 (d, 3H, *J =* 6.2, CH_3_-21)), 0.67 (s, 3H, CH_3_-18). ^13^C NMR (CDCl_3_, 125 MHz): δ = 173.47 (s, C-24), 139.22 (s, C-1^Ph^ *), 139.19 (s, C-1^Ph′^ *), 136.33 (s, C-9^), 128.16 (d, C-3^Ph^, C-5^Ph #^), 128.13 (C-3^Ph′^, C-5^Ph′ #^), 127.42 (d, C-2^Ph^, C-6^Ph ∞^), 127.36 (d, C-2^Ph′^, C-6^Ph′ ∞^), 127.28 (s, C-4^), 127.15 (d, C-4^Ph §^), 127.09 (d, C-4^Ph′ §^), 122.04 (d, C-2^), 121.87 (d, C-7^), 119.34 (d, C-6^), 118.60 (d, C-5^), 113.00 (s, C-3^), 111.13 (d, C-8^), 80.97 (d, C-12), 78.51 (d, C-3), 70.16 (t, C-26), 69.52 (t, C-25), 48.68 (d, C-14), 46.46 (s, C-13), 46.03 (d, C-17), 42.11 (d, C-5), 39.57 (t, C-1″), 35.99 (d, C-8), 35.26 (d, C-20), 35.23 (s, C-10^‡^), 34.43 (t, C-1^‡^), 33.72 (d, C-9), 34.37 (t, C-23), 33.19 (t, C-4), 31.64 (t, C-22), 27.50 (t, C-11^†^), 27.26 (t, C-16^†^), 27.19 (t, C-2^†^), 25.94 (t, C-7), 25.25 (t, C-2″), 23.61 (t, C-15), 23.25 (q, C-19), 22.97 (t, C-6), 17.46 (q, C-21), 12.62 (q, C-18).

#### 3.2.5. *N*-(2″-(1*H*-Indol-3′-yl)ethyl)-3α-benzyloxy-12α-hydroxy-5β-cholane-24-amide (**5**) 

Compound **2** (0.25 g, 0.52 mmol) and CDI (0.10 g, 0.62 mmol) was dissolved in CH_2_Cl_2_ (10 mL) and stirred at room temperature for 2 h. Then, tryptamine (0.10 g, 0.62 mmol) was added and reaction mixture was stirred at 30–35 °C for 36 h. The reaction course was monitored by TLC (CHCl_3_–AcOEt, 15:10). Reaction mixture was diluted with CH_2_Cl_2_ and Et_2_O, washed sequentially with H_2_O, HCl 5% (aq), NaHCO_3_ (aq), brine, and dried over anhydrous MgSO_4_. Solvent was evaporated to dryness; crude product (0.35 g, quantitative yield) was purified by flash column chromatography (SiO_2_, CH_2_Cl_2_ with gradient 10–30% AcOEt) to give a pure sample of compound **5** (0.27 g, 84%) as a white amorphous solid. Mp 81.7 °C [decomposition]. [$\alpha_{D}^{23.7}$] +40 (*c* 0.10 g/100 mL; CHCl_3_). HRMS: Calc. for (C_41_H_56_O_3_N_2_)^+^ *m*/*z* = 624.4286; found *m*/*z* = 624.4285. ^1^H-NMR (CDCl_3_, 300 MHz): *δ* = 8.54 (br.s., NH, indole), 7.63–7.53 (d, 1H, *J* = 7.7, H-5^), 7.42–7.21 (m, 5H, H-2^Ph^, H-3^Ph^, H-4^Ph^, H-5^Ph^, H-6^Ph^), 7.21–7.04 (m, 2H, H-7^, H-6^), 6.99 (m, 1H, H-2^), 5.69 (m, NH, amide), 4.55 (s, 2H, CH_2_-25), 3.89 (s, 1H, H-12), 3.56 (m, 2H, CH_2_-1″), 3.37 (m, 1H, H-3), 2.94 (m, 2H, H-2″), 2.14–0.79 (m, 33H, 2.14 (m, 1H, H-23), 1.97 (m, 1H, H-23′), 0.90 (d, 3H, *J =* 6.2, CH_3_-21), 0.88 (s, 3H, CH_3_-19)), 0.62 (s, 3H, CH_3_-18). ^13^C NMR (CDCl_3_, 125 MHz): δ = 173.54 (s, C-24), 138.85 (s, C-1^Ph^), 136.25 (s, C-9^), 128.21 (d, C-3^Ph^, C-5^Ph^), 127.45 (d, C-2^Ph^, C-6^Ph^), 127.26 (d, C-4^Ph^), 127.17 (s, C-4^), 122.06 (d, C-2^), 121.86 (d, C-7^), 119.16 (d, C-6^), 118.50 (d, C-5^), 112.68 (s, C-3^), 111.22 (d, C-8^), 78.44 (d, C-3), 72.92 (d, C-12), 69.72 (t, C-25), 48.05 (d, C-14), 46.83 (d, C-17), 46.24 (s, C-13), 41.85 (d, C-5), 39.50 (t, C-1″), 35.78 (d, C-8), 35.00 (s, C-10*), 34.96 (d, C-20), 34.24 (t, C-1*), 33.43 (d, C-9), 33.26 (t, C-23^#^), 32.98 (t, C-4^#^), 31.45 (t, C-22), 28.48 (t, C-11), 27.25 (t, C-16^§^), 27.03 (t, C-6^§^), 26.91 (t, C-2^§^), 25.87 (t, C-7), 25.14 (t, C-2″), 23.45 (t, C-15), 23.02 (q, C-19), 17.20 (q, C-21), 12.60 (q, C-18).

#### 3.2.6. *N*-(2″-(1*H*-Indol-3′-yl)ethyl)-3α-benzyloxy-12-oxo-5β-cholane-24-amide (**6**) 

Compound **3** (0.3 g, 0.62 mmol) and CDI (0.12 g, 0.75 mmol) was dissolved in CH_2_Cl_2_ (10 mL) and stirred at room temperature for 2 h. Then, tryptamine (0.12 g, 0.75 mmol) was added and the reaction mixture was stirred at 30–35 °C overnight. The reaction course was monitored by TLC (CHCl_3_–AcOEt, 20:3). Reaction mixture was diluted with CH_2_Cl_2_ and Et_2_O, washed sequentially with H_2_O and brine, and dried over anhydrous MgSO_4_. Solvent was evaporated to dryness; crude product (0.42 g, quantitative yield) was purified by flash column chromatography (SiO_2_, *n*-hexane with gradient 20–50% AcOEt) to give a pure sample of compound **6** (0.32 g, 82%) as a white amorphous solid. Mp 87.5 °C [decomposition]. [$\alpha_{D}^{25.7}$] +70 (*c* 0.10 g/100 mL; CHCl_3_). HRMS: Calc. for (C_41_H_54_O_3_N_2_)^+^ *m*/*z* = 622.4129; found *m*/*z* = 622.4124. ^1^H-NMR (CDCl_3_, 500 MHz): *δ* = 8.42 (s, NH, indole), 7.62–7.54 (m, 1H, H-5^), 7.37–7.21 (m, 6H, H-8^, H-2^Ph^, H-3^Ph^, H-4^Ph^, H-5^Ph^, H-6^Ph^), 7.17 (m, 1H, H-7^), 7.09 (m, 1H, H-6^), 6.99 (br.s., 1H, H-2^), 5.62 (m, NH, amide), 4.52 (ddd, 2H, *J = J = J =* 10.2, CH_2_-25), 3.57 (m, 2H, CH_2_-1″), 3.35 (m, 1H, H-3), 2.95 (m, 2H, CH_2_-2″), 2.43 (dd, 1H, *J = J =* 12.5, H-11β), 2.17 (m, 1H, H-23), 2.08–0.89 (m, 30H, 0.98 (s, 3H, CH_3_-18*), 0.96 (s, 3H, CH_3_-19*)), 0.80 (d, 3H, *J =* 6.5, CH_3_-21). ^13^C NMR (CDCl_3_, 125 MHz): δ = 214.65 (s, C-12), 173.30 (s, C-24), 138.88 (s, C-1^Ph^), 136.33 (s, C-9^), 128.19 (d, C-3^Ph^, C-5^Ph^), 127.37 (d, C-2^Ph^, C-6^Ph^), 127.24 (d, C-4^Ph^), 121.92 (d, C-2^, C-7^), 119.21 (d, C-6^), 118.53 (d, C-5^), 112.81 (s, C-3^), 111.16 (d, C-8^), 77.92 (d, C-3), 69.69 (t, C-25), 58.44 (d, C-14), 57.34 (s, C-13), 46.22 (d, C-17), 43.87 (d, C-9), 41.44 (d, C-5), 39.55 (t, C-1″), 37.95 (t, C-11), 35.52 (d, C-8), 35.49 (s, C-10^§^), 35.43 (d, C-20), 35.09 (t, C-1^§^), 33.56 (t, C-23), 33.10 (t, C-4), 31.02 (t, C-22), 27.29, 27.05, 26.74, 25.87 (t, C-7), 25.22 (t, C-2″), 24.15, 22.64 (q, C-19), 18.59 (q, C-21), 11.55 (q, C-18).

#### 3.2.7. *N*-(4′-Bromophenyl)-3α,12α-dibenzyloxy-5β-cholane-24-amide (**7**) 

Compound **1** (0.39 g, 0.68 mmol) and CDI (0.33 g, 2.0 mmol) was dissolved in CH_2_Cl_2_ (10 mL) and stirred at room temperature for 2 h, then the solution was heated to 30–35 °C and stirred 1 h more. Next, 4-bromoaniline (0.15 g, 0.89 mmol) was added and the reaction mixture was stirred at 30–35 °C for 2 h. The reaction course was monitored by TLC. Reaction mixture was diluted with AcOEt, washed sequentially with H_2_O, HCl 5% (aq), NaHCO_3_ (aq), brine, and dried over anhydrous MgSO_4_. Solvent was evaporated to dryness; crude product (0.52 g, quantitative yield) was purified by flash column chromatography (SiO_2_, CH_2_Cl_2_) to give a pure sample of compound **7** (0.42 g, 86%) as a white amorphous solid. Mp 186.2–191.3 °C. [$\alpha_{D}^{21.5}$] +20 (*c* 0.20 g/100 mL; CHCl_3_). HRMS: Calc. for (C_44_H_56_O_3_N^79^Br)^+^ *m*/*z* = 725.3438; found *m*/*z* = 725.3444. ^1^H-NMR (CDCl_3_, 400 MHz): *δ* = 7.44–7.21 (m, 15H, all aromatic protons, NH), 4.61 (d, 1H, *J =* 11.5, H-26), 4.52 (s, CH_2_-25), 4.27 (d, 1H, *J =* 11.5, H-26′), 3.67 (s, 1H, H-12), 3.36 (m, 1H, H-3), 2.33 (m, 1H, H-23), 2.16 (m, 1H, H-23′), 2.02 (m, 1H, H-17), 1.91–0.83 (m, 29H, 0.92 (s, 3H, CH_3_-19), 0.89 (d, 3H, *J =* 5.4, CH_3_-21)), 0.69 (s, 3H, CH_3_-18). ^13^C NMR (CDCl_3_, 100 MHz): δ = 171.94 (s, C-24), 139.18 (s, C-1^Ph^ *), 139.02 (s, C-1^Ph′^ *), 136.94 (s, C-1^), 131.68 (d, C-3^, C-5^), 128.13 (d, C-3^Ph^, C-5^Ph #^), 128.11 (C-3^Ph′^, C-5^Ph′ #^), 127.33 (d, C-2^Ph^, C-6^Ph^, C-2^Ph′^, C-6^Ph′^), 127.14 (d, C-4^Ph §^), 127.07 (d, C-4^Ph′ §^), 121.20 (d, C-2^, C-6^), 116.40 (s, C-4^), 80.88 (d, C-12), 78.42 (d, C-3), 70.03 (t, C-26), 69.46 (t, C-25), 48.62 (d, C-14), 46.39 (s, C-13), 45.90 (d, C-17), 41.99 (d, C-5), 35.89 (d, C-8), 35.14 (s, C-10^‡^), 35.09 (d, C-20), 34.36 (t, C-1^‡^), 34.05 (t, C-23^†^), 33.62 (d, C-9), 33.08 (t, C-4), 31.26 (t, C-22^†^), 27.46, 27.31, 27.21, 25.87 (t, C-7), 23.55 (t, C-15), 23.22 (q, C-19), 22.86 (t, C-6), 17.43 (q, C-21), 12.61 (q, C-18).

#### 3.2.8. *N*-(4′-Bromophenyl)-3α-benzyloxy-12α-hydroxy-5β-cholane-24-amide (**8**) 

Compound **2** (0.35 g, 0.73 mmol) and CDI (0.35 g, 2.2 mmol) was dissolved in CH_2_Cl_2_ (10 mL) and stirred at room temperature for 2 h, then, the solution was heated to 30–35 °C and stirred 1 h more. Next, 4-bromoaniline (0.20 g, 1.16 mmol) was added and the reaction mixture was stirred at 30–35 °C for 2 h. The reaction course was monitored by TLC. The reaction mixture was diluted with AcOEt, washed sequentially with H_2_O, HCl 5% (aq), NaHCO_3_ (aq), brine, and dried over anhydrous MgSO_4_. Solvent was evaporated to dryness; crude product (0.44 g, 96%) was purified by flash column chromatography (SiO_2_, CH_2_Cl_2_ with gradient 0–1.0% MeOH) to give compound **8** (0.08 g) and a pure sample of compound **8** (0.24 g, 67%) as a white amorphous solid. Mp 95.6–102.3 °C. [$\alpha_{D}^{21.5}$] +29 (*c* 0.20 g/100 mL; CHCl_3_). HRMS: Calc. for (C_37_H_50_O_3_N^79^Br)^+^ *m*/*z* = 635.2969; found *m*/*z* = 635.2963. ^1^H-NMR (CDCl_3_, 500 MHz): *δ* = 7.58 (br.s., 1H, NH), 7.42–7.35 (m, 4H, H-2^, H-3^, H-5^, H-6^), 7.33–7.28 (m, 4H, H-2^Ph^, H-3^Ph^, H-5^Ph^, H-6^Ph^), 7.27–7.21 (m, 1H, H-4^Ph^), 4.53 (s, 2H, CH_2_-25), 3.93 (s, 1H, H-12), 3.36 (m, 1H, H-3), 2.36 (m, 1H, H-23), 2.21 (m, 1H, H-23′), 2.00–0.83 (m, 31H; 0.96 (d, 3H, *J =* 5.9, CH_3_-21), 0.88 (s, 3H, CH_3_-19)), 0.64 (s, 3H, CH_3_-18). ^13^C NMR (CDCl_3_, 125 MHz): δ = 171.92 (s, C-24), 138.81 (s, C-1^Ph^), 137.04 (s, C-1^), 131.72 (d, C-3^, C-5^), 128.22 (d, C-3^Ph^, C-5^Ph^), 127.47 (d, C-2^Ph^, C-6^Ph^), 127.29 (d, C-4^Ph^), 121.20 (d, C-2^, C-6^), 116.39 (s, C-4^), 78.42 (d, C-3), 72.99 (d, C-12), 69.73 (t, C-25), 48.10 (d, C-14), 46.76 (d, C-17), 46.30 (s, C-13), 41.89 (d, C-5), 35.83 (d, C-8), 35.01, 34.90 (d, C-20), 34.29, 34.03, 33.47, 33.00, 31.15, 28.61, 27.31, 27.06, 26.94, 25.92 (t, C-7), 23.49 (t, C-15), 23.06 (q, C-19), 17.30 (q, C-21), 12.64 (q, C-18). 

#### 3.2.9. *N*-(4′-Bromophenyl)-3α-benzyloxy-12-oxo-5β-cholane-24-amide (**9**) 

Compound **3** (1.0 g, 2.08 mmol) and CDI (0.4 g, 2.5 mmol) was dissolved in CH_2_Cl_2_ (15 mL) and stirred at room temperature for 2 h, then, the solution was heated to 30–35 °C and stirred 1 h more. Next, 4-bromoaniline (0.43 g, 2.5 mmol) was added and the reaction mixture was stirred at 30–35 °C overnight. The reaction course was monitored by TLC. Reaction mixture was diluted with AcOEt, washed sequentially with H_2_O, HCl 5% (aq), NaHCO_3_ (aq), brine, and dried over anhydrous MgSO_4_. Solvent was evaporated to dryness; crude product (1.38 g, quantitative yield) was purified by flash column chromatography (SiO_2_, CH_2_Cl_2_) to give a pure sample of compound **9** (1.03 g, 78%) as a white amorphous solid. Mp 90.2 °C [decomposition]. [$\alpha_{D}^{25.7}$] +58 (*c* 0.10 g/100 mL; CHCl_3_). HRMS: Calc. for (C_37_H_48_O_3_N^79^Br)^+^ *m*/*z* = 633.2812; found *m*/*z* = 633.2824. ^1^H-NMR (CDCl_3_, 500 MHz): *δ* = 7.43–7.37 (m, 4H, H-2^, H-3^, H-5^, H-6^), 7.36–7.28 (m, 5H, NH, H-2^Ph^, H-3^Ph^, H-5^Ph^, H-6^Ph^), 7.27–7.21 (m, 1H, H-4^Ph^), 4.52 (ddd, 2H, *J = J = J =* 10.4, CH_2_-25), 3.34 (m, 1H, H-3), 2.44 (dd, 1H, *J = J =* 12.6, H-11β), 2.41 (m, 1H, H-23), 2.27 (m, 1H, H-23′), 2.07–1.98 (m, 2H), 1.97–1.58 (m, 10H), 1.52–1.39 (m, 3H), 1.39–1.26 (m, 6H), 1.10 (m, 1H, H-7), 1.03–0.92 (m, 7H, H-1; 0.99 (s, 3H, CH_3_-18*), 0.98 (s, 3H, CH_3_-19*)), 0.87 (d, 3H, *J =* 6.6, CH_3_-21). ^13^C NMR (CDCl_3_, 125 MHz): δ = 214.73 (s, C-12), 171.62 (s, C-24), 138.93 (s, C-1^Ph #^), 137.06 (s, C-1^ ^#^), 131.80 (d, C-3^, C-5^), 128.23 (d, C-3^Ph^, C-5^Ph^), 127.41 (d, C-2^Ph^, C-6^Ph^), 127.27 (d, C-4^Ph^), 121.21 (d, C-2^, C-6^), 116.47 (s, C-4^), 77.92 (d, C-3), 69.73 (t, C-25), 58.54 (d, C-14), 57.42 (s, C-13), 46.13 (d, C-17), 43.94 (d, C-9), 41.49 (d, C-5), 38.00 (t, C-11), 35.59 (d, C-8), 35.56 (s, C-10^§^), 35.40 (d, C-20), 35.14 (t, C-1^§^), 34.39 (t, C-23), 33.15 (t, C-4), 30.74 (t, C-22), 27.42, 27.10, 26.79, 25.93 (t, C-7), 24.22, 22.70 (q, C-19), 18.64 (q, C-21), 11.64 (q, C-18).

#### 3.2.10. *N*-(3″-(3′,5′-Di-*tert*-butyl-4′-hydroxyphenyl)propyl)-3α,12α-dibenzyloxy-5β-cholane-24-amide (**10**)

Compound **1** (0.30 g, 0.52 mmol) and CDI (0.10 g, 0.62 mmol) were dissolved in CH_2_Cl_2_ (10 mL) and stirred at room temperature for 2 h. Then, 4-(3-aminopropyl)-2,6-di-*tert*-butylphenol (0.17 g, 0.62 mmol) was added and the reaction mixture was stirred at 30–35 °C for 8 h. The reaction course was monitored by TLC (CHCl_3_). Reaction mixture was diluted with CH_2_Cl_2_ and Et_2_O, washed sequentially with H_2_O and brine, and dried over anhydrous MgSO_4_. Solvent was evaporated to dryness; crude product (0.50 g, quantitative yield) was purified by flash column chromatography (SiO_2_ + top layer Al_2_O_3_, CH_2_Cl_2_ with gradient 0–5% AcOEt) to give a pure sample of compound **10** (0.35 g, 82%) as a white amorphous solid. Mp 65.0 °C [decomposition]. [$\alpha_{D}^{25.7}$] +48 (*c* 0.10 g/100 mL; CHCl_3_). HRMS: Calc. for (C_55_H_79_O_4_N_1_)^+^ *m*/*z* = 817.6004; found *m*/*z* = 817.5991. ^1^H-NMR (CDCl_3_, 600 MHz): *δ* = 7.39–7.24 (m, 10H, aromatic protons of Ph and Ph′), 6.97 (s, 2H, H-2^, H-6^), 5.40 (m, NH), 5.07 (s, OH), 4.62 (d, 1H, *J =* 11.5, H-26), 4.53 (m, CH_2_-25), 4.28 (d, 1H, *J =* 11.5, H-26′), 3.68 (s, 1H, H-12), 3.36 (m, 1H, H-3), 3.28 (m, 2H, CH_2_-1″), 2.56 (m, 2H, CH_2_-3″), 2.18 (m, 1H, H-23), 2.07–1.95 (m, 2H, H-23′, H-17), 1.91–0.86 (m, 51H, 1.44 (s, 18H, CH_3_-9^, 10^, 11^, 12^, 13^, 14^), 0.93 (s, 3H, CH_3_-19), 0.89 (d, 3H, *J =* 6.5, CH_3_-21)), 0.70 (s, 3H, CH_3_-18). ^13^C NMR (CDCl_3_, 150 MHz): δ = 173.33 (s, C-24), 151.76 (s, C-4^), 139.16 (s, C-1^Ph^ *), 139.09 (s, C-1^Ph′^ *), 135.71 (d, C-3^, C-5^), 131.89 (s, C-1^), 128.10 (d, C-3^Ph^, C-5^Ph #^), 128.08 (d, C-3^Ph′^, C-5^Ph′ #^), 127.30 (d, C-2^Ph^, C-6^Ph^, C-2^Ph′^, C-6^Ph′^), 127.09 (d, C-4^Ph §^), 127.02 (d, C-4^Ph′ §^), 124.58 (d, C-2^, C-6^), 80.89 (d, C-12), 78.41 (d, C-3), 70.05 (t, C-26), 69.43 (t, C-25), 48.57 (d, C-14), 46.39 (s, C-13), 45.98 (d, C-17), 42.02 (d, C-5), 39.18 (t, C-1″), 35.92 (d, C-8), 35.21 (d, C-20), 35.16 (s, C-10^‡^), 34.37 (t, C-1^‡^), 34.11 (t, C-23), 33.62 (d, C-9), 33.33 (s, C-7^, C-8^ ^∞^), 33.15 (t, C-3″ ^∞^), 33.10 (t, C-4 ^∞^), 31.64 (t, C-22^†^), 31.54 (t, C-2″ ^†^), 30.17 (q, C-9^, 10^, 11^, 12^, 13^, 14^), 27.48 (t, C-11^¢^), 27.21 (t, C-16^¢^), 27.88 (t, C-2^¢^), 25.88 (t, C-7), 23.56 (t, C-15), 23.21 (q, C-19), 22.88 (t, C-6), 17.41 (q, C-21), 12.60 (q, C-18).

#### 3.2.11. *N*-(4′-Bromophenyl)-3α,12α-dibenzyloxy-5β-cholane-24-thioamide (**11**) 

Compound **7** (0.10 g, 0.14 mmol) and Lawesson’s reagent (0.17 g, 0.41 mmol) in dry toluene (5 mL) were heated at 90–100 °C for 3 h under an argon atmosphere. Then, the reaction mixture was evaporated to dryness and chromatographed (SiO_2_, CH_2_Cl_2_) to give a pure sample of compound **11** (0.075 g, 72%) as an orange amorphous substance. Mp 58.0 °C [decomposition]. [$\alpha_{D}^{24}$] +35 (*c* 0.20 g/100 mL; CHCl_3_). HRMS: Calc. for (C_44_H_56_O_2_N^79^BrS)^+^ *m*/*z* = 741.3210; found *m*/*z* = 741.3207. Elemental analysis calculated for C_44_H_56_O_2_NBrS: C, 71.14; H, 7.60; Br, 10.76; N, 1.89; O, 4.31; S, 4.32; found C, 69.63; H, 7.42; N 1.99; S, 4.42. ^1^H-NMR (CDCl_3_, 400 MHz): *δ* = 8.63 (s, NH), 7.56–7.42 (m, 4H, H-2^, H-3^, H-5^, H-6^), 7.41–7.20 (m, 10H, aromatic protons Ph and Ph′), 4.62 (d, 1H, *J =* 11.5, H-26), 4.52 (s, CH_2_-25), 4.27 (d, 1H, *J =* 11.5, H-26′), 3.68 (s, 1H, H-12), 3.36 (m, 1H, H-3), 2.78 (m, 1H, H-23), 2.63 (m, 1H, H-23′), 2.12–0.84 (m, 30H, 2.05 (m, H-17), 0.94 (d, 3H, *J =* 6.5, CH_3_-21), 0.92 (s, 3H, CH_3_-19)), 0.70 (s, 3H, CH_3_-18). ^13^C NMR (CDCl_3_, 75 MHz): δ = 206.24 (s, C-24), 139.16 (s, C-1^Ph^ *), 139.05 (s, C-1^Ph′^ *), 137.49 (s, C-1^), 131.76 (d, C-3^, C-5^), 128.14 (d, C-3^Ph^, C-5^Ph^, C-3^Ph′^, C-5^Ph′^), 127.37 (d, C-2^Ph^, C-6^Ph #^), 127.34 (d, C-2^Ph′^, C-6^Ph′ #^), 127.14 (d, C-4^Ph §^), 127.10 (d, C-4^Ph′ §^), 125.35 (d, C-2^, C-6^), 119.66 (s, C-4^), 80.88 (d, C-12), 78.45 (d, C-3), 70.07 (t, C-26), 69.48 (t, C-25), 48.66 (d, C-14), 46.47 (s, C-13), 45.94 (d, C-17), 45.47 (t, C-23), 42.02 (d, C-5), 35.93 (d, C-8), 35.70 (t, C-22), 35.16 (s, C-10; d, C-20), 34.38 (t, C-1), 33.66 (d, C-9), 33.12 (t, C-4), 27.59, 27.21, 27.12, 25.89 (t, C-7), 23.56 (t, C-15), 23.22 (q, C-19), 22.90 (t, C-6), 17.64 (q, C-21), 12.64 (q, C-18). Rotation around amide bond resulted in doubling of some signals in NMR ^1^H (NH, CH_2_-26, H-12, CH_2_-23). Rotamers ratio was about 85:15.

#### 3.2.12. *N′*-(3α,12α-Dibenzyloxy-5β-cholane-24-oyl)-2-(4″-bromophenyl)acetimidamide (**12**) 

Compound **1** (0.50 g, 0.87 mmol) and CDI (0.20 g, 1.21 mmol) were dissolved in dry CH_2_Cl_2_ (10 mL) and stirred at room temperature for 3 h. Then, 2-(4-bromophenyl)-*N′*-hydroxyacetimidamide (0.24 g, 1.21 mmol) was added and mixture was stirred at room temperature for 20 h rt, then refluxed for 3 h. The reaction course was monitored by TLC (CHCl_3_–MeOH, 60:1). The reaction mixture was evaporated to dryness and chromatographed (SiO_2_, CHC_3_) to give a pure sample of compound **12** (0.53 g, 77%) as a white amorphous solid. Mp 80.2–83.9 °C. [$\alpha_{D}^{26.7}$] +38 (*c* 0.20 g/100 mL; CHCl_3_). HRMS: *m*/*z* calcd. for (C_46_H_59_O_4_N_2_^79^Br)^+^ 782.3653; found 764.3528; *m*/*z* calcd. for (C_46_H_57_O_3_N_2_^79^Br)^+^ 764.3547 [M–H_2_O]^+^. ^1^H-NMR (CDCl_3_, 500 MHz): *δ* = 7.45–7.39 (m, 2H, H-3″, H-5″), 7.38–7.19 (m, 10H, aromatic protons of Ph and Ph′), 7.18–7.11 (m, 2H, H-2″, H-6″), 4.70 (br.s., NH_2_), 4.59 (d, 1H, *J =* 11.5, H-26), 4.51 (m, CH_2_-25), 4.27 (d, 1H, *J =* 11.5, H-26′), 3.66 (s, 1H, H-12), 3.46 (s, 2H, CH_2_-7″), 3.35 (m, 1H, H-3), 2.42 (m, 1H, H-23), 2.27 (m, 1H, H-23′), 2.03 (m, 1H, H-17), 1.91–0.83 (m, 29H, 0.91 (s, 3H, CH_3_-19), 0.90 (d, 3H, *J =* 6.0, CH_3_-21)), 0.69 (s, 3H, CH_3_-18). ^13^C NMR (CDCl_3_, 125 MHz): δ = 171.94 (s, C-24(5′)), 156.04 (s, C-3′), 139.03 (s, C-1^Ph^, C-1^Ph′^), 133.90 (s, C-1″), 131.75 (d, C-3″, C-5″), 130.34 (d, C-2″, C-6″), 128.02 (d, C-3^Ph^, C-5^Ph^, C-3^Ph′^, C-5^Ph′^), 127.28 (d, C-2^Ph^, C-6^Ph #^), 127.21 (d, C-2^Ph′^, C-6^Ph′ #^), 127.01 (d, C-4^Ph §^), 126.97 (d, C-4^Ph′ §^), 121.20 (s, C-4″), 80.76 (d, C-12), 78.36 (d, C-3), 70.00 (t, C-26), 69.36 (t, C-25), 48.53 (d, C-14), 46.35 (s, C-13), 45.86 (d, C-17), 41.95 (d, C-5), 36.61 (t, C-7″), 35.85 (d, C-8), 35.09 (s, C-10^‡^), 34.98 (d, C-20), 34.28 (t, C-1^‡^), 33.57 (d, C-9), 33.05 (t, C-4), 30.80 (t, C-23^†^), 29.59 (t, C-22^†^), 27.33, 27.13, 27.04, 25.82 (t, C-7), 23.49 (t, C-15), 23.13 (q, C-19), 22.82 (t, C-6), 17.30 (q, C-21), 12.54 (q, C-18).

#### 3.2.13. 5′-(24-Nor-3α,12α-dibenzyloxy-5β-cholan-23-yl)-3′-(methyl(4″-bromophenyl))-1′,2′,4′-oxadiazole (**13**) 

Compound **12** (0.37 g, 0.47 mmol) and *n*-Bu_4_NF (1 M in THF; 0.5 mL, 0.47 mmol) in THF (8 mL) were refluxed for 3 h, then the reaction mixture was evaporated to dryness, redissolved in CH_2_Cl_2_–Et_2_O, washed with brine, and dried over anhydrous MgSO_4_. Solvent was evaporated under vacuum. Crude product (0.37 g, quantitative yield) was purified by flash column chromatography (SiO_2_, CHCl_3_) to give a pure sample of **13** (0.21 g, 58%) as a colorless amorphous mass. [$\alpha_{D}^{24}$] +38 (*c* 0.10 g/100 mL; CHCl_3_). HRMS: Calc. for (C_46_H_57_O_3_N_2_^79^Br)^+^ *m*/*z* = 764.3547; found *m*/*z* = 673.3014; calc. for (C_39_H_50_O_3_N_2_^79^Br)^+^ *m*/*z* = 673.3000 [M–PhCH_2_]^+^. ^1^H-NMR (CDCl_3_, 500 MHz): *δ* = 7.45–7.39 (m, 2H, H-3″, H-5″), 7.37–7.20 (m, 10H, aromatic protons of Ph and Ph′), 7.20–7.15 (m, 2H, H-2″, H-6″), 4.59 (d, 1H, *J =* 11.4, H-26), 4.52 (m, CH_2_-25), 4.26 (d, 1H, *J =* 11.4, H-26′), 3.97 (s, 2H, CH_2_-7″), 3.65 (s, 1H, H-12), 3.35 (m, 1H, H-3), 2.85 (m, 1H, H-23), 2.70 (m, 1H, H-23′), 2.04 (m, 1H, H-17), 1.94–0.81 (m, 29H, 0.91 (s, 3H, CH_3_-19), 0.90 (d, 3H, *J =* 6.6, CH_3_-21)), 0.65 (s, 3H, CH_3_-18). ^13^C NMR (CDCl_3_, 125 MHz): δ = 180.68 (s, C-24 (5′)), 168.72 (s, C-3′), 139.21 (s, C-1^Ph^ *), 139.10 (s, C-1^Ph′^ *), 134.49 (s, C-1″), 131.66 (d, C-3″, C-5″), 130.59 (d, C-2″, C-6″), 128.16 (d, C-3^Ph^, C-5^Ph^, C-3^Ph′^, C-5^Ph′^), 127.41 (d, C-2^Ph^, C-6^Ph #^), 127.35 (d, C-2^Ph′^, C-6^Ph′ #^), 127.14 (d, C-4^Ph^, C-4^Ph′^), 120.99 (s, C-4″), 80.79 (d, C-12), 78.48 (d, C-3), 70.12 (t, C-26), 69.51 (t, C-25), 48.62 (d, C-14), 46.52 (s, C-13), 45.96 (d, C-17), 42.12 (d, C-5), 36.01 (d, C-8), 35.25 (s, C-10^‡^), 35.14 (d, C-20), 34.44 (t, C-1^‡^), 33.69 (d, C-9), 33.19 (t, C-4), 32.58 (t, C-22^†^), 31.67 (t, C-7″ ^†^), 27.45, 27.28, 27.19, 25.93 (t, C-7), 23.59 (t, C-15^§^), 23.37 (t, C-23^§^), 23.26 (q, C-19), 22.95, 17.29 (q, C-21), 12.60 (q, C-18).

#### 3.2.14. *N′*-(3α,12α-Dibenzyloxy-5β-cholane-24-oyl)-4″-bromobenzimidamide (**14**)

Compound **1** (0.50 g, 0.87 mmol) and CDI (0.17 g, 1.2 mmol) were dissolved in dry CH_2_Cl_2_ (10 mL) and stirred at room temperature for 3 h. Then, 4-bromo*-N′*-hydroxybenzimidamide (0.23 g, 1.2 mmol) was added and the mixture was stirred at room temperature overnight. The reaction course was monitored by TLC (CHCl_3_). The reaction mixture was evaporated to dryness and chromatographed (SiO_2_, CHC_3_) to give a pure sample of compound **14** (0.54 g, 80%) as a white amorphous solid. Mp 144.5–147.4°C. [$\alpha_{D}^{24}$] +30 (*c* 0.10 g/100 mL; CHCl_3_). HRMS: Calc. for (C_45_H_57_O_4_N_2_^79^Br)^+^ *m*/*z* = 768.3496; found *m*/*z* = 659.2835; calc. for (C_38_H_48_O_3_N_2_^79^Br)^+^ *m*/*z* = 659.2843 [M–PhCH_2_–H_2_O]^+^. ^1^H-NMR (CDCl_3_, 500 MHz): *δ* = 7.57–7.48 (m, 2H, H-2″, H-3″, H-5″, H-6″), 7.39–7.21 (m, 10H, aromatic protons of Ph and Ph′), 5.06 (br.s., NH_2_), 4.60 (d, 1H, *J =* 11.5, H-26), 4.51 (m, CH_2_-25), 4.27 (d, 1H, *J =* 11.5, H-26′), 3.67 (s, 1H, H-12), 3.34 (m, 1H, H-3), 2.49 (m, 1H, H-23), 2.34 (m, 1H, H-23′), 2.03 (m, 1H, H-17), 1.92–0.83 (m, 29H, 0.91 (s, 3H, CH_3_-19), 0.90 (d, 3H, CH_3_-21)), 0.69 (s, 3H, CH_3_-18). ^13^C NMR (CDCl_3_, 125 MHz): δ = 171.51 (s, C-24 (5′)), 155.08 (s, C-3′), 139.13 (s, C-1^Ph^ *), 139.03 (s, C-1^Ph′^ *), 131.73 (d, C-3″, C-5″), 129.92 (s, C-1″), 128.14 (d, C-2″, C-6″ ^∞^), 128.12 (d, C-3^Ph^, C-5^Ph^^∞^), 128.08 (d, C-3^Ph′^, C-5^Ph′^^∞^), 127.35 (d, C-2^Ph^, C-6^Ph #^), 127.33 (d, C-2^Ph′^, C-6^Ph′ #^), 127.14 (d, C-4^Ph §^), 127.06 (d, C-4^Ph′ §^), 125.21 (s, C-4″), 80.84 (d, C-12), 78.39 (d, C-3), 70.02 (t, C-26), 69.45 (t, C-25), 48.58 (d, C-14), 46.41 (s, C-13), 45.93 (d, C-17), 41.99 (d, C-5), 35.90 (d, C-8), 35.13 (s, C-10^‡^), 35.07 (d, C-20), 34.37 (t, C-1^‡^), 33.60 (d, C-9), 33.07 (t, C-4), 30.89 (t, C-23^†^), 29.68 (t, C-22^†^), 27.44, 27.20, 27.09, 25.88 (t, C-7), 23.57 (t, C-15), 23.23 (q, C-19), 22.84 (t, C-6), 17.34 (q, C-21), 12.64 (q, C-18).

#### 3.2.15. 5′-(24-Nor-3α,12α-Dibenzyloxy-5β-cholan-23-yl)-3′-(4″-bromophenyl)-1′,2′,4′-oxadiazole (**15**) 

Compound **14** (0.40 g, 0.52 mmol) and *n*-Bu_4_NF (1 M in THF; 0.25 mL, 0.25 mmol) in THF (5 mL) were refluxed for 1 h, then the reaction mixture was evaporated to dryness, dissolved in CH_2_Cl_2_–Et_2_O, washed with brine, and dried over anhydrous MgSO_4_. Solvent was evaporated under vacuum. Crude product (0.40 g, quantitative yield) was purified by flash column chromatography (SiO_2_, CH_2_Cl_2_) to give a pure sample of **15** (0.34 g, 89%) as a colorless amorphous solid. Mp 130.2–135.5 °C. [$\alpha_{D}^{24}$] +20 (*c* 0.10 g/100 mL; CHCl_3_). HRMS: Calc. for (C_45_H_55_O_3_N_2_^79^Br)^+^ *m*/*z* = 750.3391; found *m*/*z* = 659.2839; calc. for (C_38_H_48_O_3_N_2_^79^Br)^+^ *m*/*z* = 659.2843 [M–PhCH_2_]^+^. ^1^H-NMR (CDCl_3_, 400 MHz): *δ* = 7.93 (m, 2H, H-2″, H-6″), 7.60 (m, 2H, H-3″, H-5″), 7.40–7.20 (m, 10H, aromatic protons of Ph and Ph′), 4.61 (d, 1H, *J =* 11.5, H-26), 4.52 (m, CH_2_-25), 4.27 (d, 1H, *J =* 11.5, H-26′), 3.67 (s, 1H, H-12), 3.34 (m, 1H, H-3), 2.96 (m, 1H, H-23), 2.79 (m, 1H, H-23′), 2.08 (m, 1H, H-17), 2.03–0.86 (m, 29H, 0.94 (d, 3H, *J =* 6.5, CH_3_-21), 0.91 (s, 3H, CH_3_-19)), 0.69 (s, 3H, CH_3_-18). ^13^C NMR (CDCl_3_, 100 MHz): δ = 180.71 (s, C-24(5′)), 167.38 (s, C-3′), 139.10 (s, C-1^Ph^ *), 139.08 (s, C-1^Ph′^ *), 131.96 (d, C-3″, C-5″), 128.77 (d, C-2″, C-6″), 128.17 (d, C-3^Ph^, C-5^Ph^, C-3^Ph′^, C-5^Ph′^), 127.37 (d, C-2^Ph^, C-6^Ph^, C-2^Ph′^, C-6^Ph′^), 127.15 (d, C-4^Ph^, C-4^Ph′^), 125.82 (s, C-1″ ^#^), 125.43 (s, C-4″ ^#^), 80.79 (d, C-12), 78.42 (d, C-3), 70.07 (t, C-26), 69.49 (t, C-25), 48.60 (d, C-14), 46.49 (s, C-13), 45.92 (d, C-17), 42.04 (d, C-5), 35.96 (d, C-8), 35.23 (d, C-20), 35.19 (s, C-10^‡^), 34.41(t, C-1^‡^), 33.63 (d, C-9), 33.12 (t, C-4), 32.64 (t, C-22), 27.50, 27.24, 27.14, 25.90 (t, C-7), 23.59 (t, C-15^§^), 23.35 (t, C-23^§^), 23.25 (q, C-19), 22.89, 17.28 (q, C-21), 12.65 (q, C-18).

#### 3.2.16. Methyl 3α,12α-Dibenzyloxy-5β-cholan-24-oate (**16**)

Compound **1** (1.0 g, 1.75 mmol) and dimethylsulfate (0.45 mL, 4.81 mmol) were dissolved in acetone (50 mL); K_2_CO_3_ (0.89 g, 6.47 mmol) and KI (0.02 g, 0.12 mmol) were added to the solution. Reaction mixture was stirred overnight at room temperature. The reaction course was monitored by TLC (CHCl_3_). Then, acetone was evaporated under the vacuum. Reaction mixture was dissolved in CHCl_3_, washed with water and brine, and dried over anhydrous MgSO_4_. Solvent was evaporated under vacuum. Crude product (1.23 g) was purified by flash column chromatography (SiO_2_, CHCl_3_) to give pure **11** (1.1 g, quantitative yield) as a white amorphous solid. Mp 93.3–93.7 °C. [$\alpha_{D}^{21}$] +50 (*c* 0.20 g/100 mL; CHCl_3_). HRMS: Calc. for (C_39_H_54_O_4_)^+^ *m*/*z* = 586.4017; found *m*/*z* = 586.4022. ^1^H NMR (CDCl_3_, 500 MHz): *δ* = 7.40–7.22 (m, 10H, aromatic protons), 4.61 (d, 1H, *J =* 11.4, H-26), 4.53 (m, 2H, CH_2_-25), 4.29 (d, 1H, *J =* 11.4, H-26′), 3.68 (s, 1H, H-12), 3.66 (s, 3H, CH_3_-27), 3.36 (m, 1H, H-3), 2.37 (m, 1H, H-23), 2.21 (m, 1H, H-23′), 2.04 (m, 1H, H-17), 1.92–1.67 (m, 9H), 1.66–1.52 (m, 2H), 1.49–0.85 (m, 19H, 0.93 (s, 3H, CH_3_-19), 0.89 (d, 3H, *J_21,20_* = 6.1, CH_3_-21)), 0.71 (s, 3H, CH_3_-18). ^13^C NMR (CDCl_3_, 125 MHz): δ = 174.61 (s, C-24), 139.06 (s, C-1^Ph^, C-1^Ph′^), 128.08 (d, C-3^Ph^, C-5^Ph^ *), 128.07 (d, C-3^Ph′^, C-5^Ph′^ *), 127.30 (d, C-2^Ph^, C-6^Ph ∞^), 127.28 (d, C-2^Ph′^, C-6^Ph′ ∞^), 127.07 (d, C-4^Ph #^), 127.02 (d, C-4^Ph′ #^), 80.79 (d, C-12), 78.36 (d, C-3), 70.03 (t, C-26), 69.39 (t, C-25), 51.26 (q, C-27), 48.51 (d, C-14), 46.37 (s, C-13), 45.97 (d, C-17), 41.98 (d, C-5), 35.89 (d, C-8), 35.13 (s, C-10^§^), 35.10 (d, C-20), 34.33 (t, C-1^§^), 33.56 (d, C-9), 33.04 (t, C-4), 30.71 (t, C-23^‡^), 30.75 (t, C-22^‡^), 27.40, (t, C-11^†^), 27.19 (t, C-16^†^), 27.07 (t, C-2^†^), 25.87 (t, C-7), 23.55 (t, C-15), 23.19 (q, C-19), 22.85 (t, C-6), 17.26 (q, C-21), 12.57 (q, C-18).

#### 3.2.17. 3α,12α-Dibenzyloxy-5β-cholan-24-hydrazide (**17**) 

Compound **16** (0.9 g, 1.54 mmol) and hydrazine hydrate (2.5 mL, 51.5 mmol) were dissolved in EtOH (25 mL), and solution was refluxed until full conversion was reached. The reaction course was monitored by TLC (CHCl_3_–MeOH; 25:0.5). Reaction mixture was concentrated under vacuum and poured into cold water. Precipitate was filtered, washed with water, and dried under vacuum. Crude product was purified by flash column chromatography (SiO_2_, CHCl_3_ with 0–2% MeOH) to give product **16** (0.66 g, 73%) and recrystallized from EtOH to give a pure sample of **17** as a white amorphous solid. Mp 179.0–180.0 °C (EtOH). [$\alpha_{D}^{21}$] +61 (*c* 0.20 g/100 mL; CHCl_3_). HRMS: *m*/*z* calcd. for (C_38_H_54_O_3_N_2_)^+^ 586.4129; found 586.4131. ^1^H NMR (CDCl_3_, 400 MHz): *δ* = 7.41–7.18 (m, 10H, aromatic protons), 5.24 (br.s., NH_2_), 4.58 (d, 1H, *J =* 11.4, H-26), 4.51 (m, 2H, CH_2_-25), 4.25 (d, 1H, *J =* 11.4, H-26′), 3.64 (s, 1H, H-12), 3.34 (m, 1H, H-3), 2.21 (m, 1H, H-23), 2.10–1.92 (m, 2H, H-23′, H-17), 1.92–0.80 (m, 29H, 0.90 (s, 3H, CH_3_-19), 0.86 (d, 3H, *J_21,20_* = 5.8, CH_3_-21)), 0.67 (s, 3H, CH_3_-18). ^13^C NMR (CDCl_3_, 100 MHz): δ = 174.25 (s, C-24), 139.07 (s, C-1^Ph^, C-1^Ph′^), 128.10 (d, C-3^Ph^, C-5^Ph^, C-3^Ph′^, C-5^Ph′^), 127.33 (d, C-2^Ph^, C-6^Ph^ *), 127.30 (d, C-2^Ph′^, C-6^Ph′^ *), 127.09 (d, C-4^Ph^, C-4^Ph′^), 80.83 (d, C-12), 78.37 (d, C-3), 70.07 (t, C-26), 69.38 (t, C-25), 48.52 (d, C-14), 46.38 (s, C-13), 46.03 (d, C-17), 41.99 (d, C-5), 35.89 (d, C-8), 35.20 (d, C-20), 35.13 (s, C-10^§^), 34.35 (t, C-1^§^), 33.58 (d, C-9), 33.06 (t, C-4), 31.22 (t, C-23^‡^), 30.98 (t, C-22^‡^), 27.47, (t, C-11^†^), 27.20 (t, C-16^†^), 27.08 (t, C-2^†^), 25.87 (t, C-7), 23.57 (t, C-15), 23.21 (q, C-19), 22.87 (t, C-6), 17.36 (q, C-21), 12.62 (q, C-18).

#### 3.2.18. *N’*-(4″-Bromobenzylidene)-3α,12α-dibenzyloxy-5β-cholan-24-hydrazide (**18**) 

Compound **17** (0.54 g, 0.93 mmol) and *p*-bromobenzaldehyde (0.17 g, 0.93 mmol) were dissolved in EtOH (10 mL), and the reaction mixture was refluxed for 3 h. The reaction course was monitored by TLC (CHCl_3_–MeOH; 30:0.5). Reaction mixture was evaporated to dryness and crude product was purified by flash column chromatography (SiO_2_, CHCl_3_) to give product **18** (0.65 g, 93%) as a white amorphous solid. Mp 147.7–150.0 °C (AcOEt). [$\alpha_{D}^{26.5}$] +48 (*c* 0.10 g/100 mL; CHCl_3_). HRMS: Calc. for (C_45_H_57_O_3_N_2_^79^Br)^+^ *m*/*z* = 752.3547; found *m*/*z* = 661.2988; calc. for (C_38_H_50_O_3_N_2_^79^Br)^+^ *m*/*z* [M–PhCH_2_]^+^ = 661.2999. ^1^H-NMR (CDCl_3_, 500 MHz): *δ* = 10.42 (s, NH^&^), 7.78 (s, H-5′ ^&^), 7.56–7.45 (m, 4H, H-2″, H-3″, H-5″, H-6″), 7.41–7.18 (m, 10H, aromatic protons of Ph and Ph′), 4.62 (d, 1H, *J =* 11.4, H-26), 4.53 (m, CH_2_-25), 4.32 (d, 1H, *J =* 11.4, H-26′), 3.70 (s, 1H, H-12), 3.36 (m, 1H, H-3), 2.80 (m, 1H, H-23), 2.63 (m, 1H, H-23′), 2.11 (m, 1H, H-17), 1.96–0.88 (m, 29H, 0.99 (d, 3H, *J =* 6.0, CH_3_-21), 0.93 (s, 3H, CH_3_-19)), 0.72 (s, 3H, CH_3_-18). ^13^C NMR (CDCl_3_, 125 MHz): δ = 177.49 (s, C-24 (2′)), 142.28 (s, C-5′), 139.19 (s, C-1^Ph^ *), 139.16 (s, C-1^Ph′^ *), 132.90 (s, C-1″), 131.78 (d, C-3″, C-5″), 128.31 (d, C-2″, C-6″), 128.12 (d, C-3^Ph^, C-5^Ph^, C-3^Ph′^, C-5^Ph′^), 127.33 (d, C-2^Ph^, C-6^Ph #^), 127.32 (d, C-2^Ph′^, C-6^Ph′ #^), 127.10 (d, C-4^Ph ∞^), 127.06 (d, C-4^Ph′ ∞^), 123.92 (s, C-4″), 80.90 (d, C-12), 78.48 (d, C-3), 70.15 (t, C-26), 69.46 (t, C-25), 48.62 (d, C-14), 46.50 (s, C-13), 46.15 (d, C-17), 42.09 (d, C-5), 36.00 (d, C-8), 35.52 (d, C-20), 35.23 (s, C-10^‡^), 34.42 (t, C-1^‡^), 33.69 (d, C-9), 33.16 (t, C-4), 31.03 (t, C-23^§^), 29.68 (t, C-22^§^), 27.54 (t, C-11^‡^), 27.27 (t, C-16^‡^), 27.17 (t, C-2^‡^), 25.93 (t, C-7), 23.61 (t, C-15), 23.24 (q, C-19), 22.97 (t, C-6), 17.61 (q, C-21), 12.67 (q, C-18).

#### 3.2.19. 2′-(24-Nor-3α,12α-dibenzyloxy-5β-cholan-23-yl)-5′-(4″-bromophenyl)-1′,3′,4′-oxadiazole (**19**)

Compound **18** (0.57 g, 0.75 mmol) was dissolved in DMSO (10 mL) then I_2_ (0.29 g, 1.13 mmol) and potassium carbonate (0.39 g, 2.8 mmol) were added to the solution. Reaction mixture was heated at 70 °C for 3 h, then cooled to room temperature, diluted with Na_2_S_2_O_3_ (aq.), and stirred for 30 min. Reaction mixture was extracted with AcOEt–Et_2_O, washed with brine, and dried over anhydrous MgSO_4_. Solvent was evaporated to give crude product **19** (0.52 g, 93%). Crude product was chromatographed (SiO_2_, CH_2_Cl_2_ with 0–100% gradient CHCl_3_) to give pure compound **19** (0.15g, 27%). Mp 131.4 °C [decomposition]. [$\alpha_{D}^{24}$] +36 (*c* 0.10 g/100 mL; CHCl_3_). HRMS: Calc. for (C_45_H_55_O_3_N_2_^79^Br)^+^ *m*/*z* = 750.3391; found *m*/*z* = 659.2838 and *m*/*z* = 750.3354; calc. for (C_38_H_48_O_3_N_2_^79^Br)^+^ *m*/*z* = 659.2843. ^1^H-NMR (CDCl_3_, 300 MHz): *δ* = 7.88 (m, 2H, H-2″, H-6″), 7.62 (m, 2H, H-3″, H-5″), 7.44–7.18 (m, 10H, aromatic protons of Ph and Ph′), 4.61 (d, 1H, *J =* 11.5, H-26), 4.51 (m, CH_2_-25), 4.28 (d, 1H, *J =* 11.5, H-26′), 3.68 (s, 1H, H-12), 3.34 (m, 1H, H-3), 2.93 (m, 1H, H-23), 2.78 (m, 1H, H-23′), 2.19–0.82 (m, 30H; 2.09 (m, 1H, H-17), 0.96 (d, 3H, *J =* 6.3, CH_3_-21), 0.91 (s, 3H, CH_3_-19)), 0.69 (s, 3H, CH_3_-18). ^13^C NMR (CDCl_3_, 100 MHz): δ = 167.60 (s, C-5′), 163.83 (s, C-24 (2′)), 139.12 (s, C-1^Ph^, C-1^Ph′^), 132.22 (d, C-3″, C-5″), 128.17 (d, C-3^Ph^, C-5^Ph^, C-3^Ph′^, C-5^Ph′^), 128.05 (d, C-2″, C-6″), 127.38 (d, C-2^Ph^, C-6^Ph^, C-2^Ph′^, C-6^Ph′^), 127.16 (d, C-4^Ph^, C-4^Ph′^), 125.98 (s, C-1″), 122.91 (s, C-4″), 80.81 (d, C-12), 78.43 (d, C-3), 70.08 (t, C-26), 69.50 (t, C-25), 48.63 (d, C-14), 46.50 (s, C-13), 45.98 (d, C-17), 42.06 (d, C-5), 35.97 (d, C-8), 35.23 (d, C-20), 35.20 (s, C-10^‡^), 34.42 (t, C-1^‡^), 33.65 (d, C-9), 33.13 (t, C-4), 32.58 (t, C-22), 27.55 (t, C-11*), 27.24 (t, C-16*), 27.15 (t, C-2*), 25.91 (t, C-7), 23.60 (t, C-15), 23.25 (q, C-19), 22.90 (t, C-6), 22.21 (t, C-23), 17.31 (q, C-21), 12.65 (q, C-18).

### 3.3. Detection of TDP1 Activity

TDP1 activity was detected, as described [29] by intensity measurement in a reaction of quencher removal from a fluorophore quencher–coupled DNA oligonucleotide catalyzed by TDP1. The reaction was carried out at different concentrations of inhibitors (the control samples contained 1% of DMSO). The reaction mixtures contained TDP1 buffer (50 mM Tris-HCl pH 8.0, 50 mM NaCl, and 7 mM β-mercaptoethanol), 50 nM biosensor, and an inhibitor being tested. Purified TDP1 (1.5 nM) triggered the reaction. The biosensor (5′-[FAM] AAC GTC AGGGTC TTC C [BHQ]-3′) was synthesized in the Laboratory of Nucleic Acid Chemistry at the Institute of Chemical Biology and Fundamental Medicine (Novosibirsk, Russia).

The reactions were incubated on a POLARstar OPTIMA fluorimeter (BMG LABTECH, GmbH, Ortenberg, Germany) to measure fluorescence every 60 s (ex. 485/em. 520 nm) during the linear phase (here, data from minute 0 to minute 8). The values of IC_50_ were determined using a six-point concentration response curve in a minimum of three independent experiments and were calculated using MARS Data Analysis 2.0 (BMG LABTECH, Ortenberg, Germany).

### 3.4. Gel-Based TDP2 Activity Assay

The oligonucleotide with tyrosine residue on the 5′-end (5′-tyrosine-AAC GTC AGG GTC TTC C-FAM-3′) was used for the TDP2 enzyme activity assay. The TDP2 reaction products were separated under denaturing conditions in polyacrylamide gel (PAGE). The reaction was performed in a final volume of 20 μL using 100 nM substrate incubated with 200 nM recombinant human TDP2 in the absence or presence of the inhibitor for 10 min at 37 °C in a buffer containing 50 mM Tris-HCl, pH 8.0, 50 mM NaCl, 7 mM β-mercaptoethanol. Reactions were terminated by the addition of gel loading buffer contained 10% formamide, 7 M carbamide, 0.1% xylene cyanol, and 0,1% bromophenol blue. The samples were heated at 90 °C for 7 min before loading on the gel. The reaction products were separated by electrophoresis in a 20% denaturing PAGE with 7 M carbamide. A Typhoon FLA 9500 phosphorimager (GE Healthcare) was used for gel scanning and imaging. Each experiment was carried out in three independent replicates. The data were analyzed with QuantityOne 4.6.7 software. 

### 3.5. Cytotoxicity Assays

Cytotoxicity of the compounds to HeLa (human cervical cancer) and HEK293A (human embryonic kidney) cell lines was examined using the EZ4U Cell Proliferation and Cytotoxicity Assay (Biomedica, Vienna, Austria), according to the manufacturer’s protocols. The cells were grown in Iscove’s modified Dulbecco’s medium (IMDM) with 40 μg/mL gentamicin, 50 IU/mL penicillin, 50 μg/mL streptomycin (MP Biomedicals, Waltham, MA, USA), and 10% of fetal bovine serum (Biolot, Saint Petersburg, Russia) in a 5% CO_2_ atmosphere. After the formation of a 30–50%monolayer, the tested compounds were added to the medium. TDP1 inhibitors were dissolved in DMSO and topotecan in water. The volume of the added reagents was 1/100 of the total volume of the culture medium, and the amount of DMSO was 1% of the final volume. Control cells were grown in the presence of 1% DMSO. The cell culture was monitored for three days.

### 3.6. Modelling and Screening

The compounds were docked against the crystal structure of TDP1 (PDB ID: 6W7K, resolution 1.70 Å) [35] and TDP2 (PDB ID: 5J3S, resolution 3.40 Å) [36], which were obtained from the Protein Data Bank (PDB) [17,18]. The GOLD (v2020.2.0) software suite was used to prepare the crystal structures for docking, in other words, the hydrogen atoms were added, water molecules deleted, and the co-crystallized ligands identified: TDP1 4-[(2-phenylimidazo[1,2-*a*]pyridin-3-yl)amino]benzene-1,2-dicarboxylic acid (TG7) and TDP2 2,4-dioxo-10-[3-(1*H*-tetrazol-5-yl)phenyl]-2,3,4,10-tetrahydropyrimido[4,5-*b*]quinoline-8-carbonitrile (6FQ). The Scigress version FJ 2.6 program [39] was used to build the inhibitors and the MM3 [40,41,42] force field was applied to identify the global minimum using the CONFLEX method [43], followed by structural optimization. The docking center for the TDP1 catalytic pocket was defined as the position of the co-crystallized ligand TG7 as well as for TDP2 (6FQ) with a 10 Å radius. Fifty docking runs were allowed for each ligand with default search efficiency (100%). The basic amino acids lysine and arginine were defined as protonated. Furthermore, aspartic and glutamic acids were assumed to be deprotonated. The GoldScore (GS) [44], ChemScore (CS) [45,46], ChemPLP (Piecewise Linear Potential) [47], and ASP (Astex Statistical Potential) [48] scoring functions were implemented to predict the binding modes and relative binding energies of the ligands using the GOLD v2020.2.0 software suite.

The QikProp 6.2 [49] software package was used to calculate the molecular descriptors. The reliability of QikProp was established for the calculated descriptors [50]. The Known Drug Indexes (KDI) were calculated from the molecular descriptors as described by Eurtivong and Reynisson [38]. For application in Excel, columns for each property were created and the following equations used to derive the KDI numbers for each descriptor: KDI MW: =EXP(−((*MW* − 371.76)^2)/(2 × (112.76^2))), KDI Log P: =EXP(−((*LogP* − 2.82)^2)/(2 × (2.21^2))), KDI HD: =EXP(−((*HD* − 1.88)^2)/(2 × (1.7^2))), KDI HA: =EXP(−((*HA* − 5.72)^2)/(2 × (2.86^2))), KDI RB =EXP(−((*RB* − 4.44)^2)/(2 × (3.55^2))), and KDI PSA: =EXP(−((*PSA* − 79.4)^2)/(2 × (54.16^2))). These equations can simply be copied into Excel and the descriptor name (e.g., *MW*) substituted with the value in the relevant column. To derive KDI_2A_, this equation was used: = (KDI MW + KDI LogP + KDI HD + KDI HA + KDI RB + KDI PSA) and for KDI_2B_: = (KDI MW × KDI LogP × KDI HD × KDI HA × KDI RB × KDI PSA).

## 4. Conclusions

Deoxycholic acid amides (*para*-bromoanilides, tryptamides, and amide containing 2,6-bis*-tert-*butylphenol moiety) with benzyl ether groups on the steroid scaffold as well as 1,2,4- and 1,3,4-oxadiazole derivatives were synthesized. The compounds were tested against the TDP1 and TDP2 DNA repair enzymes. Good inhibitory activity against TDP1 with IC_50_ in the submicromolar range was obtained. Additionally, it was shown that the amides as well as a 1,3,4-oxadiazole derivatives moderately inhibited the TDP2 enzyme. The tryptamide derivative **5** and the *para*-bromanilide **8**, with the benzyloxy substituent at the C-3 position and free hydroxy group on C-12, are inhibitors of both TDP1 and TDP2 and enhance the cytotoxicity of topotecan. Comparison of the benzyloxy derivatives of deoxycholic acid with the previously synthesized *bis*-acetoxy [11] and *bis*-methoxy derivatives [12] by molecular modeling showed that the addition of the benzyloxy moieties prevents binding to the allosteric site. According to the modeling, the ligands are anchored into the catalytic pocket of TDP1 by one hydrogen bond to the backbone of Gly458 as well as π–π stacking and between the indolyl rings, in the case of ligand **5**, and Tyr590. It can therefore be concluded that these derivatives form a good starting point for further development of TDP1 and TDP2 inhibitors to be used in combination with TOP1 poisons.

## Figures and Tables

**Table 1:** TDP1 and TDP2 inhibitory activity of compounds **1**–**18**.

	Compound	TDP1 IC_50_^1^, μM	TDP2 (Inhibitor Concentration 1 mM) ^2^,%	HeLa CC_50_^3^, μM	HEK293A CC_50_^3^, μM
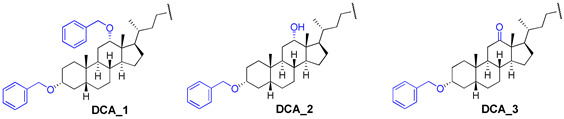					
**4**	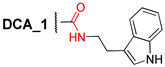	0.75 ± 0.17	35 ± 4	>100	>100
**5**	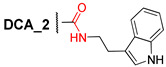	0.24 ± 0.01	28 ± 5	>100	>100
**6**	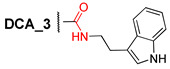	0.59 ± 0.13	45 ± 4	>100	>100
**7**	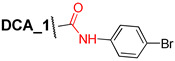	0.97 ± 0.46	44 ± 5	ND	ND
**8**	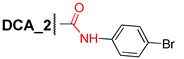	0.57 ± 0.30	47 ± 3	>100	>100
**9**	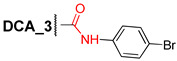	0.71 ± 0.34	39 ± 6	>100	>100
**10**	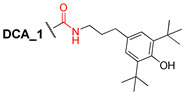	0.37 ± 0.17	45 ± 6	>100	>100
**12**	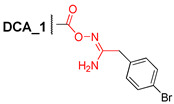	0.32 ± 0.13	40 ± 6	ND	ND
**13**	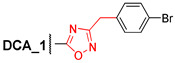	0.53 ± 0.23	>50	ND	ND
**14**	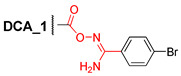	0.33 ± 0.15	45 ± 5	ND	ND
**15**	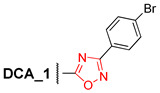	0.42 ± 0.30	>50	ND	ND
**17**	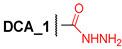	0.23 ± 0.08	>50	ND	ND
**18**	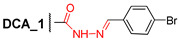	0.51 ± 0.21	48 ± 7	ND	ND
**19**	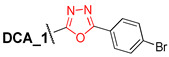	1.01 ± 0.43	50 ± 10	ND	ND
**Fur^4^**		1.2 ± 0.3	ND	ND	ND
**Topotecan**		ND	ND	0.9 ± 0.1	0.33 ± 0.07
**Etoposide**		ND	ND	6.2 ± 0.6	5.9 ± 1.8

^1^**IC_50_**—concentration that inhibits the activity of an enzyme by 50%. ^2^ The residual activity of TDP2 (%) in the presence of compounds **4–10, 12–15, 16–19**. ^3^ **CC_50_**—average cytotoxic concentration causing 50% cell death after 72 h of treatment measured by the colorimetric EZ4U assay. ^4^ Fur—furamidine, commercial TDP1 inhibitor [30]. ND—not determined.

## Data Availability

The data presented in this study are available on request from the corresponding authors.

## Supplementary Materials

The following are available online at, NMR ^1^H and ^13^C, HRMS of benzyloxy-DCA derivatives (1–19); Table S1. The influence of the deoxycholic acid derivative **8** at 10 μM on topotecan cytotoxicity; Table S2. The influence of the deoxycholic acid derivative **5** at 10 μM on topotecan cytotoxicity; Figure S1. The compounds **4**–**19** inhibit TDP2 in 1 mM concentration; Figure S2. The influence of derivatives **4**–**6** and **8**–**10** (5 μM) on etoposide cytotoxicity against HeLa cells; **Molecular modeling section.** Further discussion and details are given for the modeling results; Table S3. The binding affinities as predicted by the scoring functions used to the TDP2 binding site as well as the RMSD values for the co-crystallized ligand (6FQ); Table S4. The binding affinities as predicted by the scoring functions used for the catalytic TDP1 binding pocket; Table S5. The molecular descriptors and their corresponding Known Drug Indexes 2a and 2b (KDI_2a/2b_). The R^2^ numbers derived do not contain derivatives **13** and **DCA** since they are outliers; Table S6. Definition of lead-like, drug-like, and Known Drug Space (KDS) in terms of molecular descriptors. The values given are the maxima for each descriptor for the volumes of chemical space used; Table S7. **Structures of DCA derivatives.** Effect of benzyloxy vs. acetoxy groups in the steroid scaffold on TDP1; Table S8. **Structures of DCA derivatives.** Effect of benzyloxy vs. methoxy groups in the steroid scaffold on TDP1.

## Sample Availability

Samples of the compounds are not available from the authors.
