# Supplementary material for: New Deoxycholic Acid Derived Tyrosyl-DNA Phosphodiesterase 1 Inhibitors Also Inhibit Tyrosyl-DNA Phosphodiesterase 2
Source: Molecules. 2021 Dec 23;27(1):72. doi: 10.3390/molecules27010072 (PMC8746696; doi:10.3390/molecules27010072)
Supplement: Supplementary file 1 [file molecules-27-00072-s001.zip › molecules-1492190-supplementary.pdf]

Benzyloxy derivatives of deoxycholic acid as new type of dual tyrosyl-DNA phosphodiesterase 1 and 2 inhibitors

Oksana V. Salomatina[a], Nadezhda. S. Dyrkheeva[b], Irina I. Popadyuk[a], Alexandra L. Zakharenko[b], Ekaterina S. Ilina[b], Nina I. Komarova[a], Jóhannes Reynisson[c], Nariman F. Salakhutdinov[a], Olga I. Lavrik[b], Konstantin P. Volcho[a]*

[a] N.N. Vorozhtsov Novosibirsk Institute of Organic Chemistry, SB RAS, 9, Lavrent'ev Ave., Novosibirsk 630090, Russian Federation

[b] Institute of Chemical Biology and Fundamental Medicine, SB RAS, 8, Lavrent'ev Ave., Novosibirsk 630090, Russian Federation

[c] School of Pharmacy and Bioengineering, Keele University, Hornbeam Building, Staffordshire ST5 5BG, UK

* Corresponding author: volcho@nioch.nsc.ru; Tel.: +7 383 3308870

NMR ^{1}H and **^{13}C, HRMS** of benzyloxy-DCA derivatives (**1-19**)

Table S1. The influence of the deoxycholic acid derivative **8** at 10 μM on topotecan cytotoxicity

Table S2. The influence of the deoxycholic acid derivative **5** at 10 μM on topotecan cytotoxicity

Table S3. The binding affinities as predicted by the scoring functions used to the Tdp2 binding site as well as the RMSD values for the co-crystallized ligand (6FQ).

Figure S1. The compounds **4-18** inhibit TDP2 in 1 mM concentration

Figure S2. The influence of derivatives **4-6** and **8-10** (5 μM) on etoposide cytotoxicity against HeLa cells.

Molecular modelling section. A detailed discussion on the molecular modelling results.

Table S4. The binding affinities as predicted by the scoring functions used to the catalytic Tdp1 binding pocket.

Table S5. The molecular descriptors and their corresponding Known Drug Indexes 2a and 2b ($KDI_{2a/2b}$). The R^2 numbers derived do not contain derivatives **13** and **DCA** since they are outlier.

Table S6. Definition of lead-like, drug-like and Known Drug Space (KDS) in terms of molecular descriptors. The values given are the maxima for each descriptor for the volumes of chemical space used.

Table S7. Structures of DCA derivatives. Effect of benzyloxy *vs* acetoxy groups in the steroid scaffold on Tdp1.

Table S8. Structures of DCA derivatives. Effect of benzyloxy *vs* methoxy groups in the steroid scaffold on Tdp1.

Spectrum of Compound **1**, ^{1}H NMR, 500MHz, $CDCl_3$

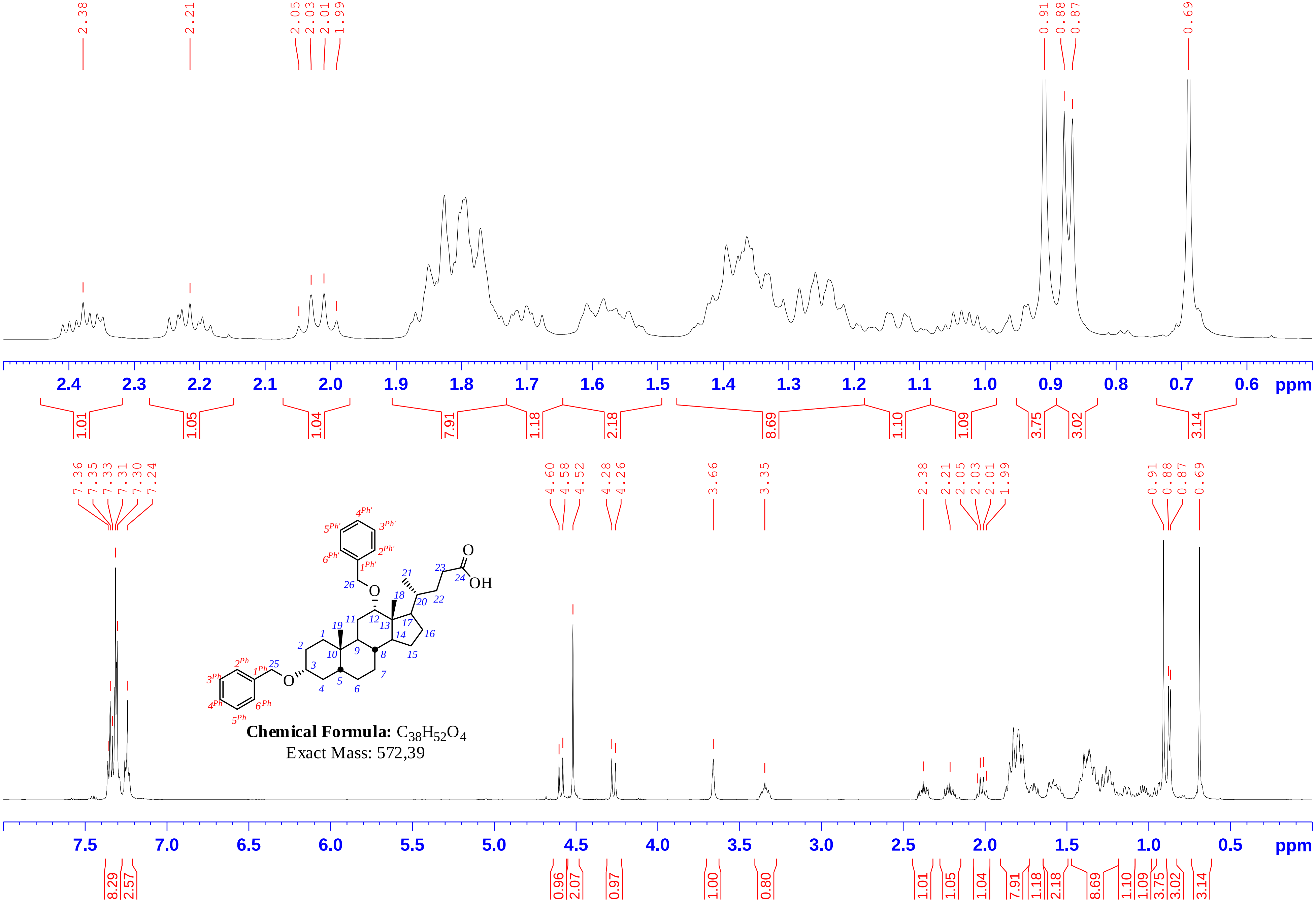

Spectrum of Compound **1**, ^{13}C NMR, JMOD, 125MHz, $CDCl_3$

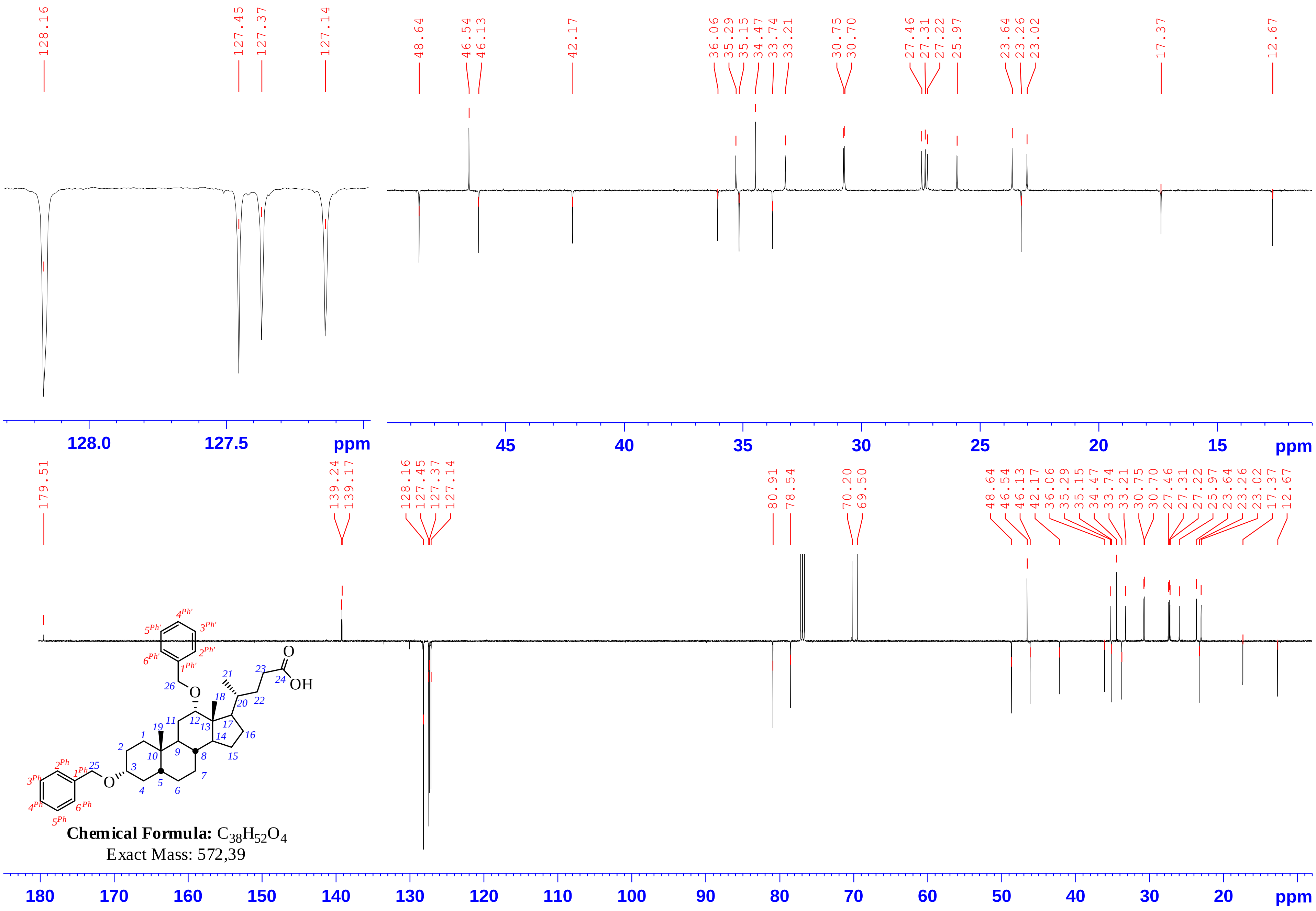

High resolution mass spectrum of compound **1**, Tsource=110°C, Tprobe=250°C

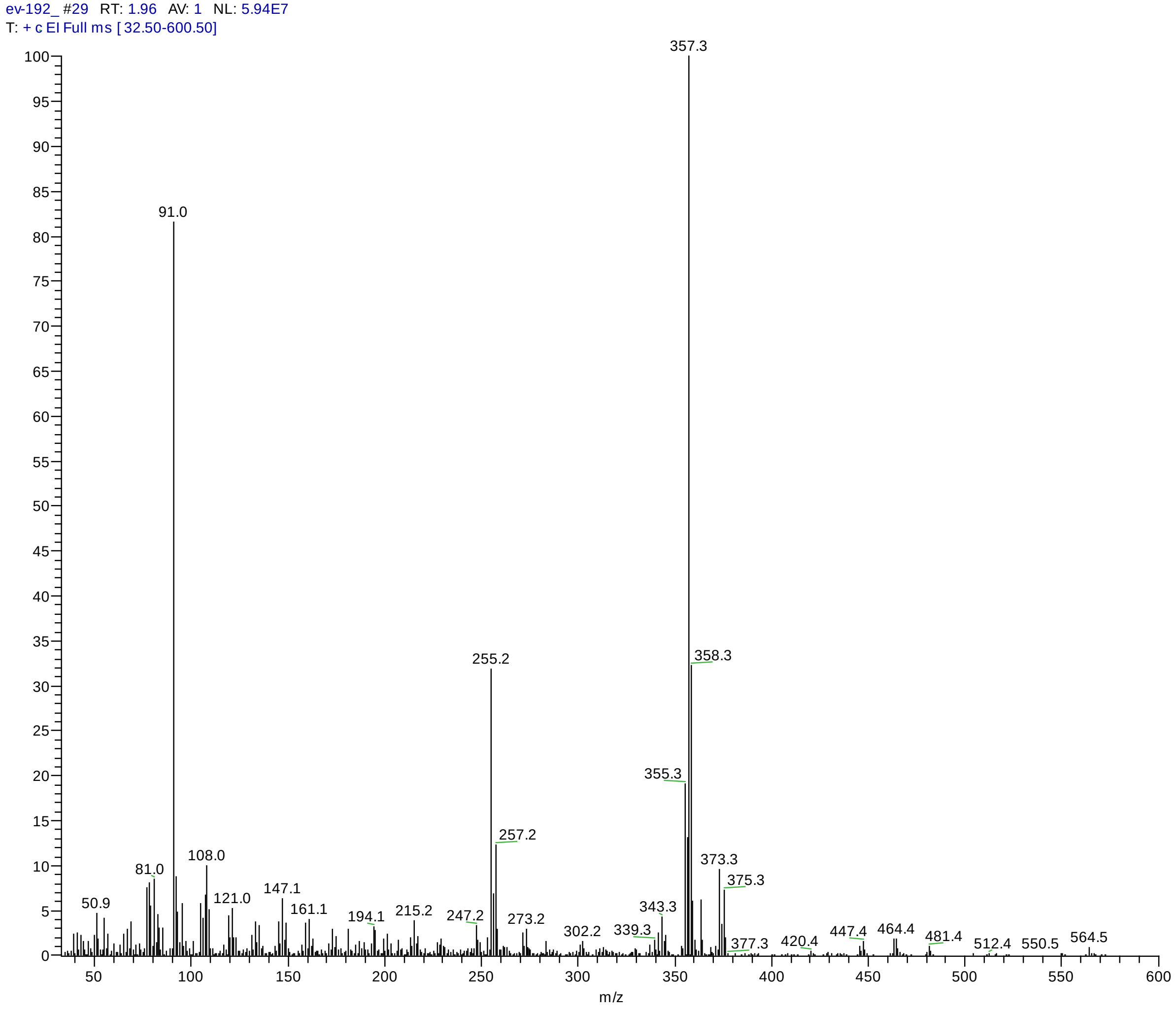

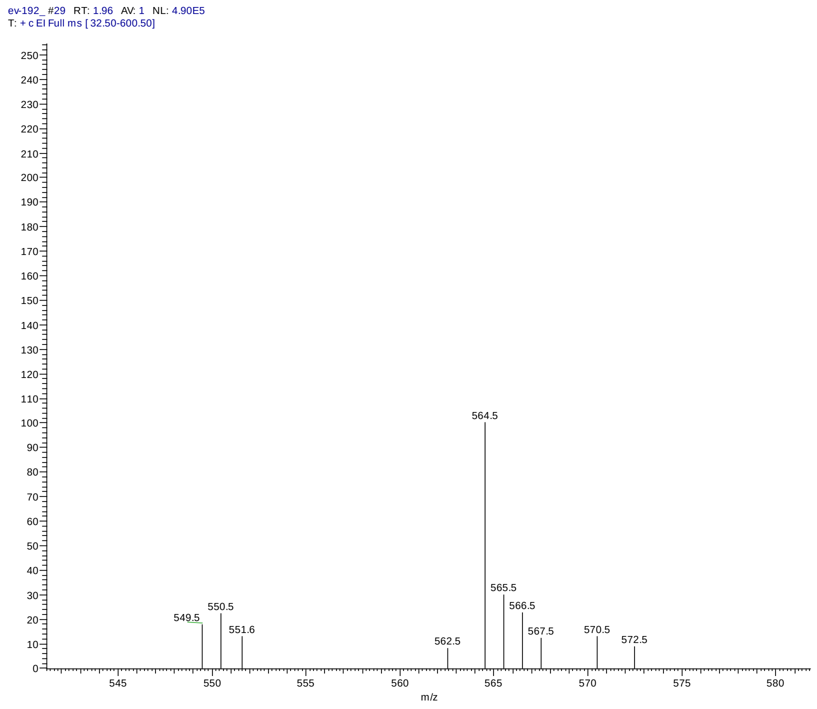

Calculated m/z= 570.3704 $(C_{38}H_{50}O_4)^+$

Found m/z= 570.3712

Spectrum of Compound **2**, ^{1}H NMR, 500MHz, $CDCl_3$

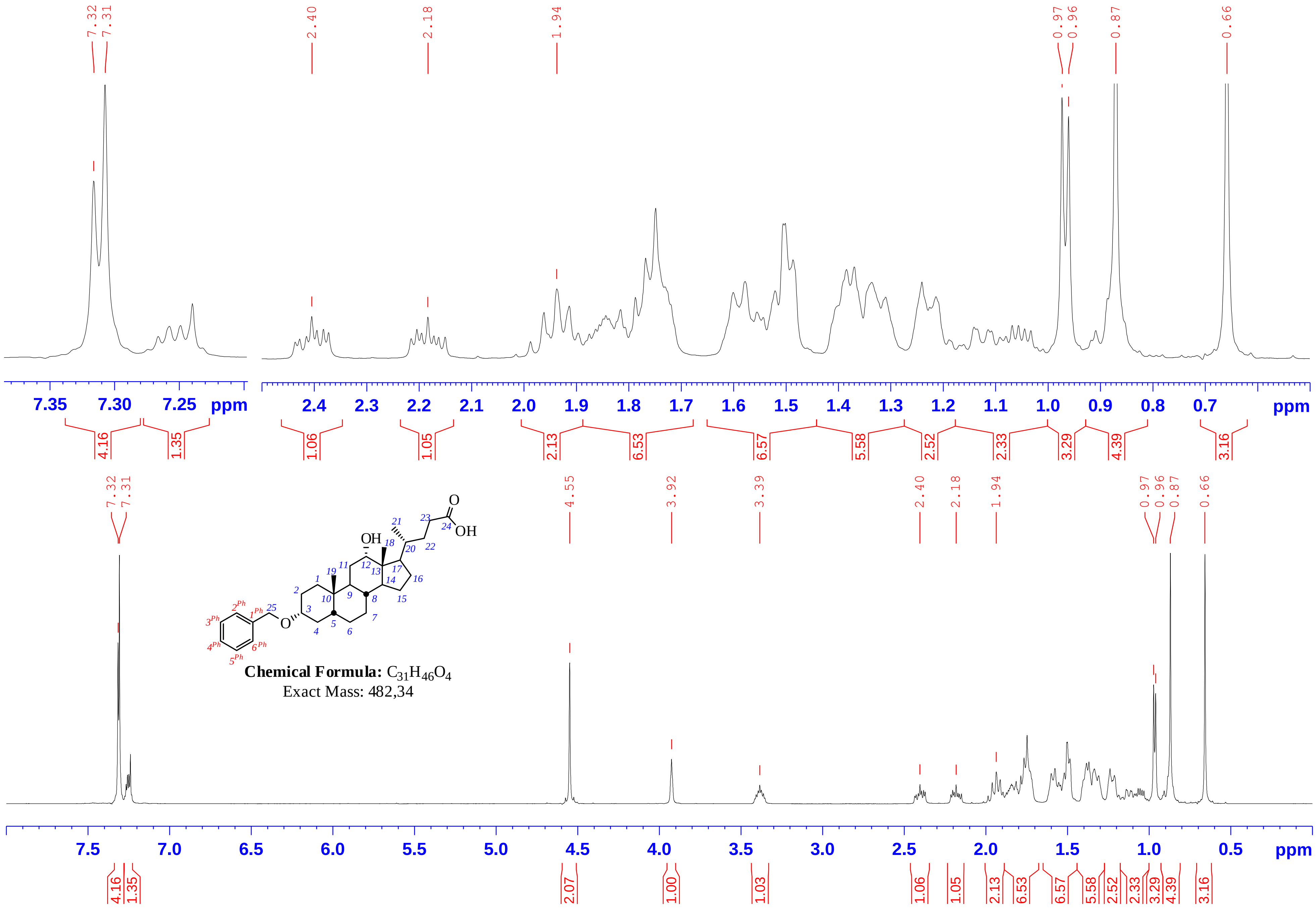

Spectrum of Compound **2**, ^{13}C NMR, JMOD, 125MHz, $CDCl_3$

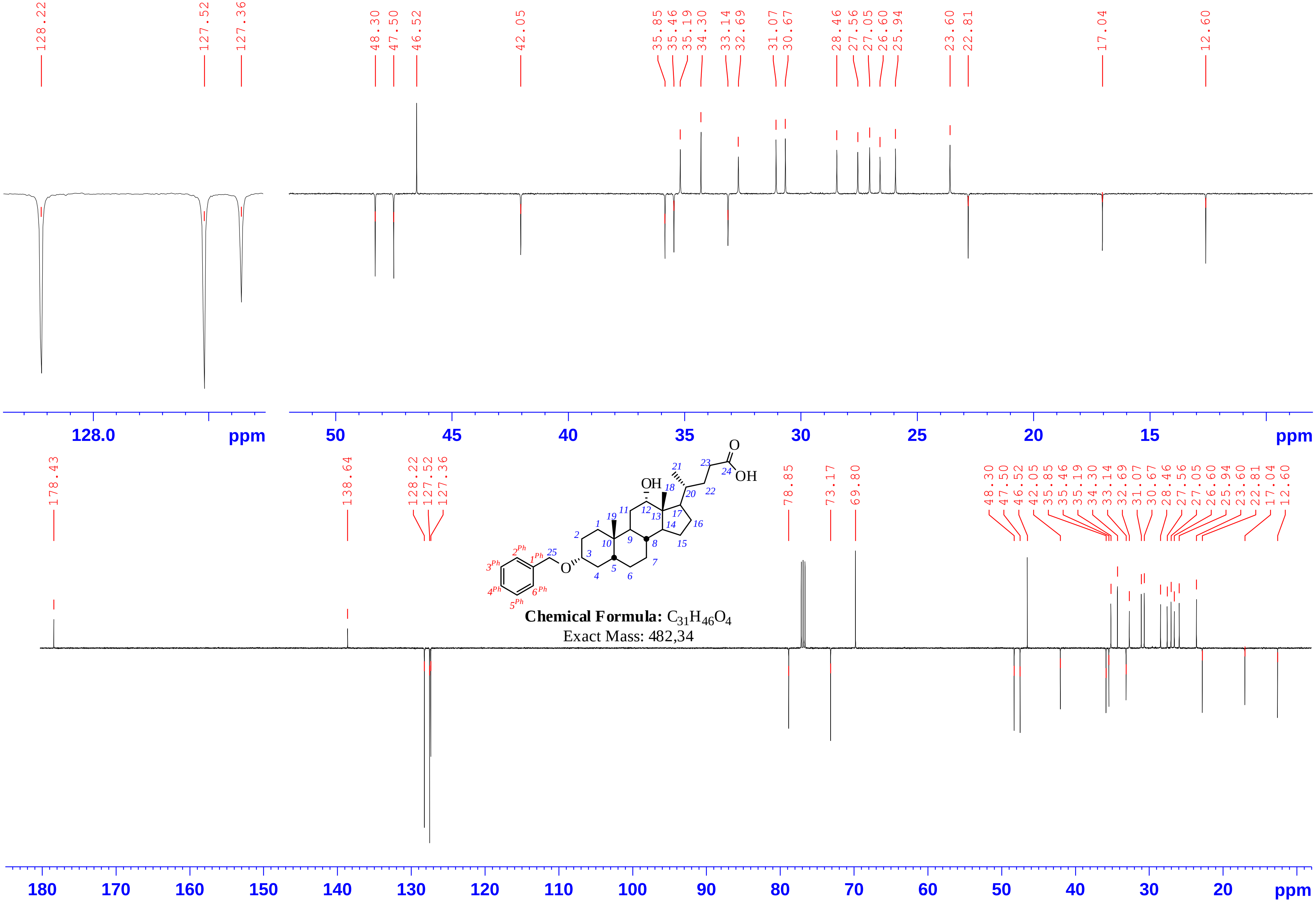

High resolution mass spectrum of compound **2**, Tsource=100°C, Tprobe=290°C

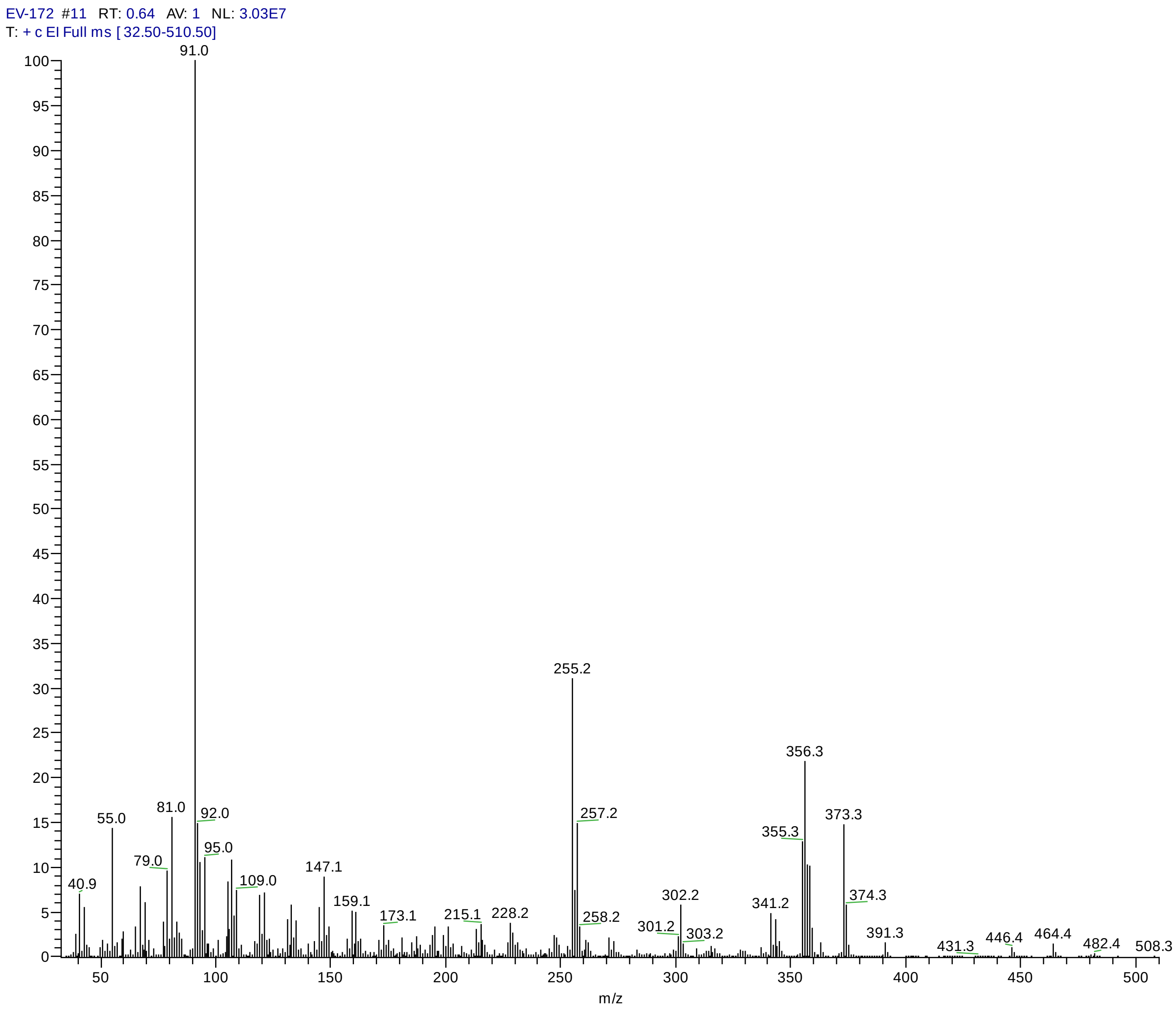

Calculated m/z= 482.3391 ($C_{31}H_{46}O_4$)$^{+\cdot}$

Found m/z= 482.3379

Spectrum of Compound **3**, ^{1}H NMR, 500MHz, $CDCl_3$

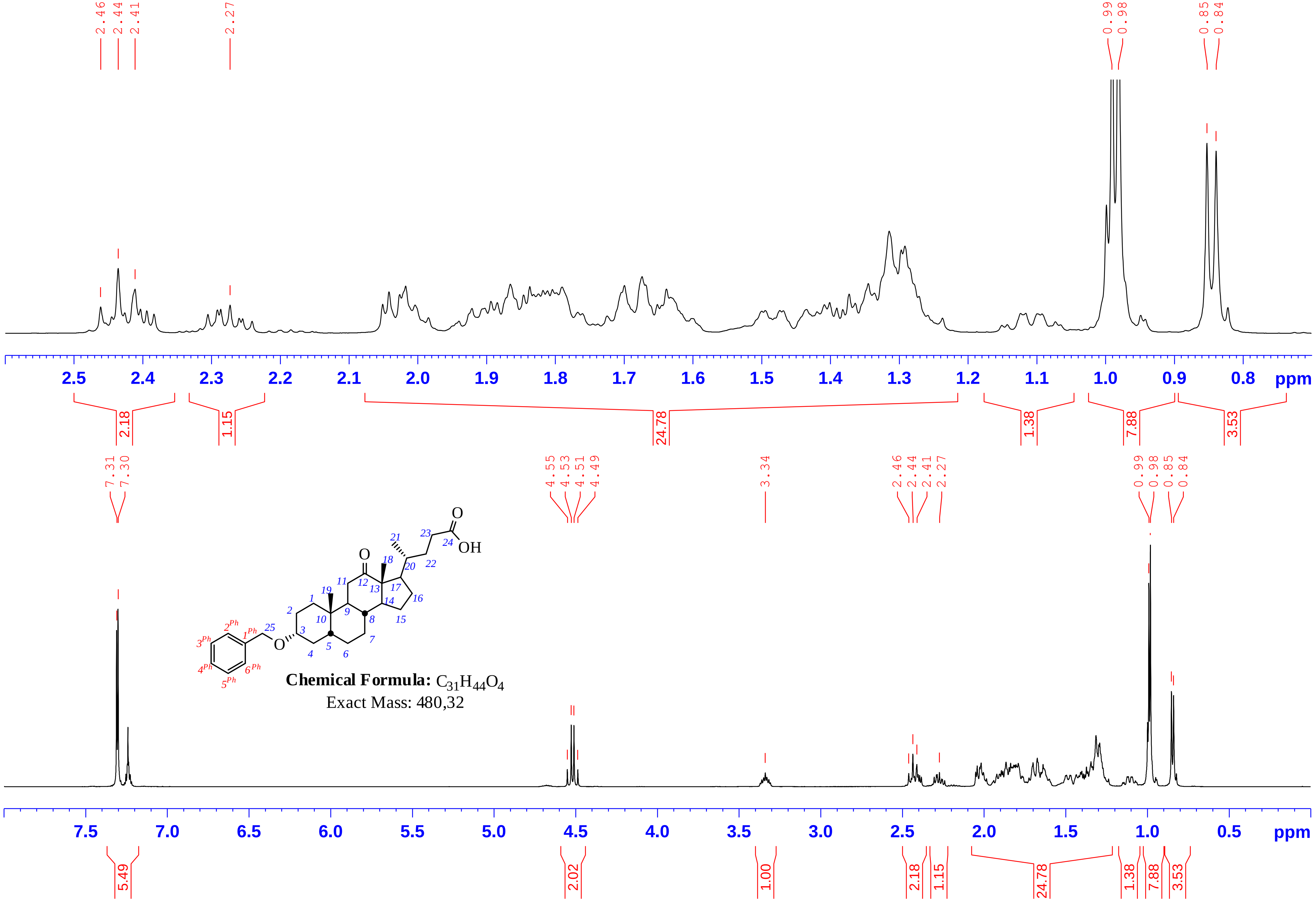

Spectrum of Compound **3**, ^{13}C NMR, JMOD, 125MHz, $CDCl_3$

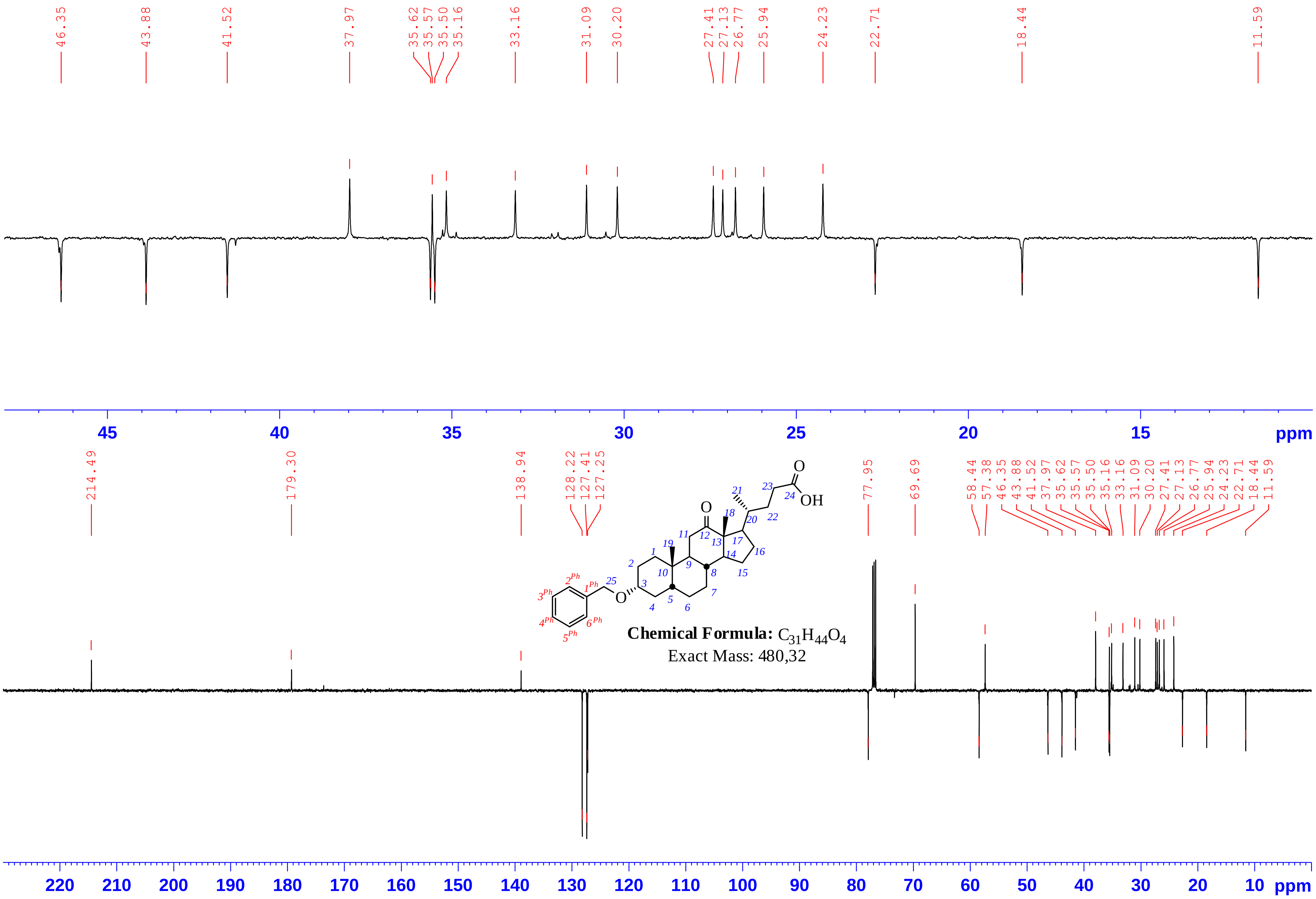

Chemical Formula: $C_{31}H_{44}O_4$

Exact Mass: 480,32

High resolution mass spectrum of compound **3**, Tsource=100°C, Tprobe=280°C

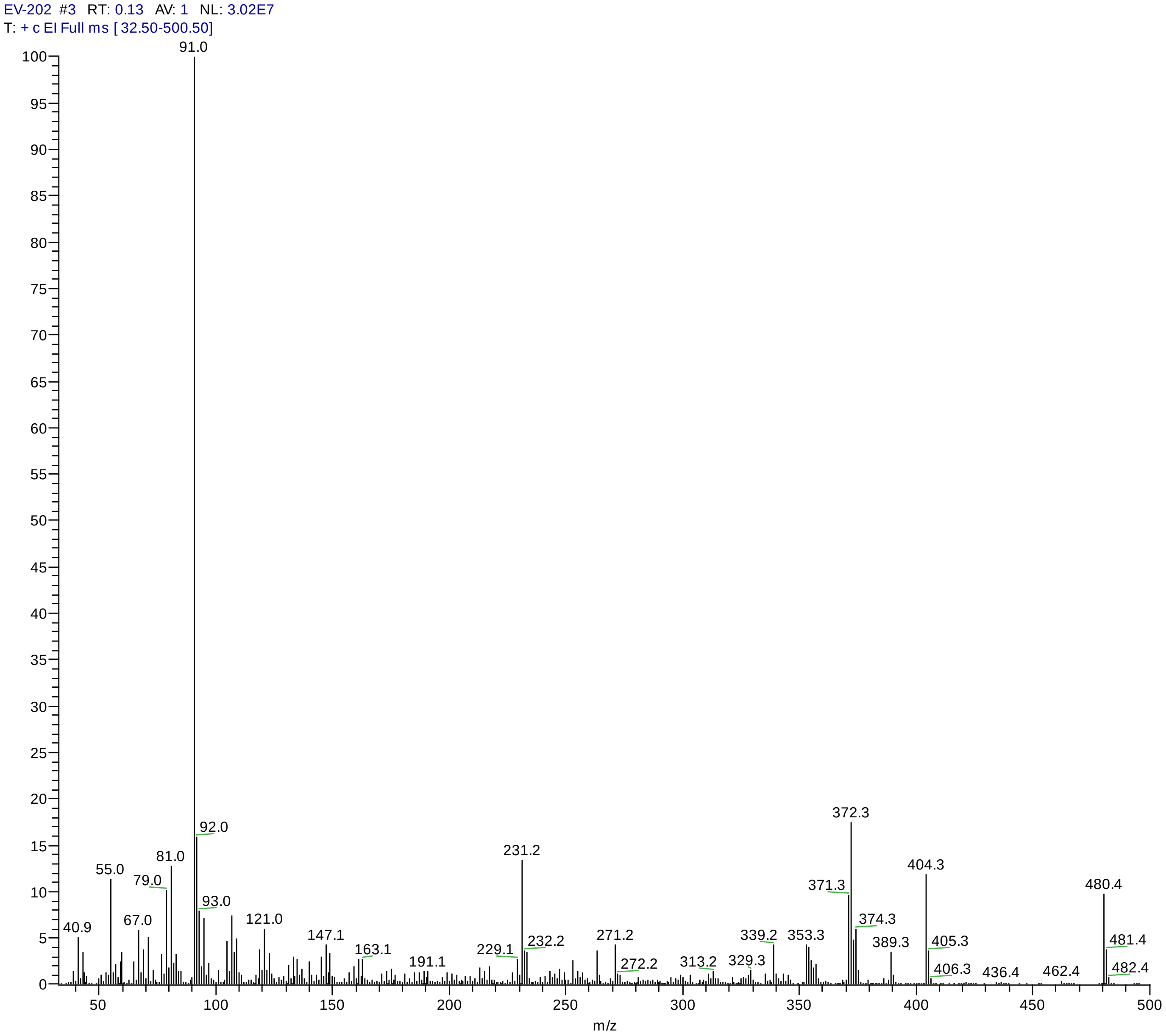

Calculated m/z= 480.3234 ($C_{31}H_{44}O_4$)$^+$

Found m/z= 480.3231

Spectrum of Compound **4**, ^{1}H NMR, 500MHz, $CDCl_3$

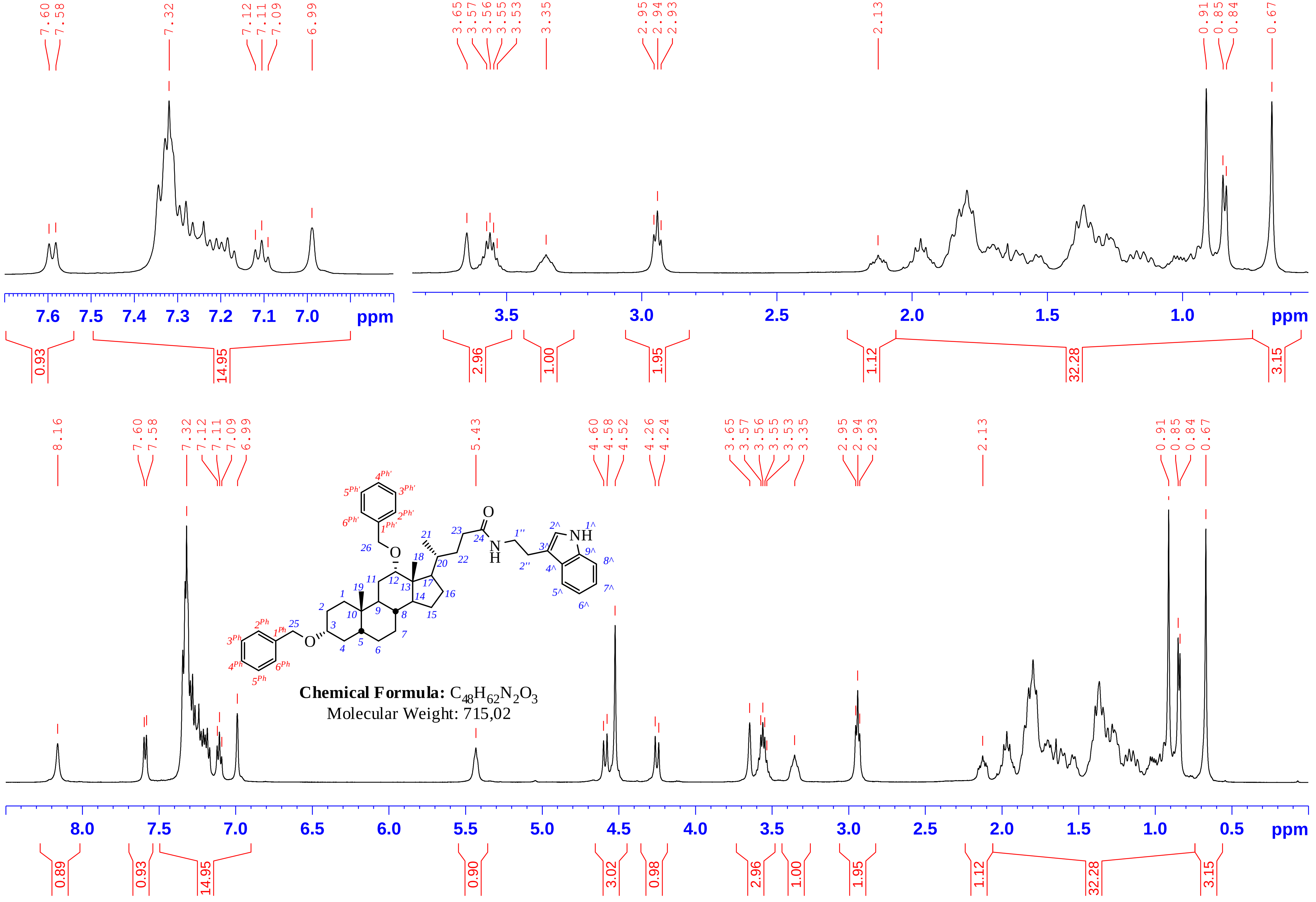

Spectrum of Compound **4**, ^{13}C NMR, JMOD, 125MHz, $CDCl_3$

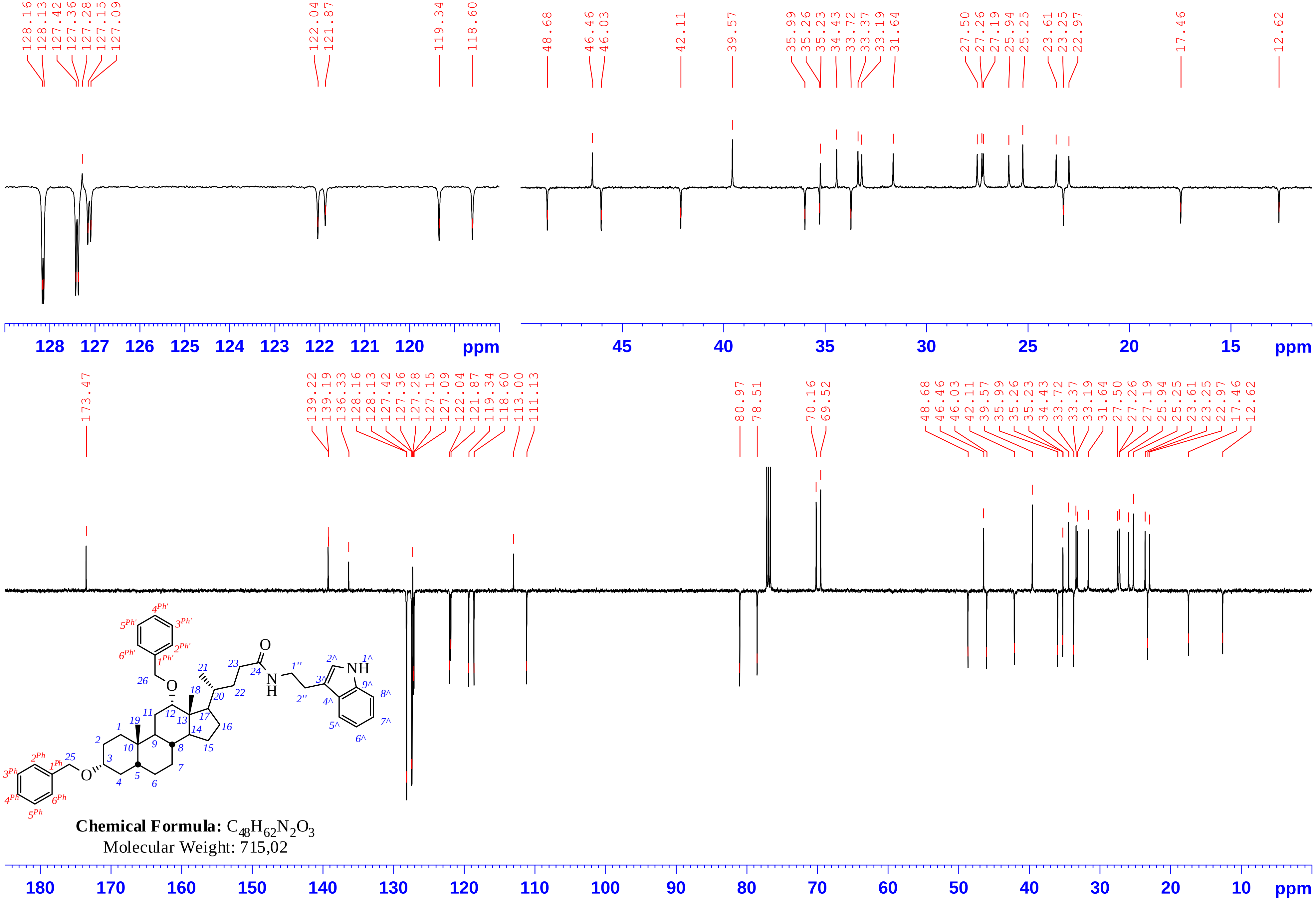

Chemical Formula: $C_{48}H_{62}N_2O_3$

Molecular Weight: 715,02

High resolution mass spectrum of compound **4**, Tsource=80°C, Tprobe=340°C

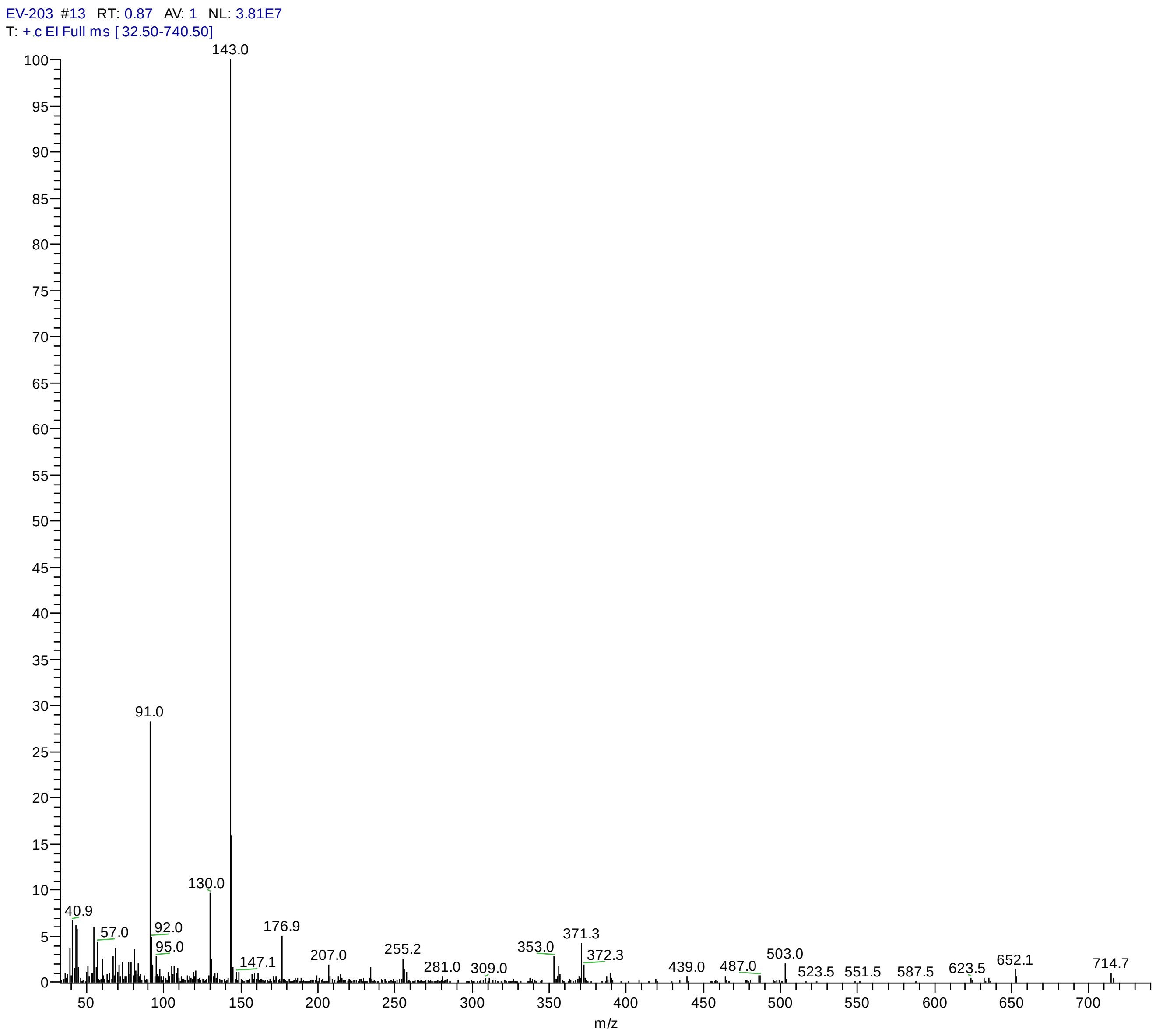

Calculated m/z= 714.4755 $(C_{48}H_{62}O_3N_2)^+$

Found m/z= 714.4748

Spectrum of Compound **5**, ^{1}H NMR, 300MHz, $CDCl_3$

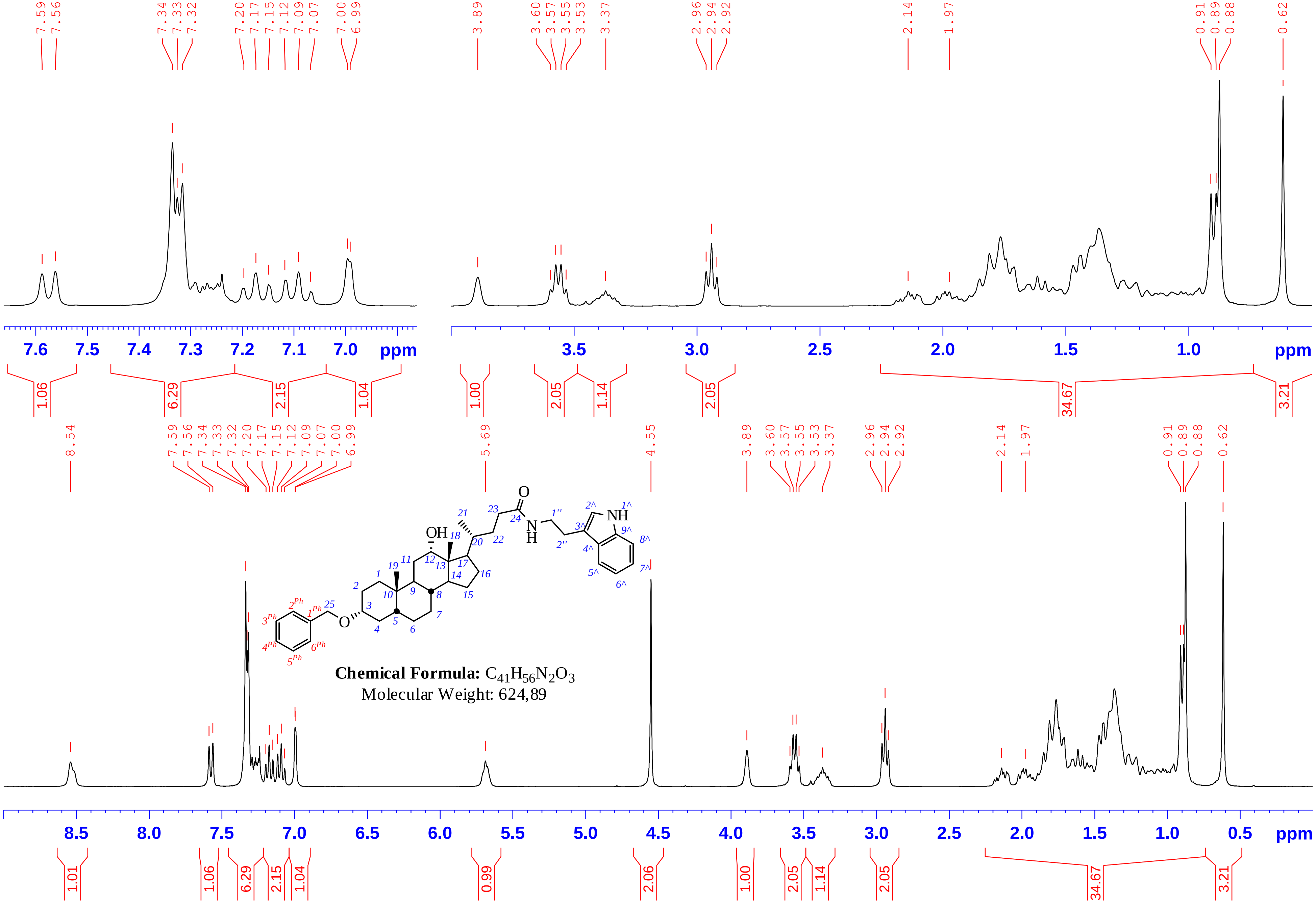

Spectrum of Compound **5**, ^{13}C NMR, JMOD, 75MHz, $CDCl_3$

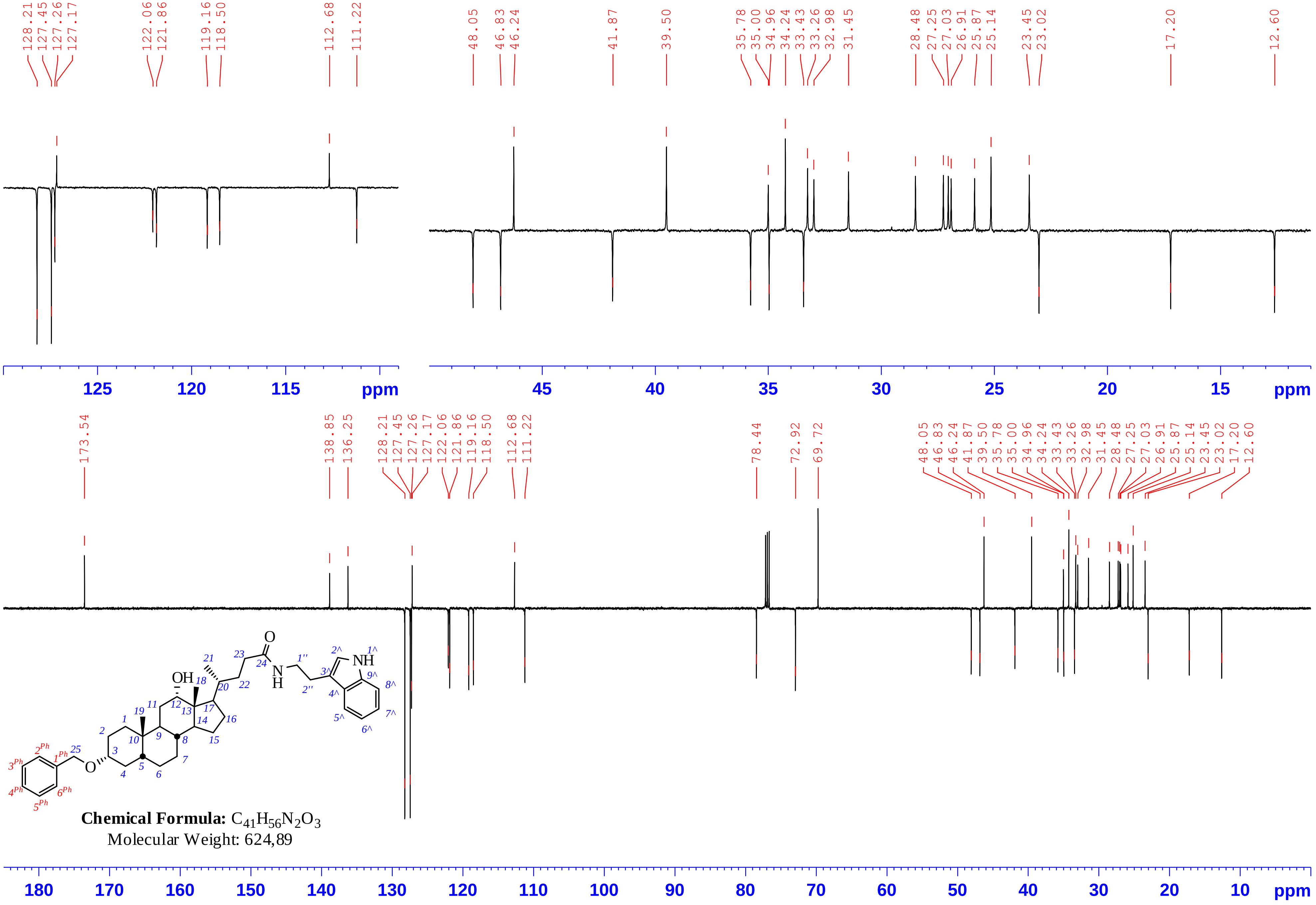

High resolution mass spectrum of compound **5**, Tsource=100°C, Tprobe=300°C

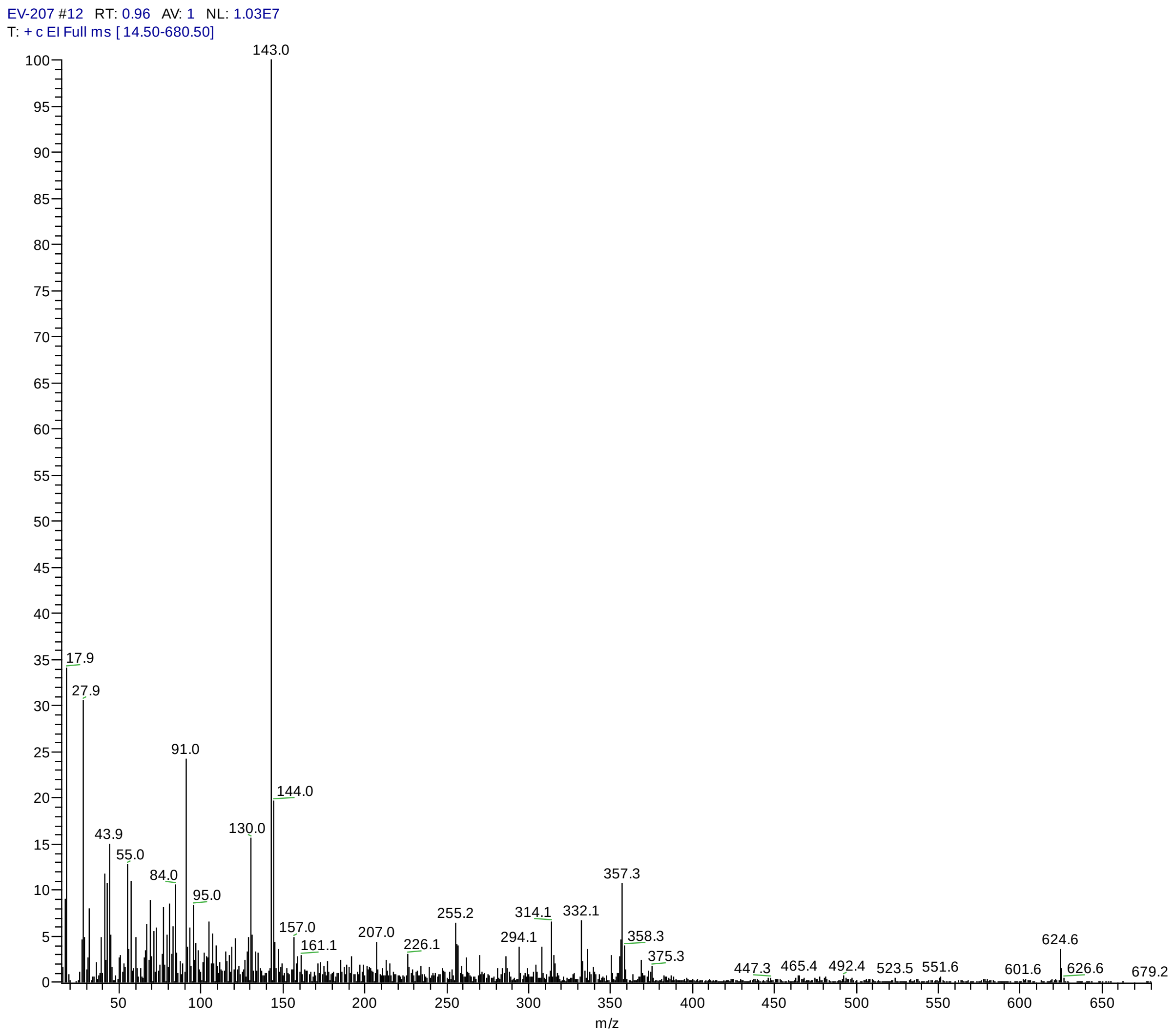

Calculated m/z= 624.4286 $(C_{41}H_{56}O_3N_2)^+$

Found m/z= 624.4285

Spectrum of Compound **6**, ^{1}H NMR, 500MHz, $CDCl_3$

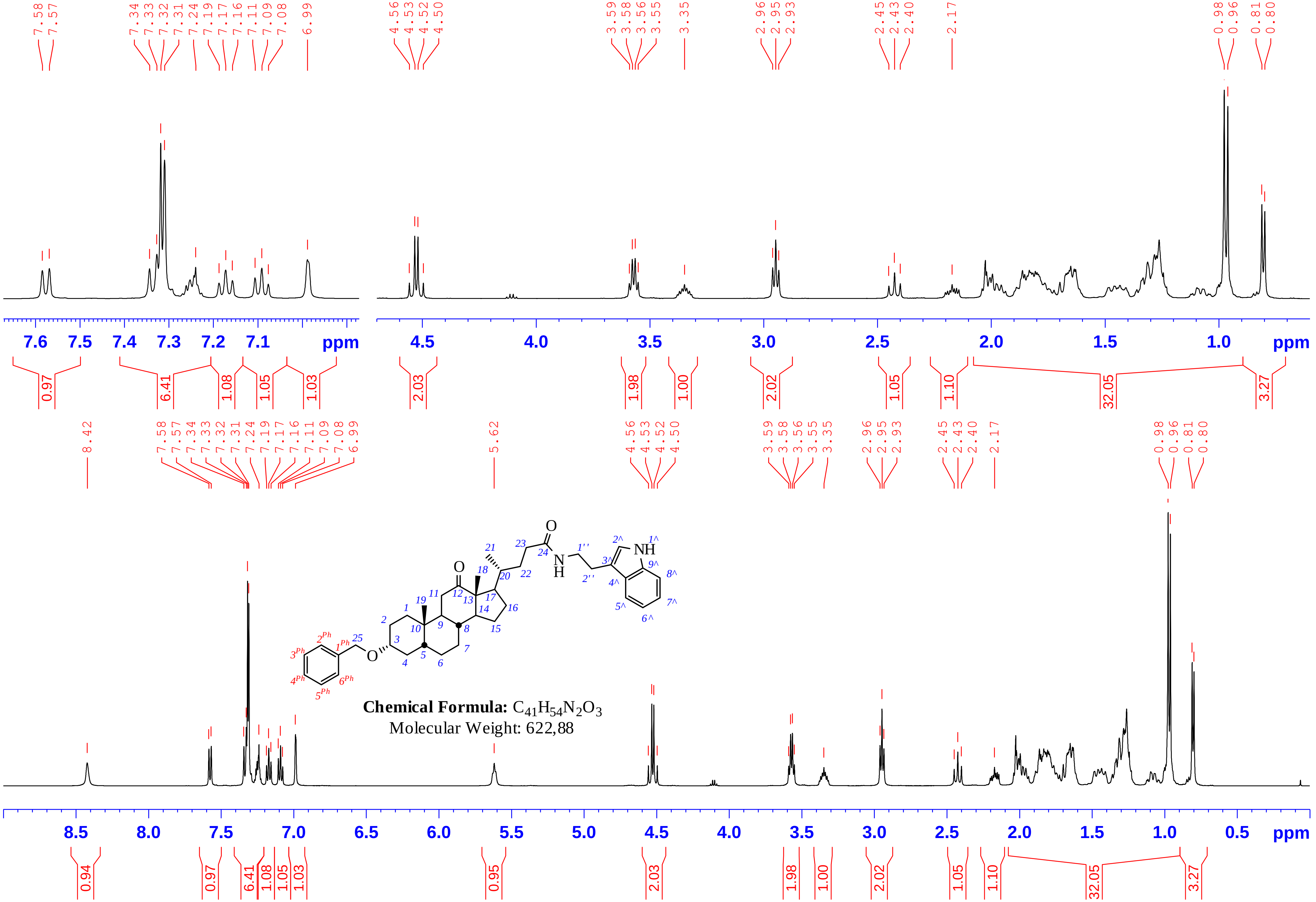

Spectrum of Compound **6**, ^{13}C NMR, JMOD, 125MHz, $CDCl_3$

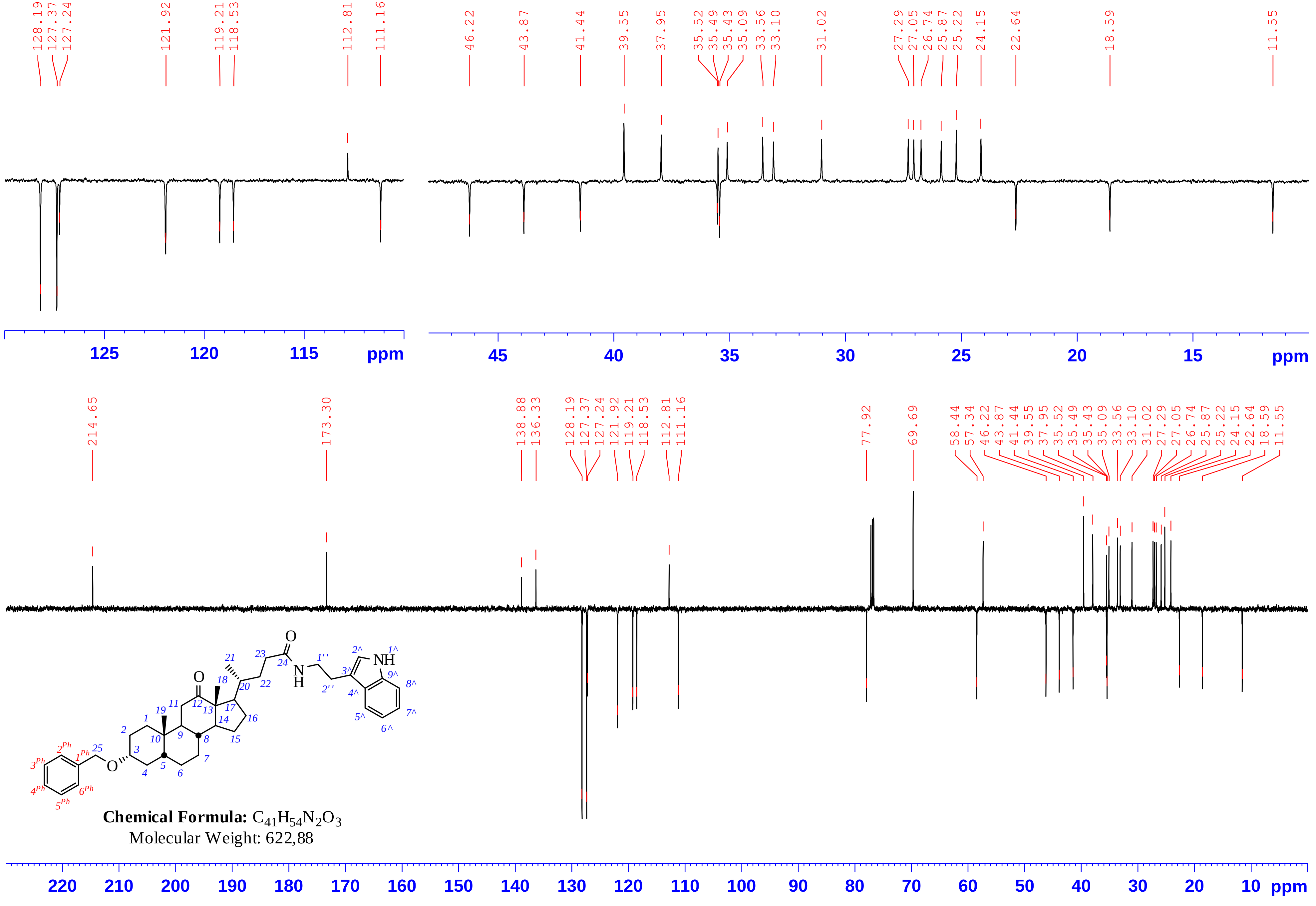

Chemical Formula: $C_{41}H_{54}N_2O_3$

Molecular Weight: 622,88

High resolution mass spectrum of compound **6**, Tsource=100°C, Tprobe=345°C

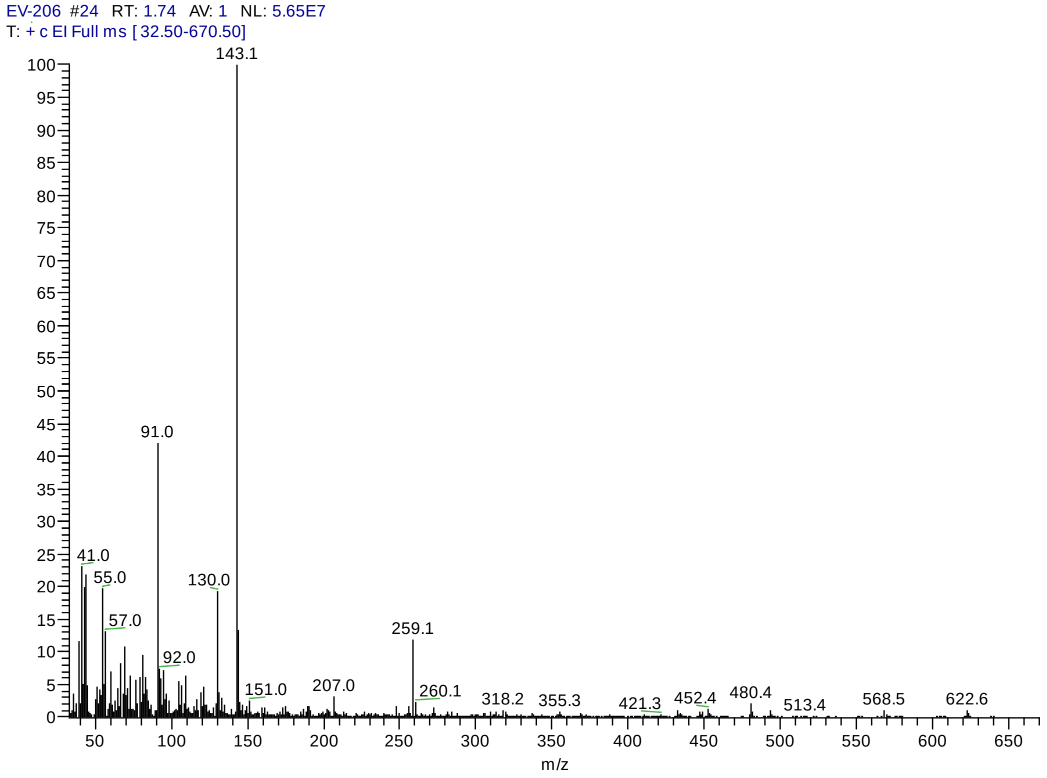

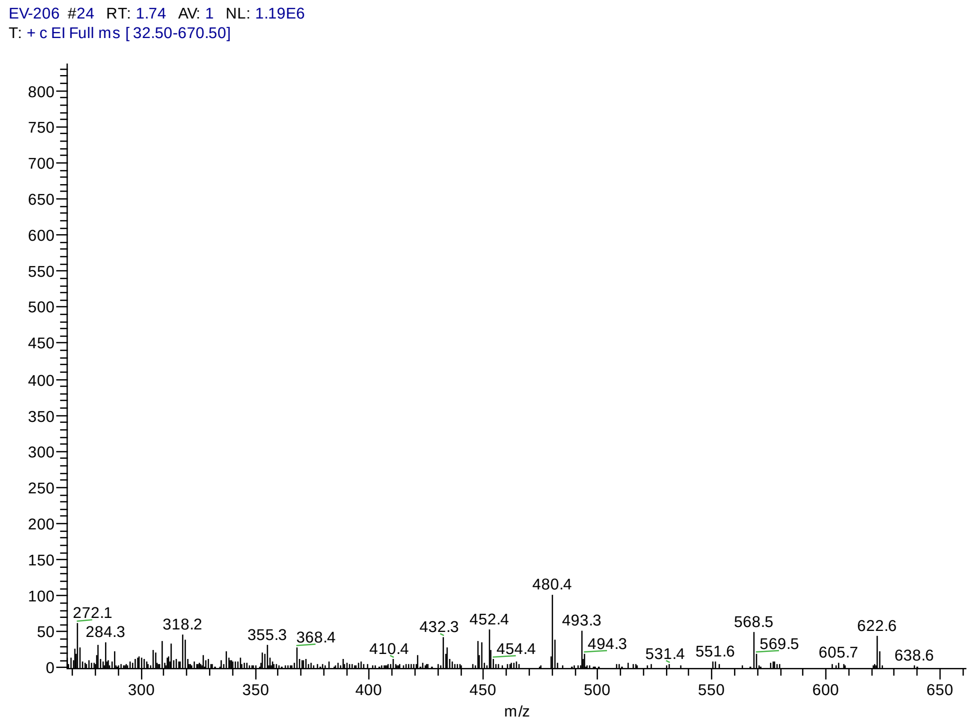

Calculated m/z= 622.4129 $(C_{41}H_{54}O_3N_2)^+$

Found m/z= 622.4124

Spectrum of Compound **7**, ^{1}H NMR, 400MHz, $CDCl_3$

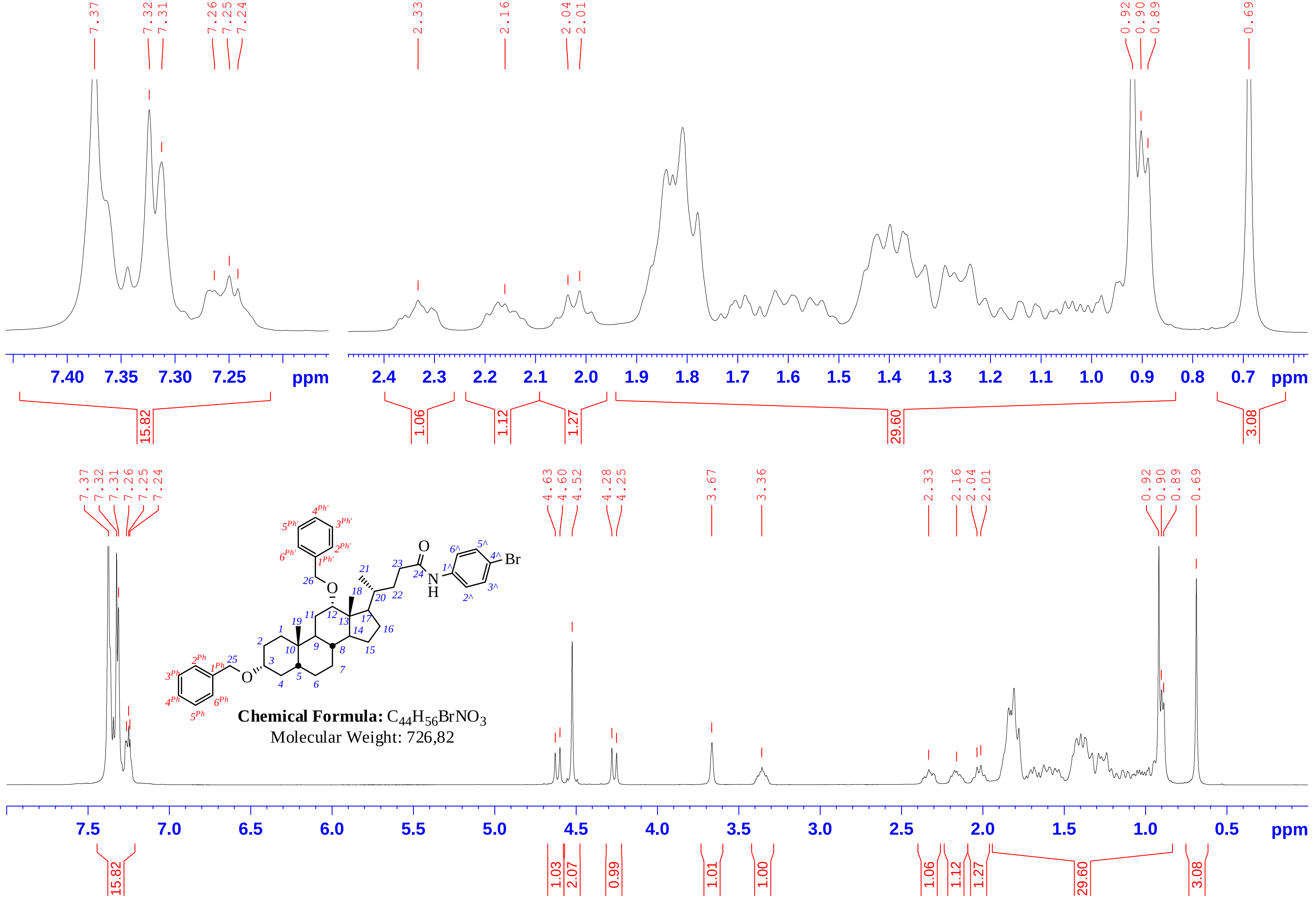

Spectrum of Compound **7**, ^{13}C NMR, JMOD, 100MHz, $CDCl_3$

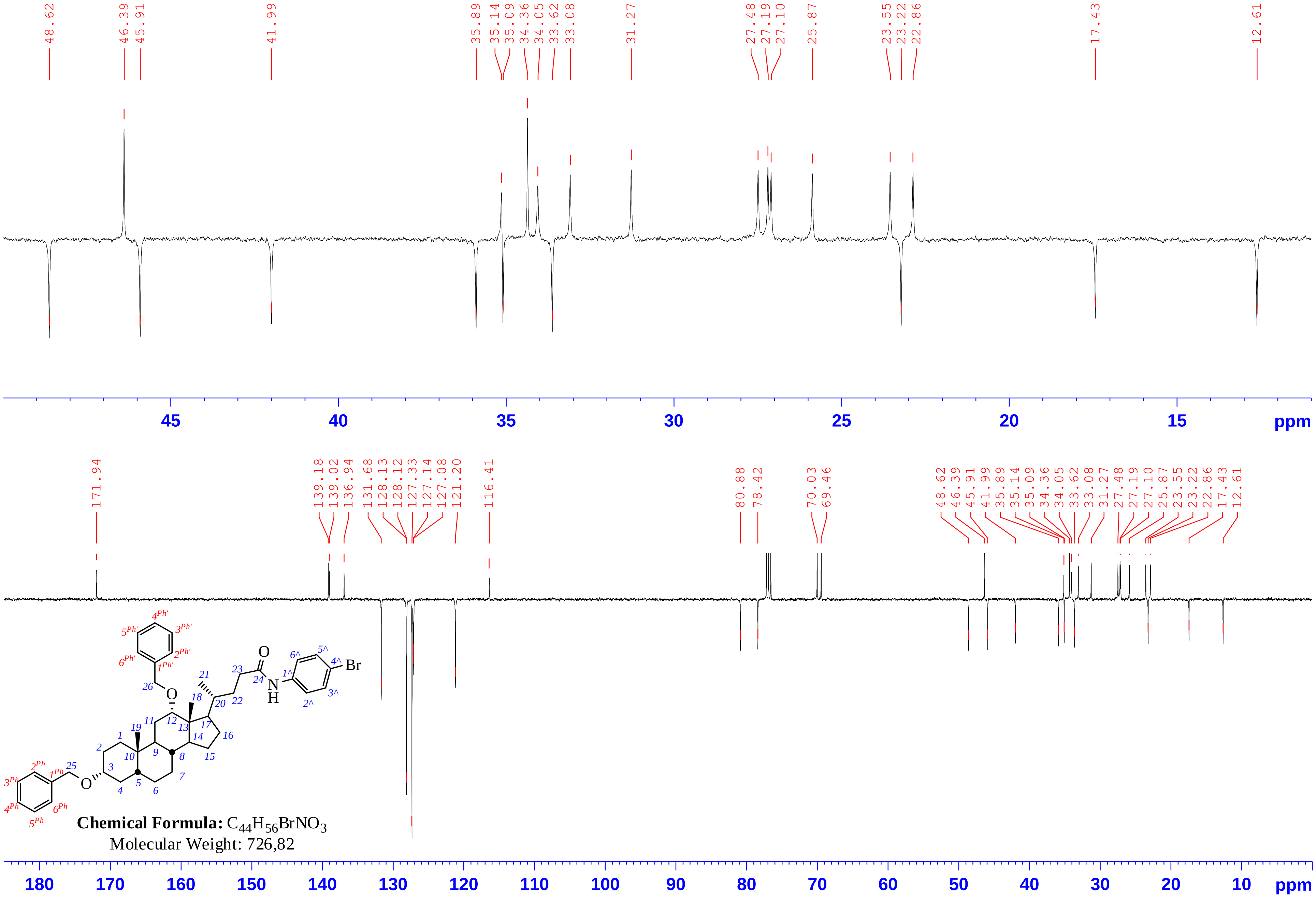

High resolution mass spectrum of compound **7**, Tsource=100°C, Tprobe=290°C

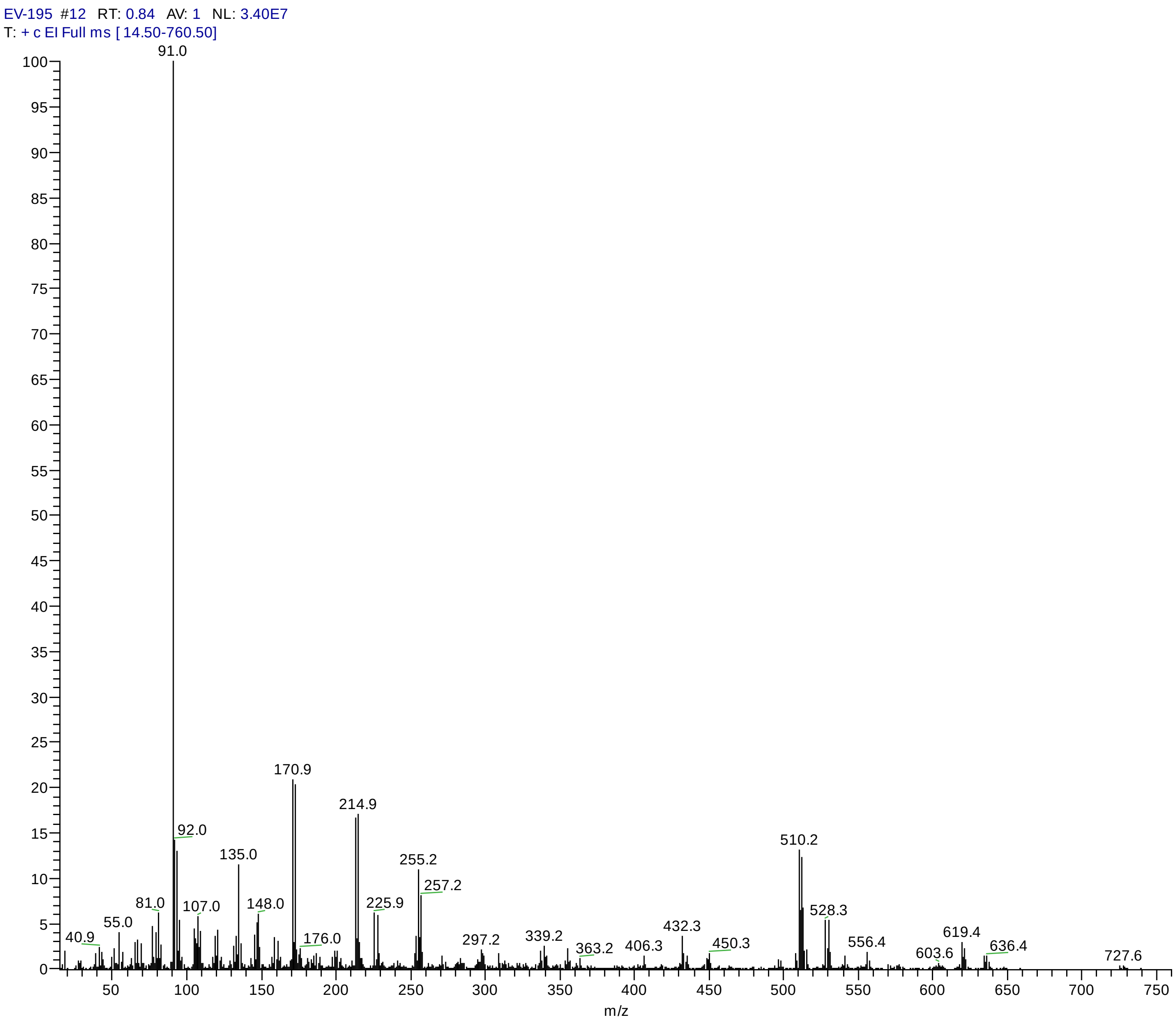

Calculated m/z= 725.3438 $(C_{44}H_{56}O_3N_1{}^{79}Br_1)^+$

Found m/z= 725.3444

IR spectrum of compound **7**

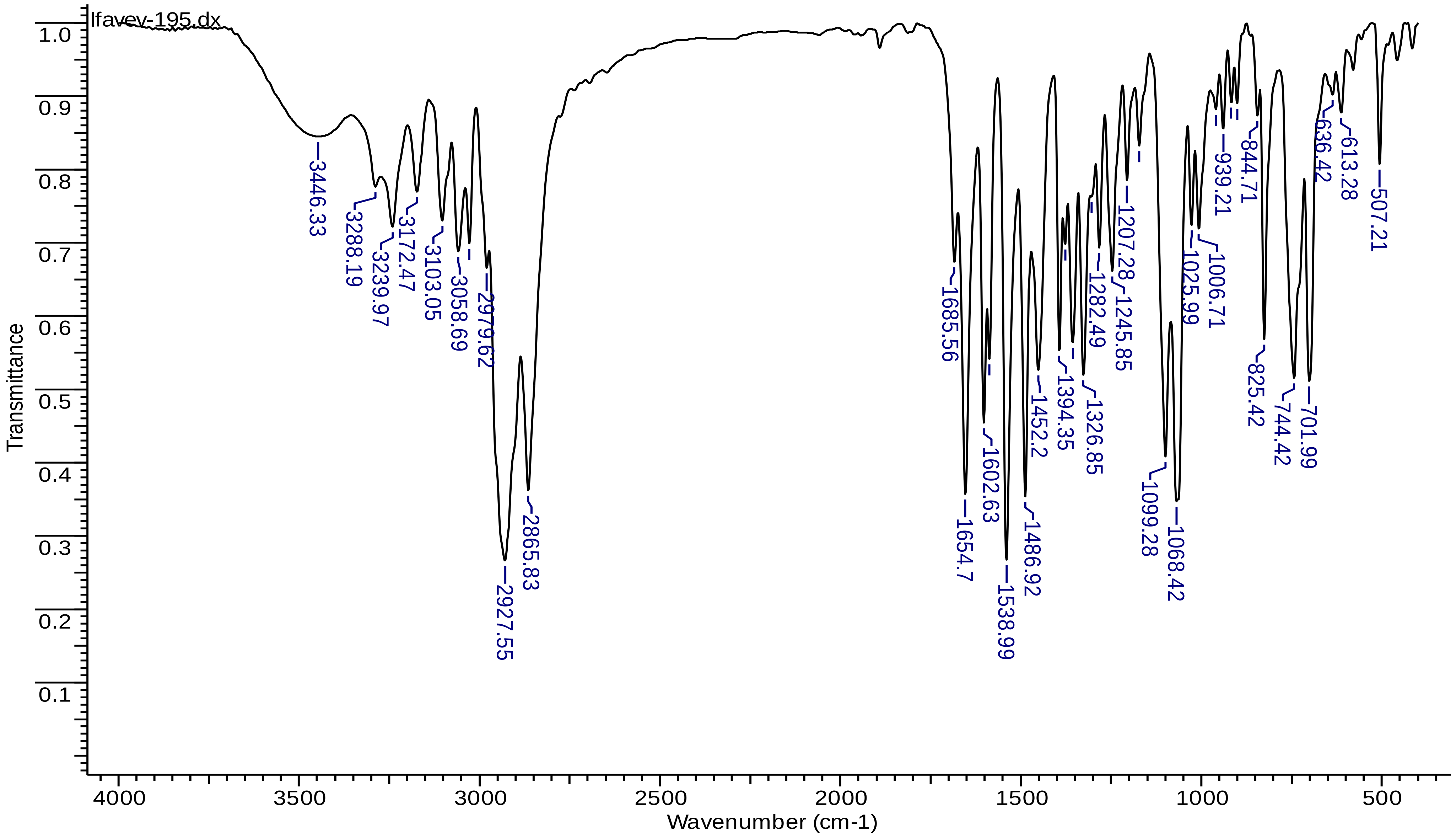

IR, ν, cm^{-1}: 1654 (C=O st (amide))

Spectrum of Compound **8**, ^{1}H NMR, 500MHz, $CDCl_3$

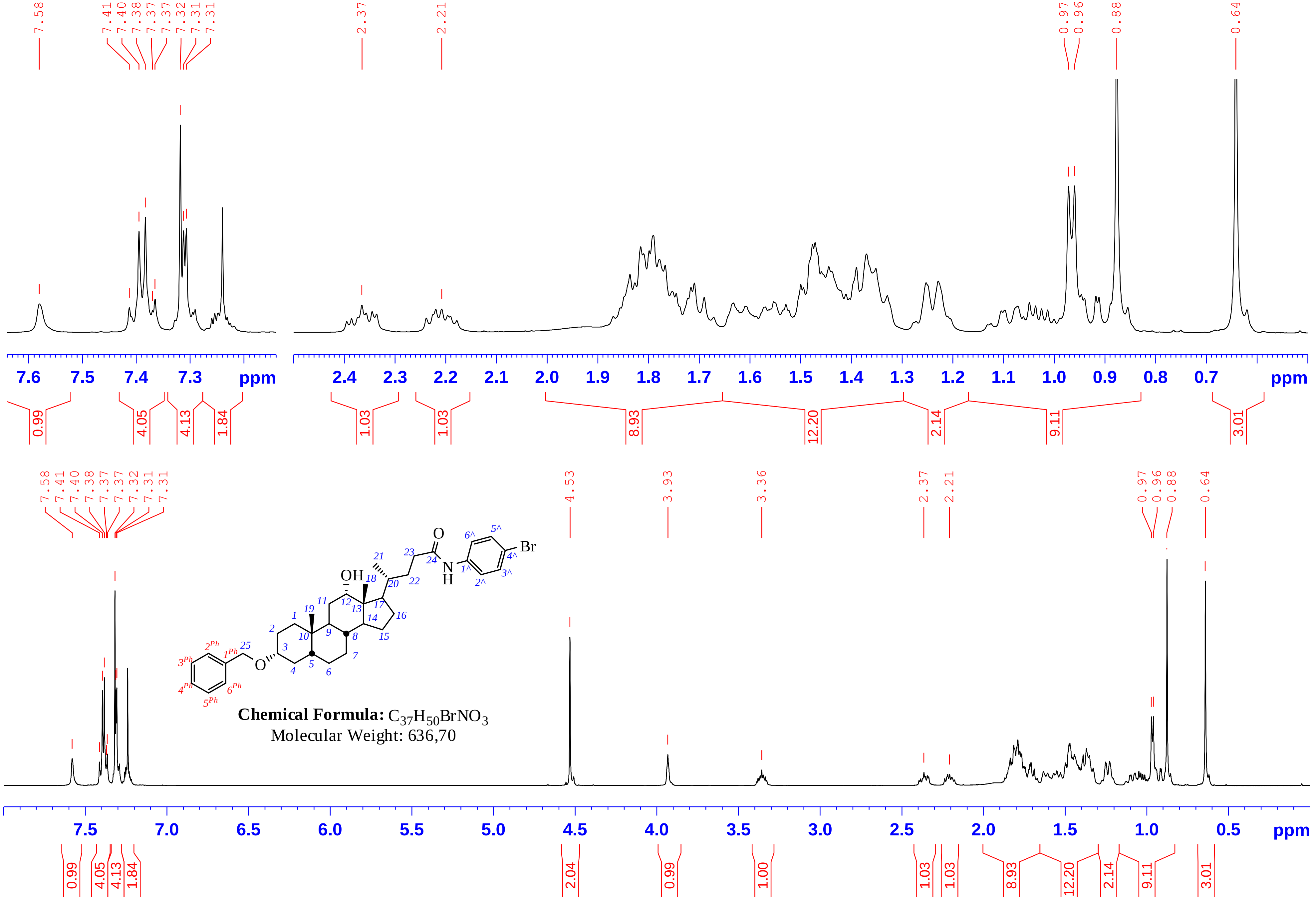

Spectrum of Compound **8**, ^{13}C NMR, JMOD, 125MHz, $CDCl_3$

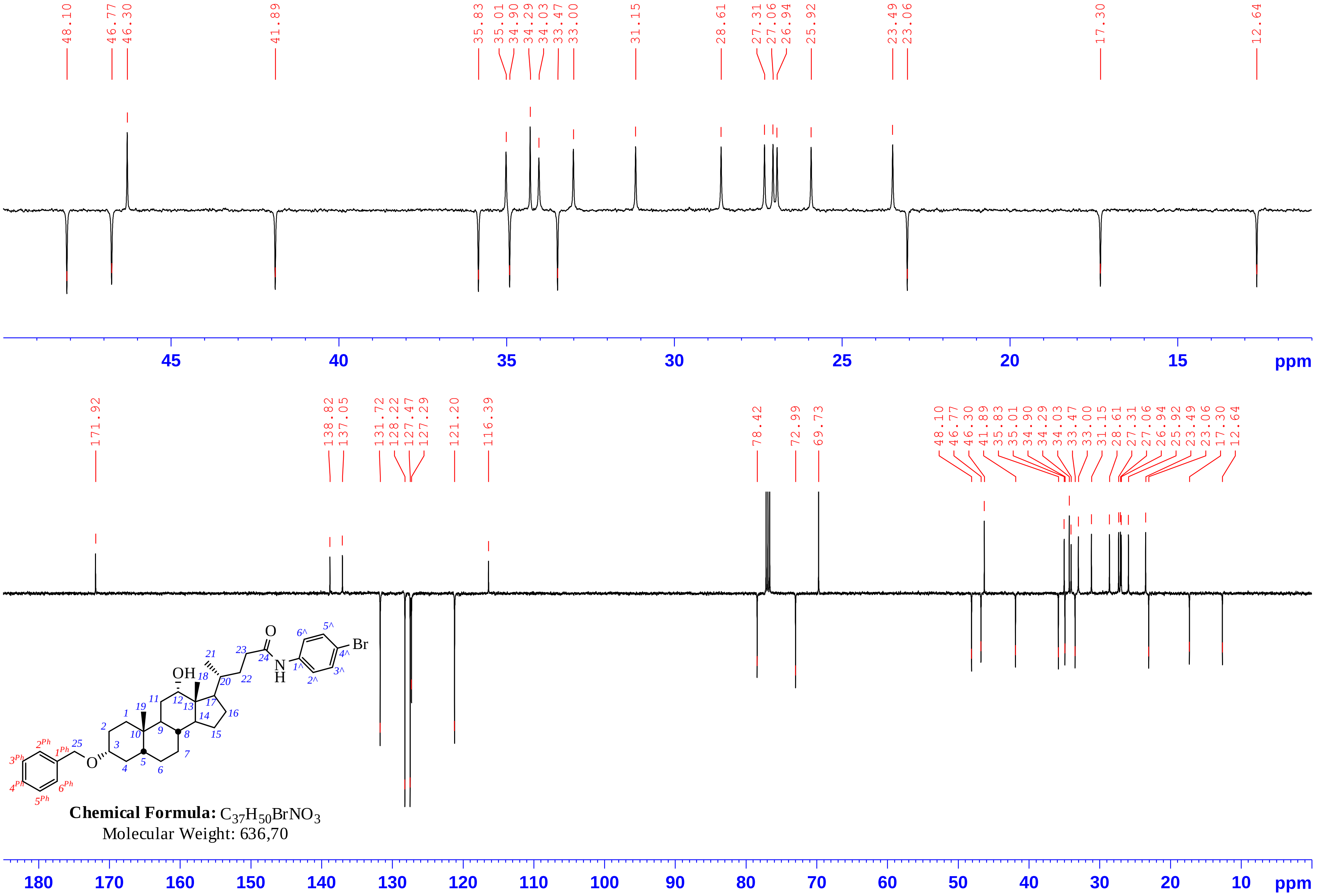

Chemical Formula: $C_{37}H_{50}BrNO_3$
Molecular Weight: 636,70

High resolution mass spectrum of compound **8**, Tsource=100°C, Tprobe=300°C

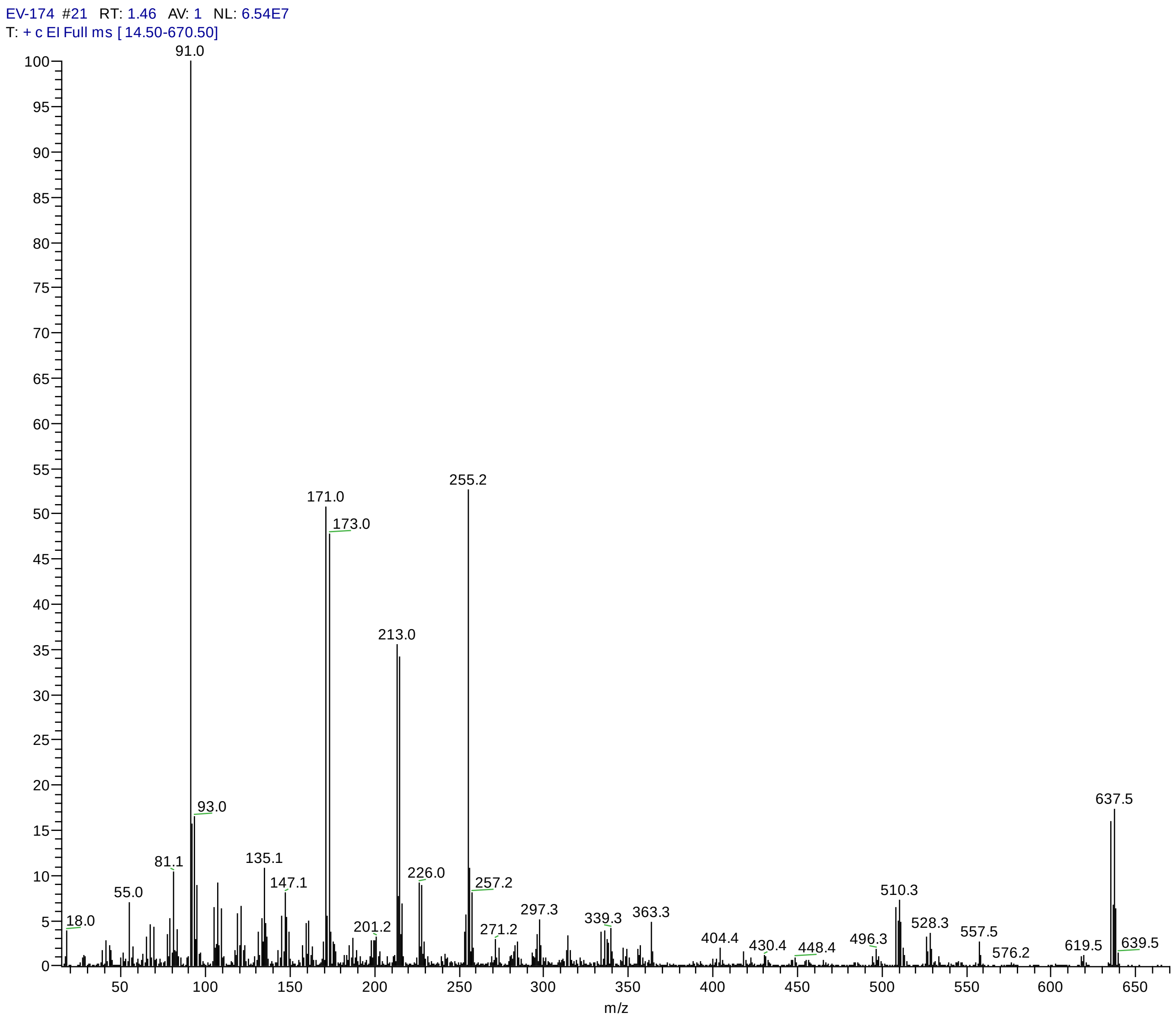

Calculated m/z= 635.2969 ($(C_{37}H_{50}O_{3}N_{1}{}^{79}Br_{1})^{+}$)

Found m/z= 635.2963

Spectrum of Compound **9**, ^{1}H NMR, 500MHz, $CDCl_3$

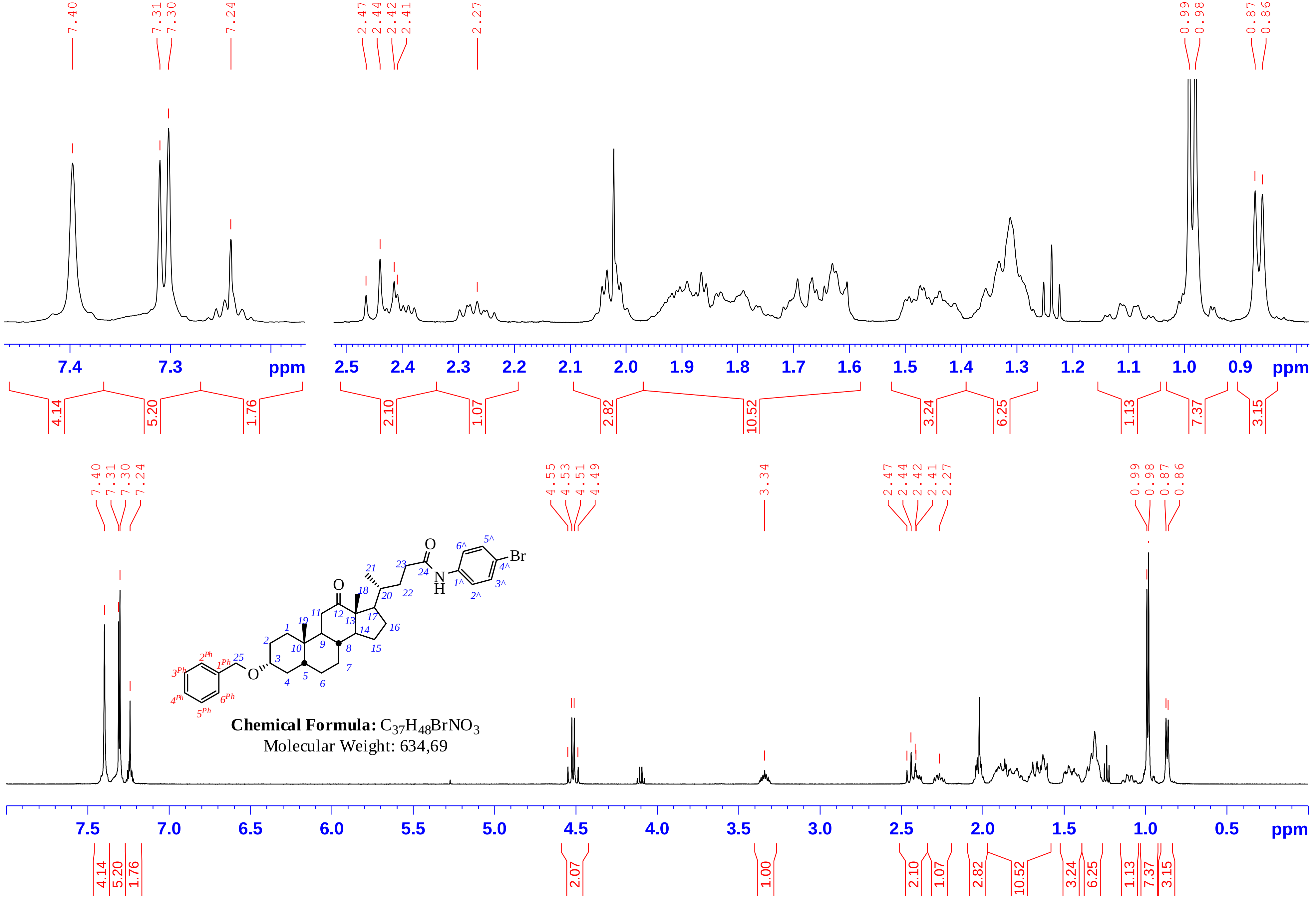

Spectrum of Compound **9**, ^{13}C NMR, JMOD, 125MHz, $CDCl_3$

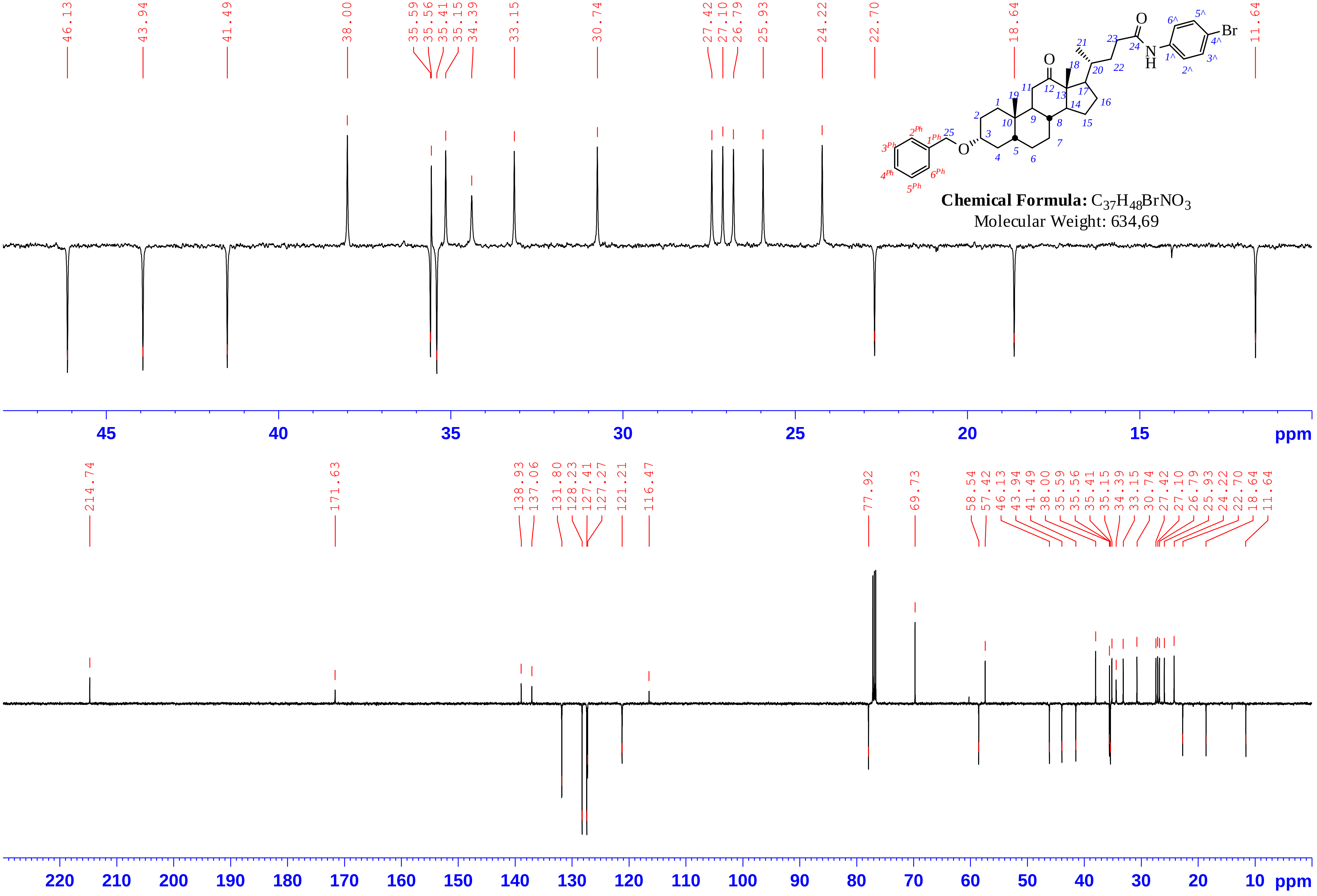

Chemical Formula: $C_{37}H_{48}BrNO_3$

Molecular Weight: 634,69

High resolution mass spectrum of compound **9**, Tsource=50°C, Tprobe=3300°C

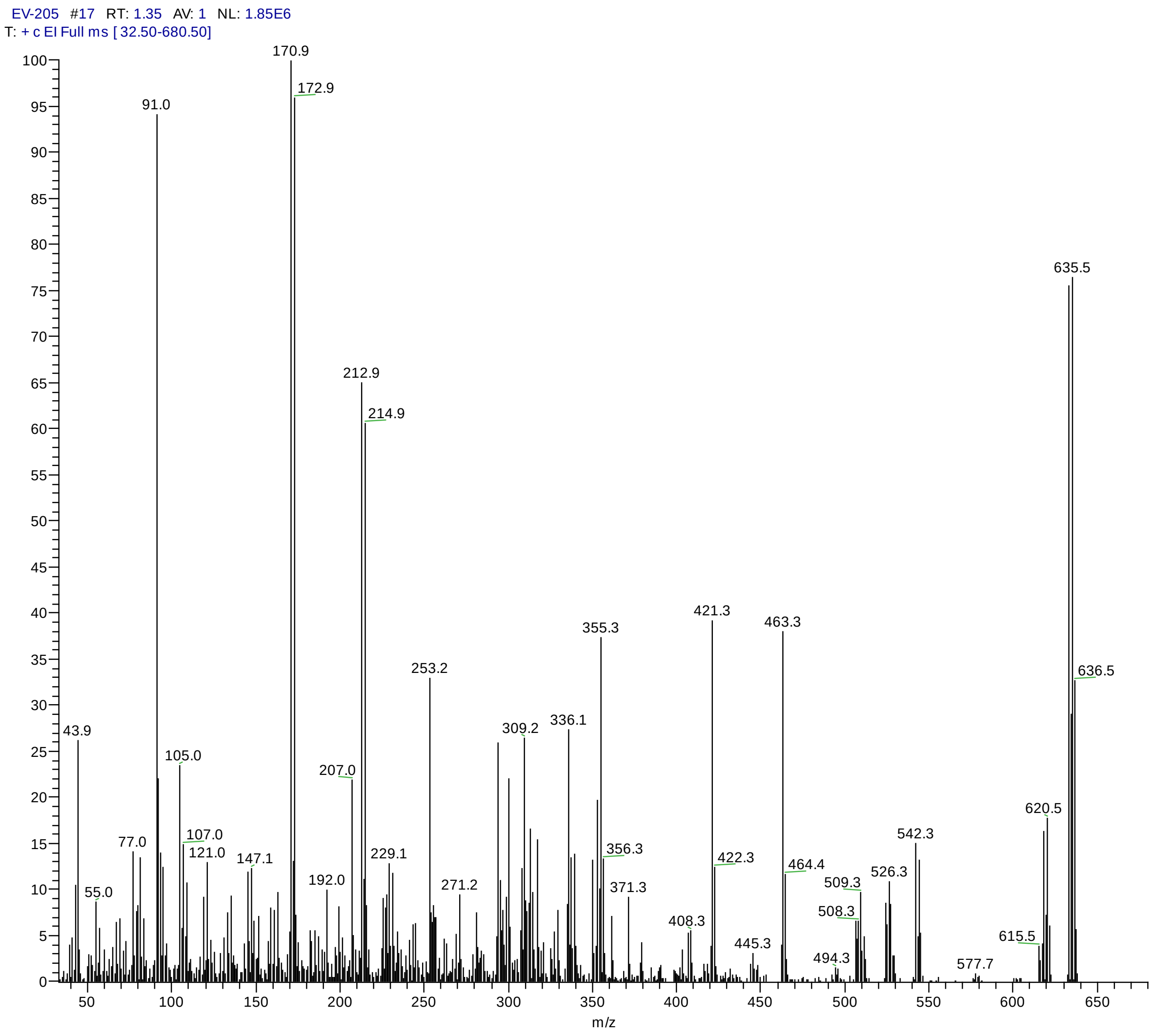

Calculated m/z= 633.2812 ($(C_{37}H_{48}O_3N_1{}^{79}Br_1)^+$)

Found m/z= 633.2824

IR spectrum of compound **9**

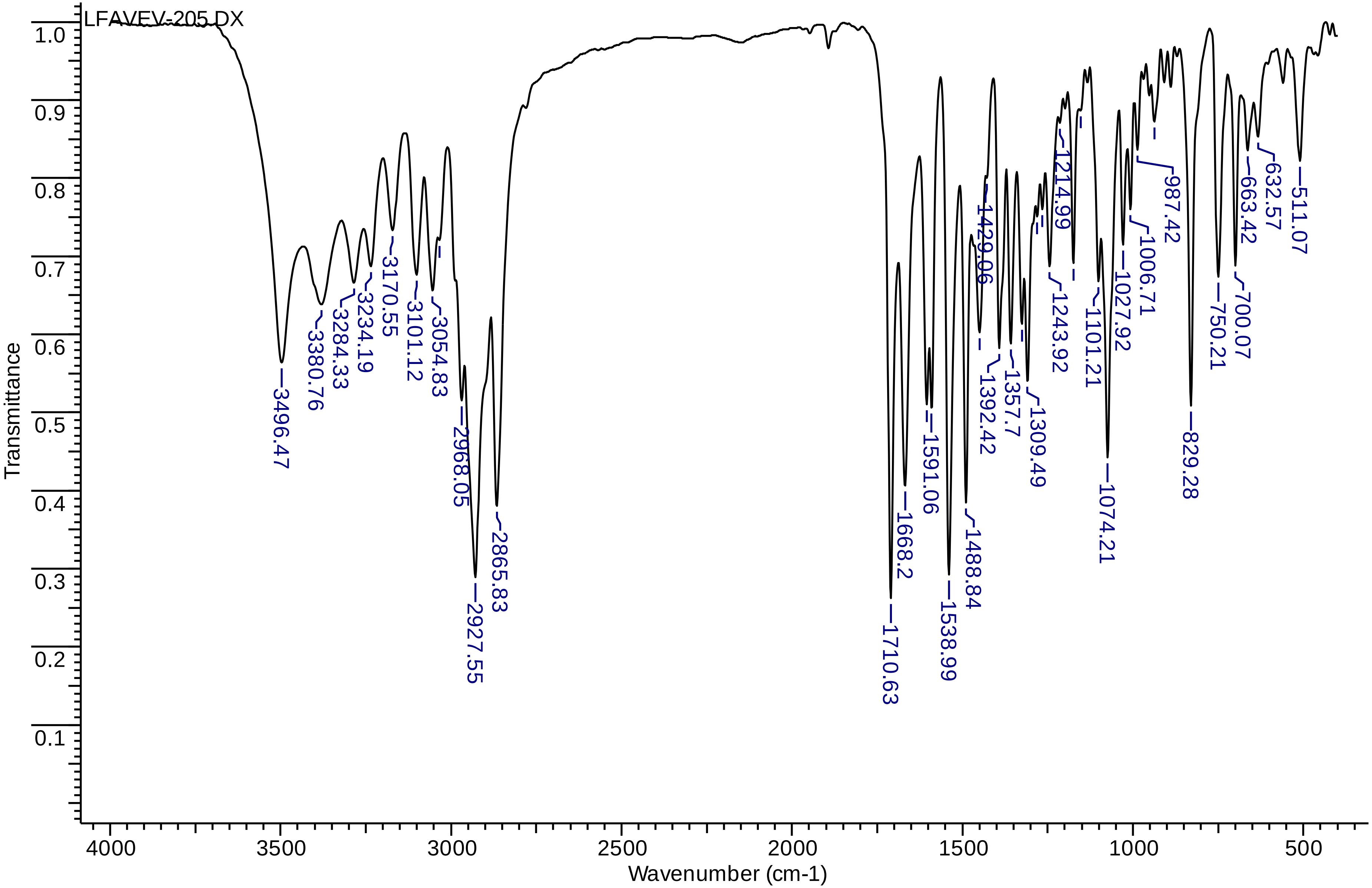

IR, ν, cm^{-1}: 1710 (C=O st); 1668 (C=O st (amide))

Spectrum of Compound **10**, ^{1}H NMR, 500MHz, $CDCl_3$

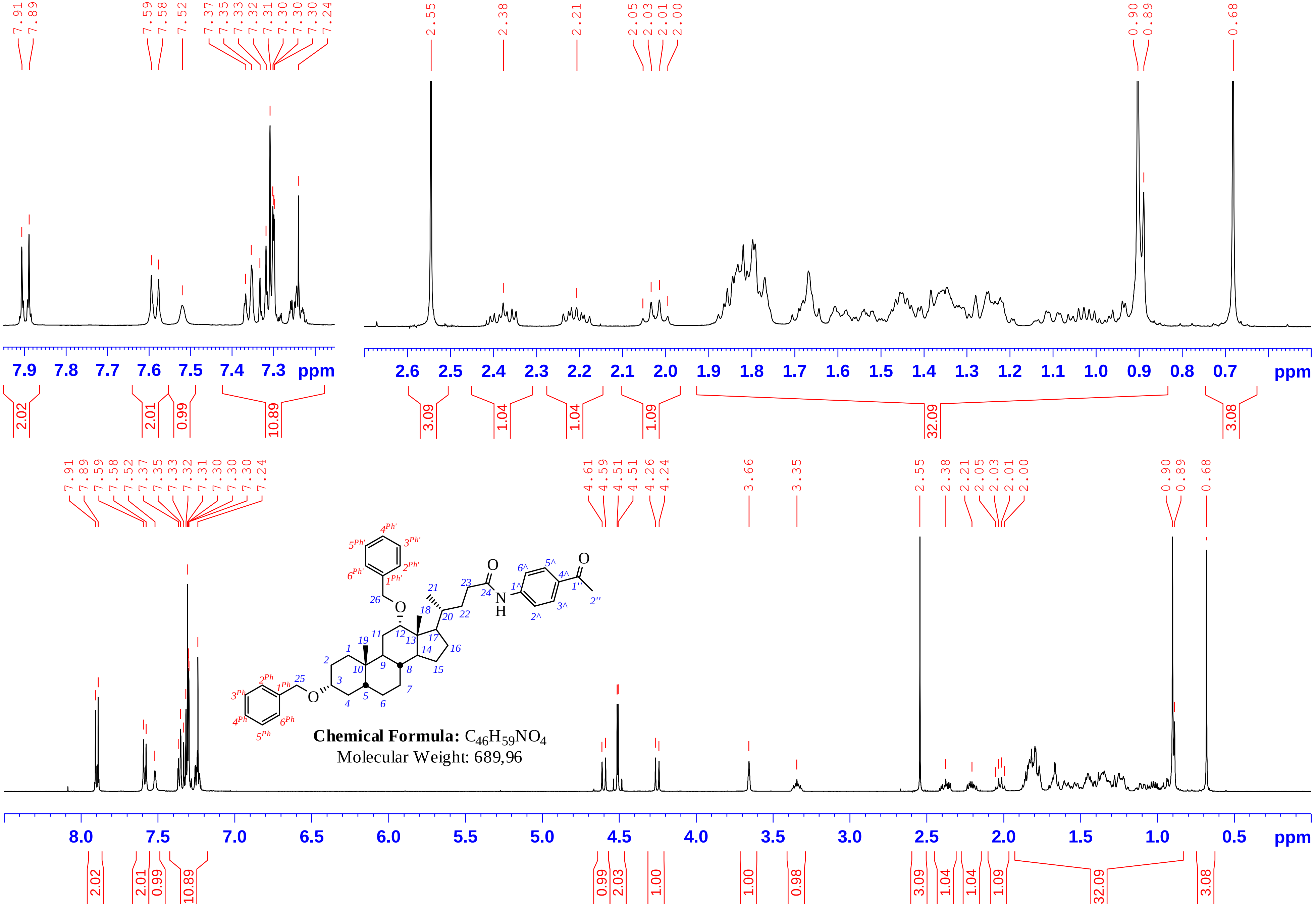

Spectrum of Compound **10**, ^{13}C NMR, JMOD, 125MHz, $CDCl_3$

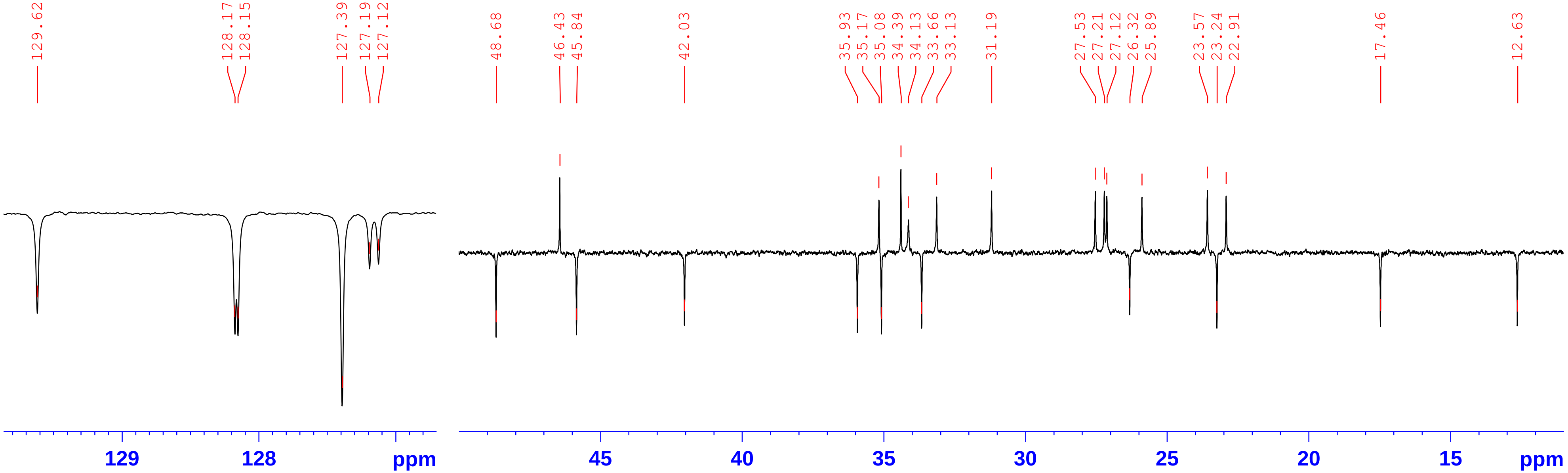

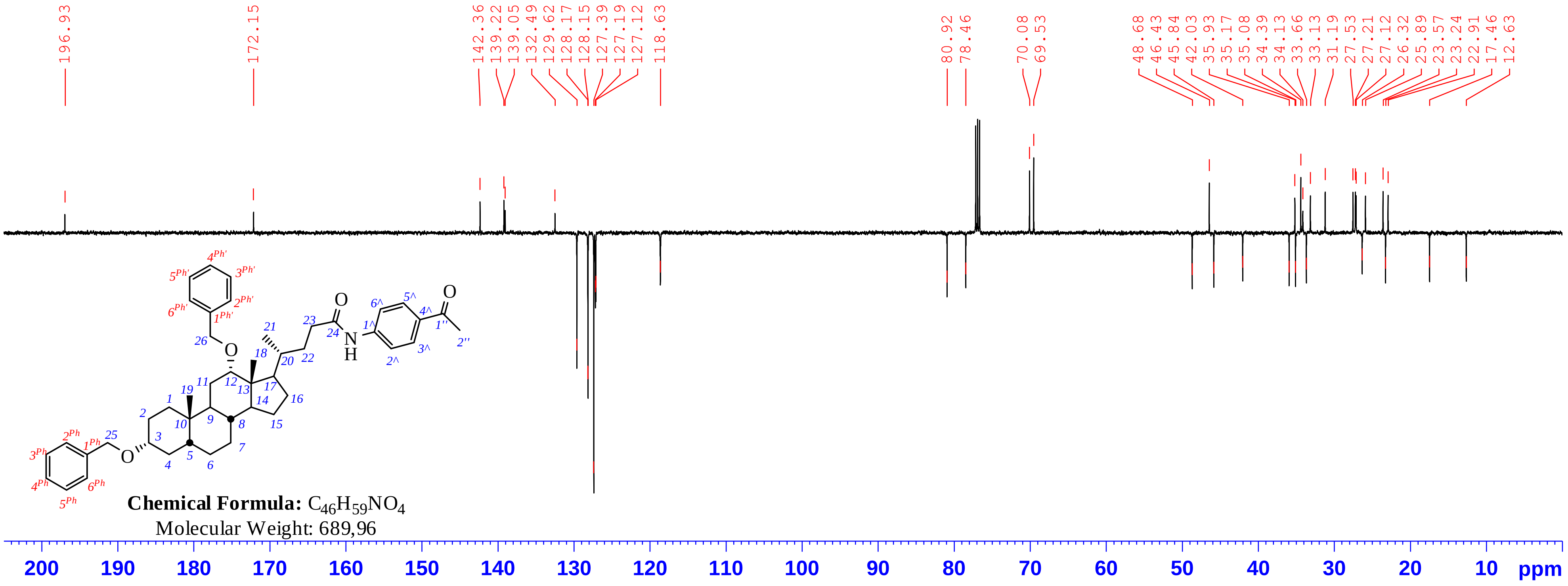

Chemical Formula: $C_{46}H_{59}NO_4$

Molecular Weight: 689,96

High resolution mass spectrum of compound **10**, Tsource=100°C, Tprobe=160°C

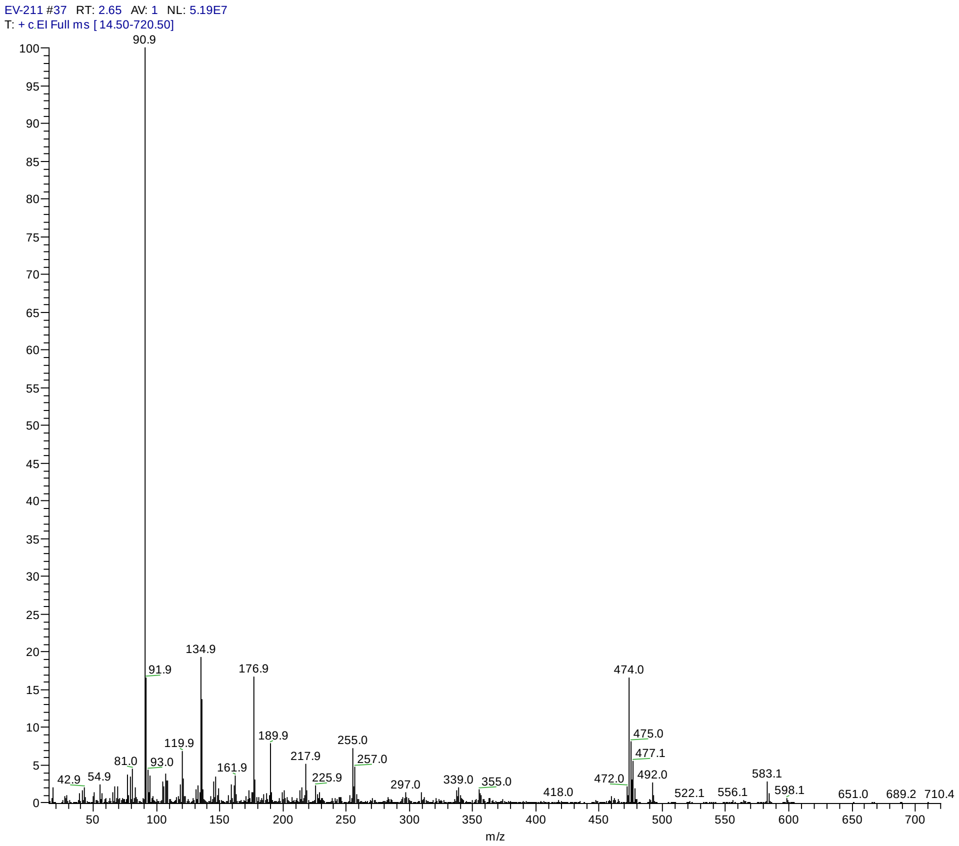

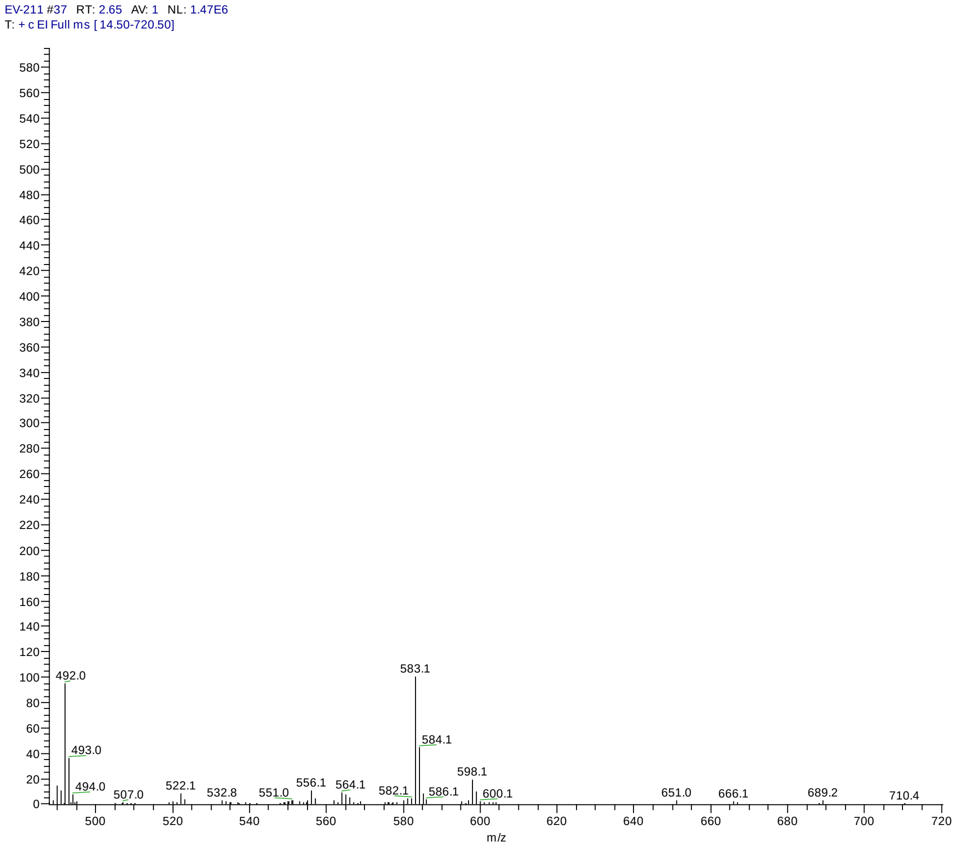

Calculated	m/z=689.4439 $(C_{46}H_{59}O_4N_1)^+$	$[M]^+$
Found	m/z= 598.3889	
Calculated	m/z=598.3891 $(C_{39}H_{52}O_4N_1)^+$	$[M–PhCH_2]^+$

Spectrum of Compound **10**, ^{1}H NMR, 600MHz, $CDCl_3$

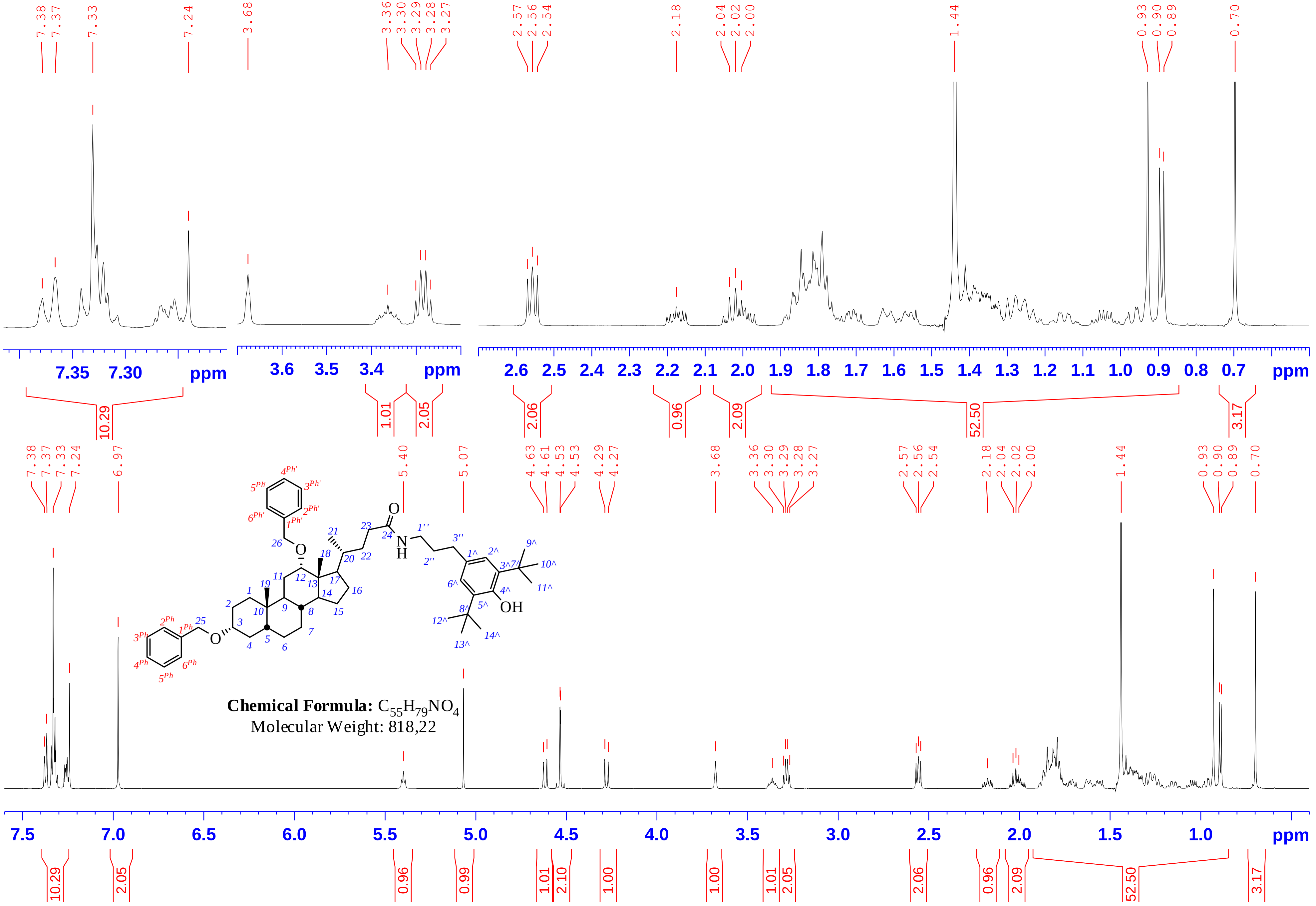

Spectrum of Compound **10**, ^{13}C NMR, JMOD, 150MHz, $CDCl_3$

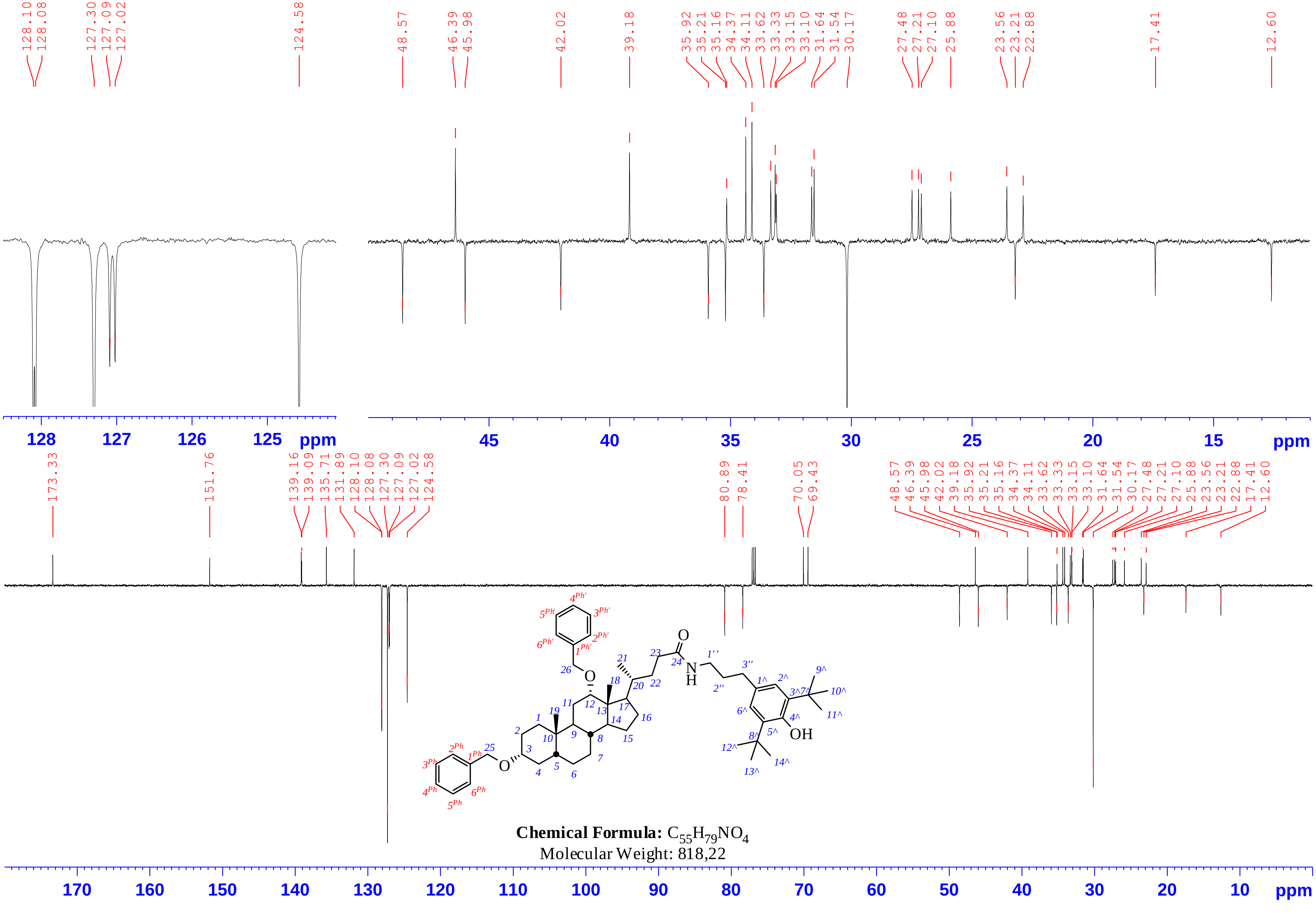

Chemical Formula: $C_{55}H_{79}NO_4$

Molecular Weight: 818,22

High resolution mass spectrum of compound **10**, Tsource=100°C, Tprobe=345°C

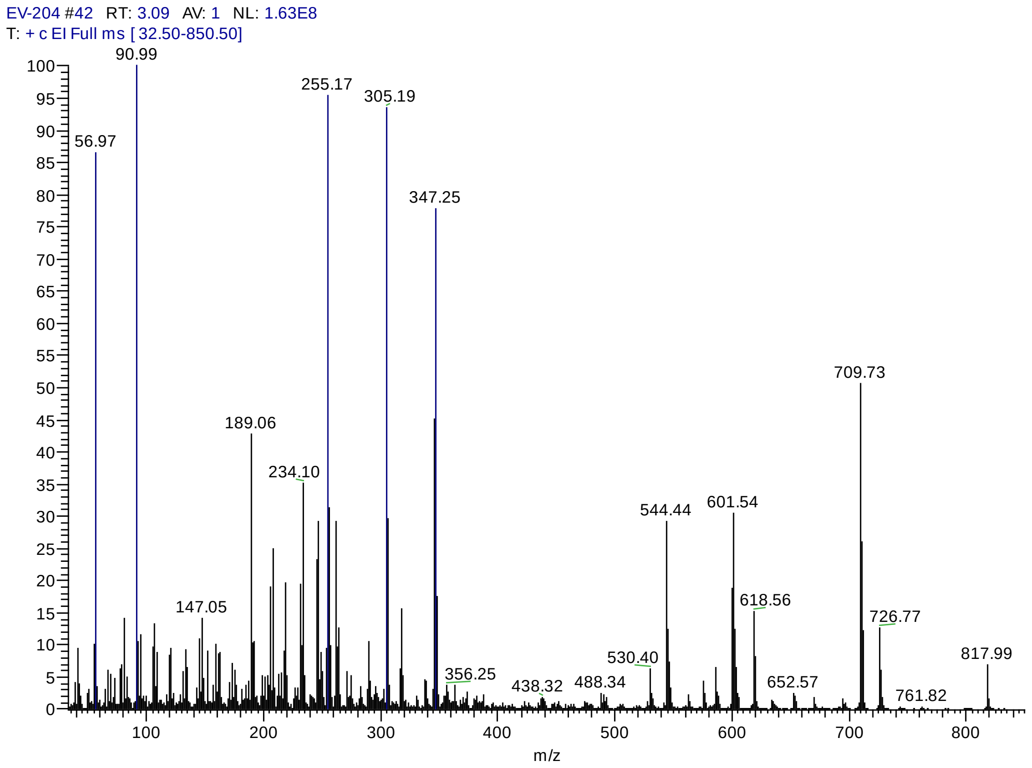

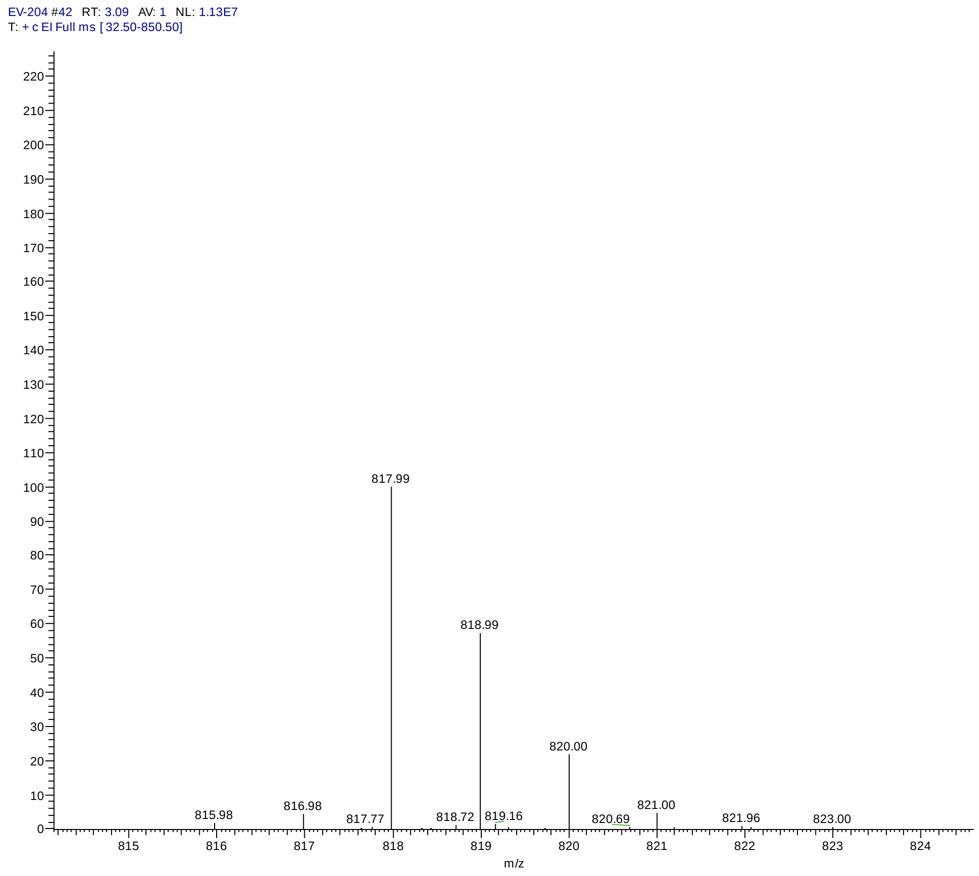

Calculated m/z= 817.6004 $(C_{55}H_{79}O_4N_1)^+$

Found m/z= 817.5991

Spectrum of Compound **11**, ^{1}H NMR, 400MHz, $CDCl_3$

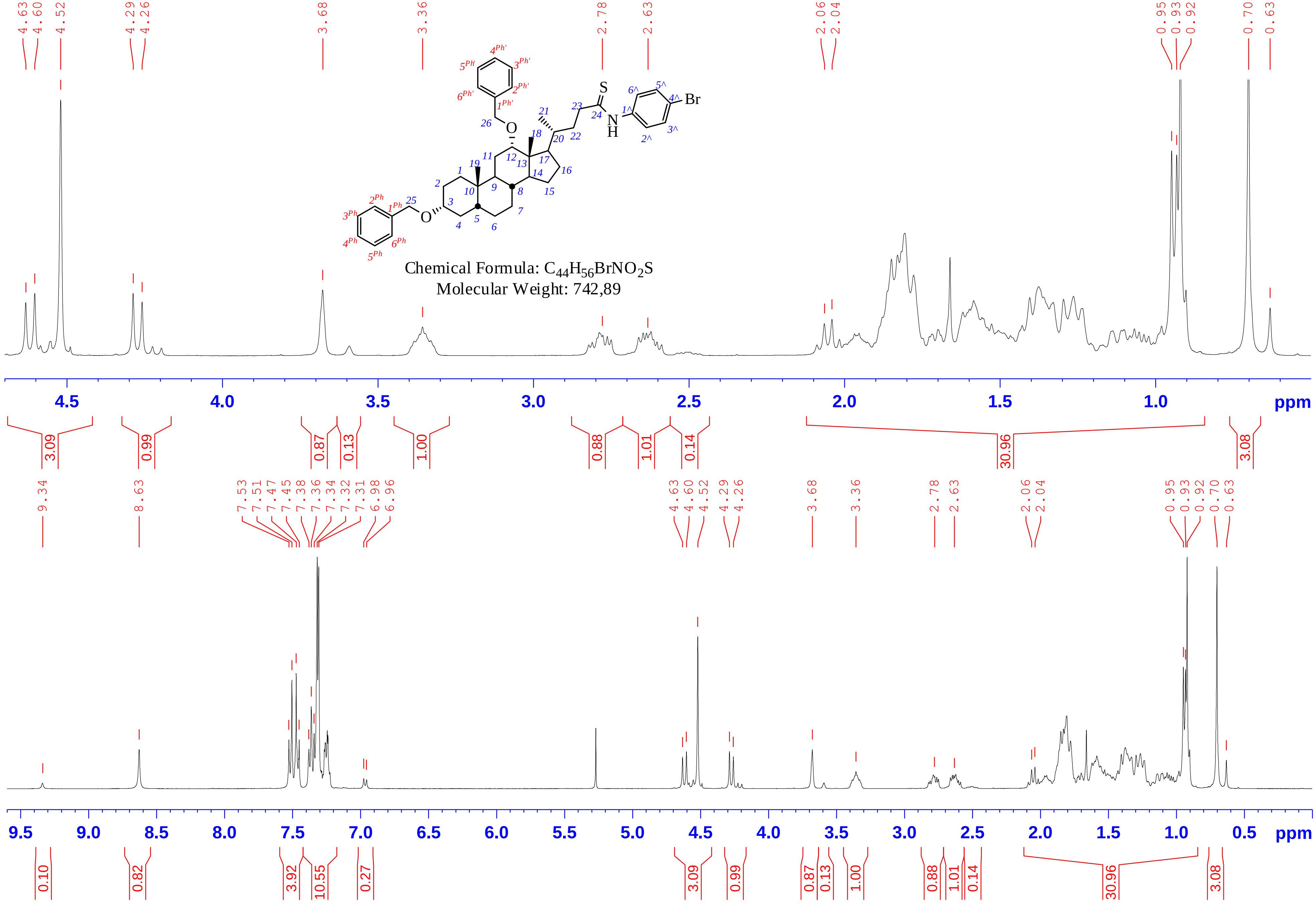

Spectrum of Compound **11**, ^{13}C NMR, JMOD, 75MHz, $CDCl_3$

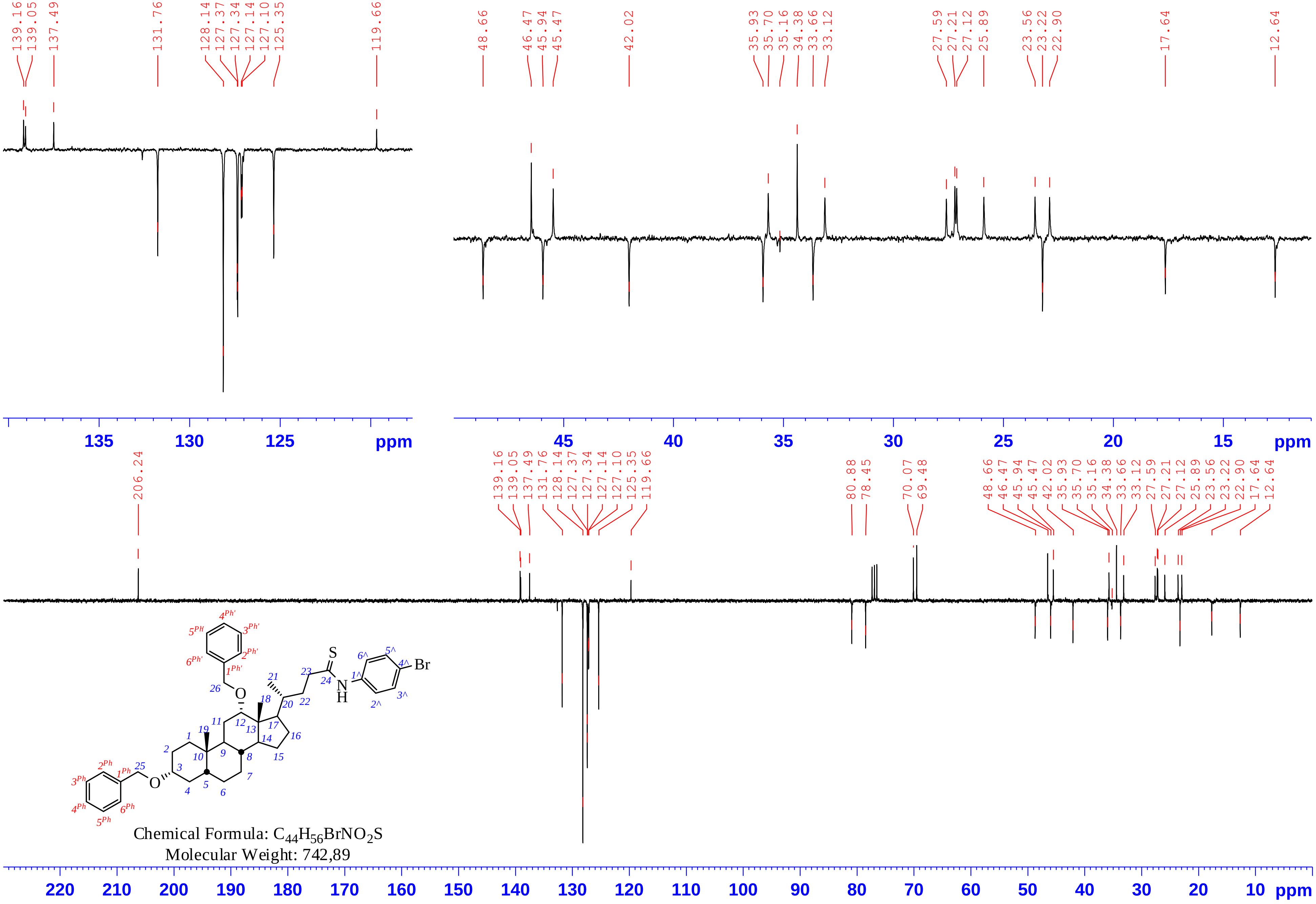

High resolution mass spectrum of compound **11**, Tsource=100°C, Tprobe=300°C

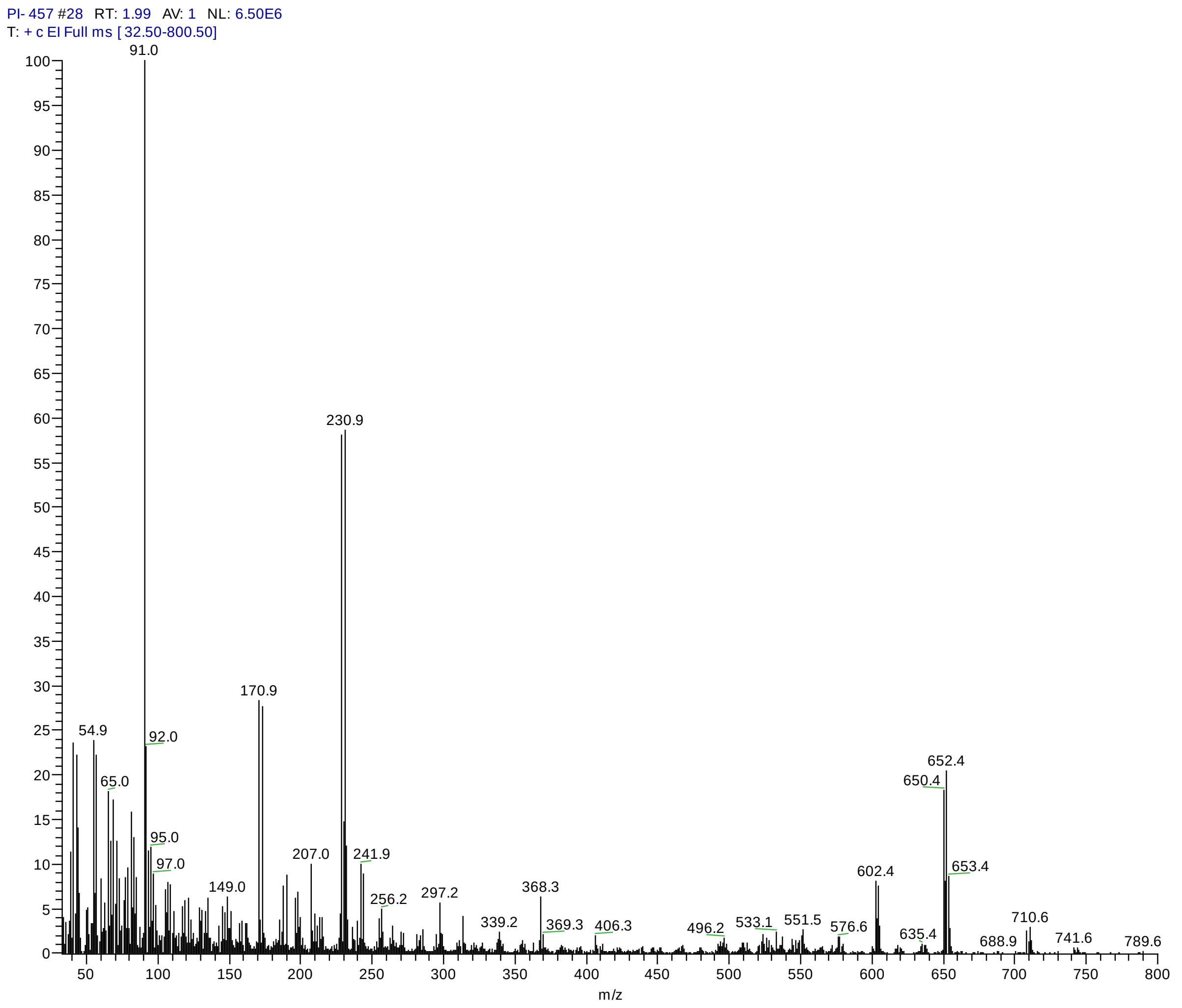

Calculated m/z= 741.3210 ($C_{44}H_{56}O_2N_1{}^{79}Br_1S$)$^+$

Found m/z= 741.3207

IR spectrum of compound **11**

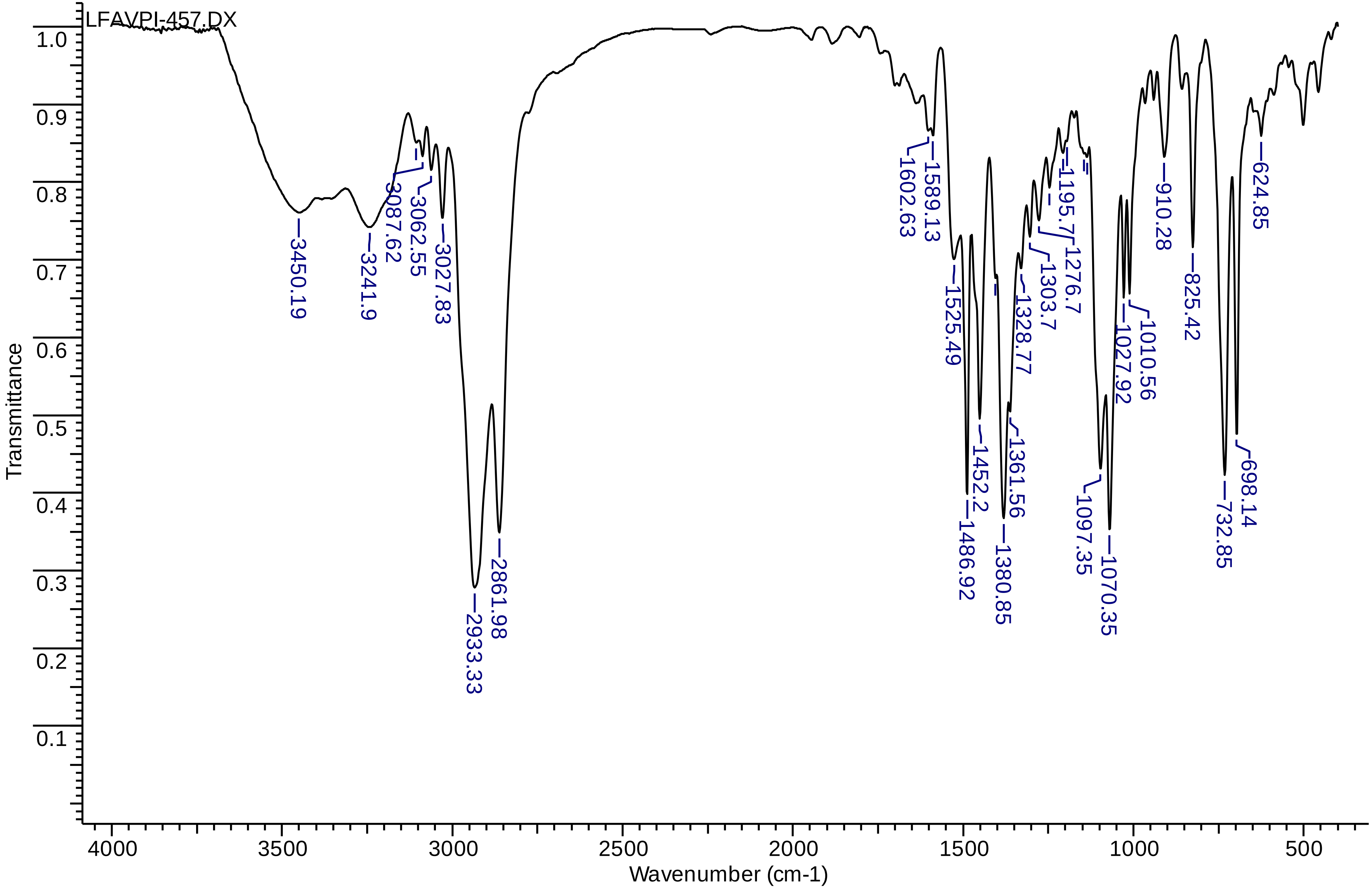

Spectrum of Compound **12**, ^{1}H NMR, 500MHz, $CDCl_3$

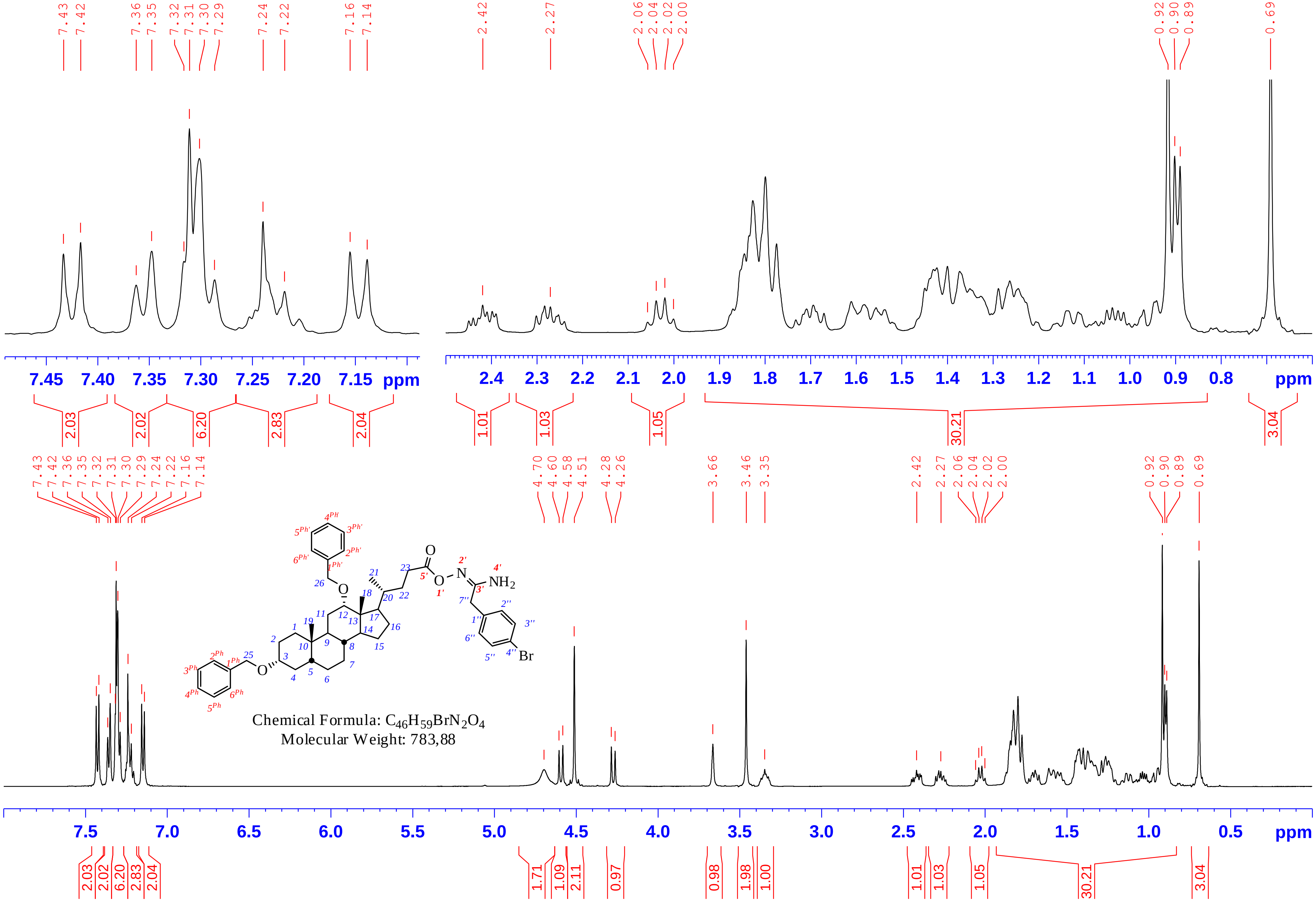

Chemical Formula: $C_{46}H_{59}BrN_2O_4$

Molecular Weight: 783,88

Spectrum of Compound **12**, ^{13}C NMR, JMOD, 125MHz, $CDCl_3$

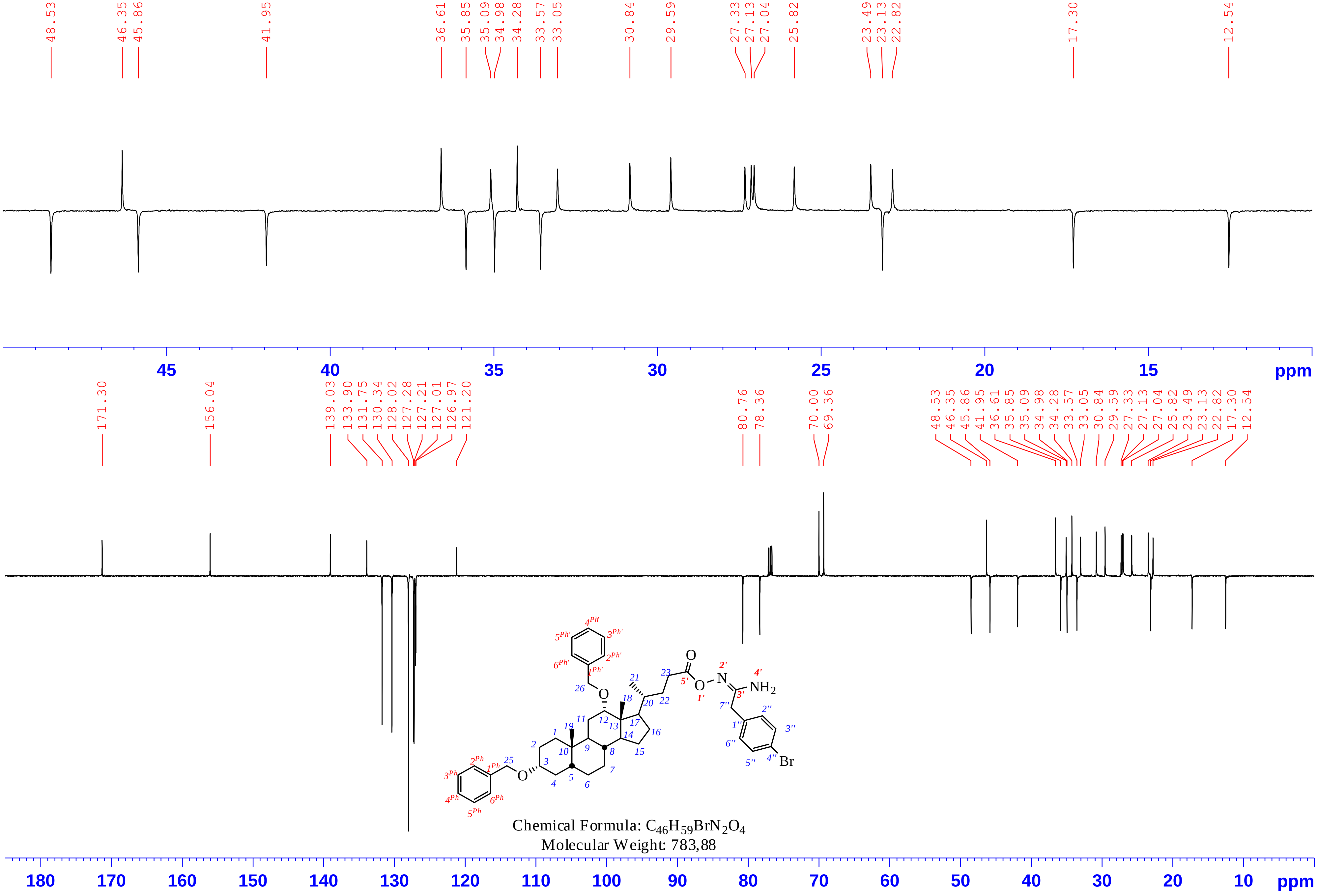

High resolution mass spectrum of compound **12**, Tsource=115°C, Tprobe=280°C

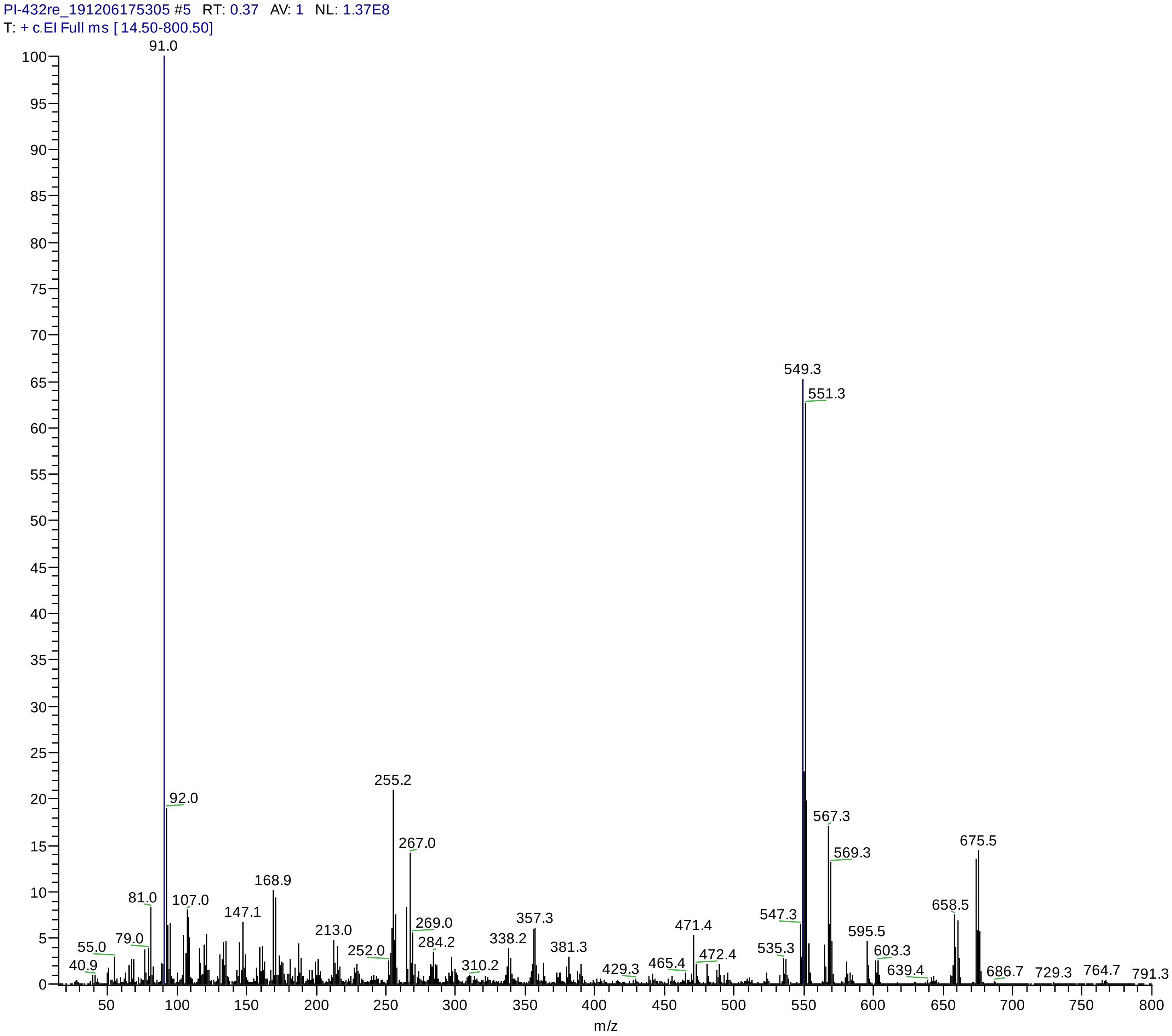

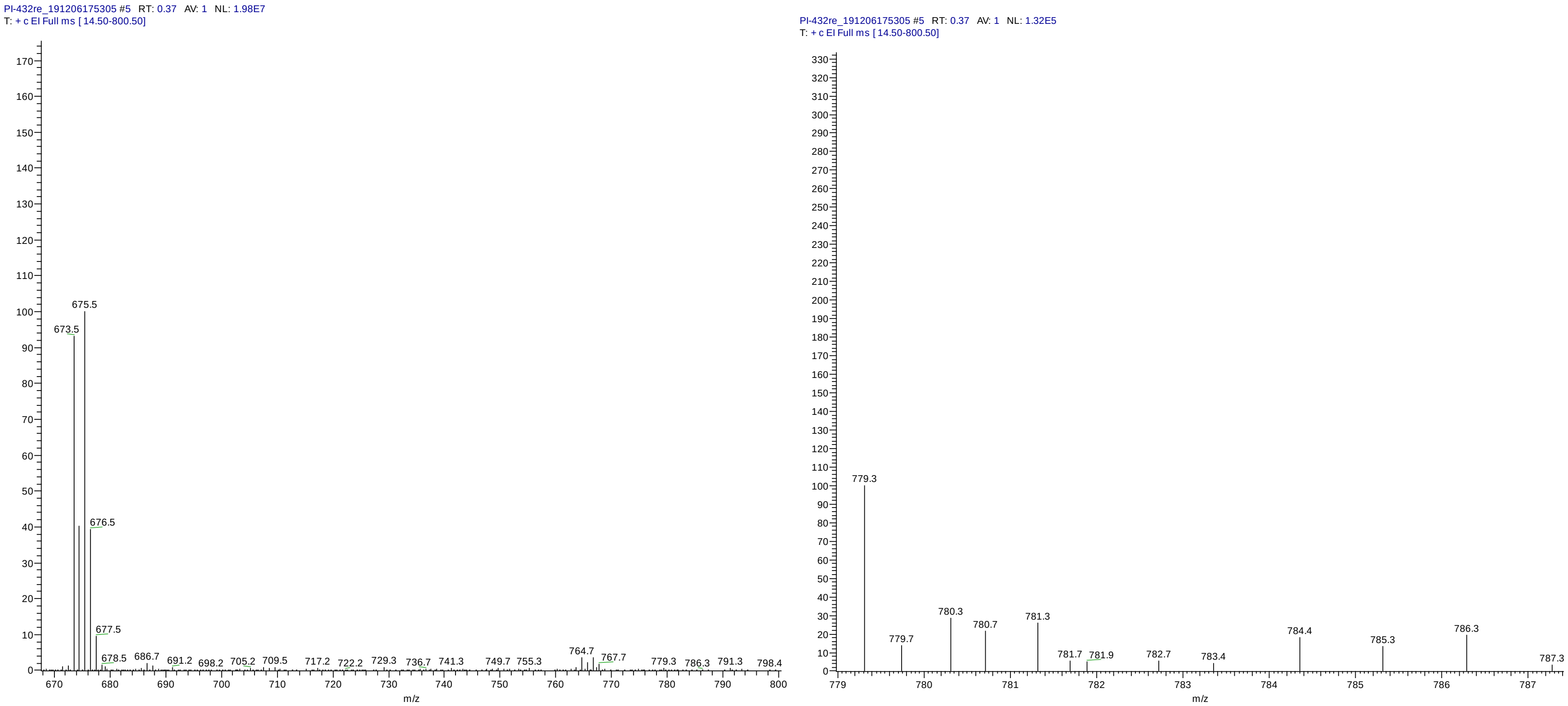

Calculated	m/z=782.3653 ($C_{46}H_{59}O_4N_2{}^{79}Br_1)^+$	$[M]^+$
Found	m/z=764.3528	
Calculated	m/z=764.3547 ($C_{46}H_{57}O_3N_2{}^{79}Br_1)^+$	$[M–H_2O]^+$

Spectrum of Compound **13**, ^{1}H NMR, 500MHz, $CDCl_3$

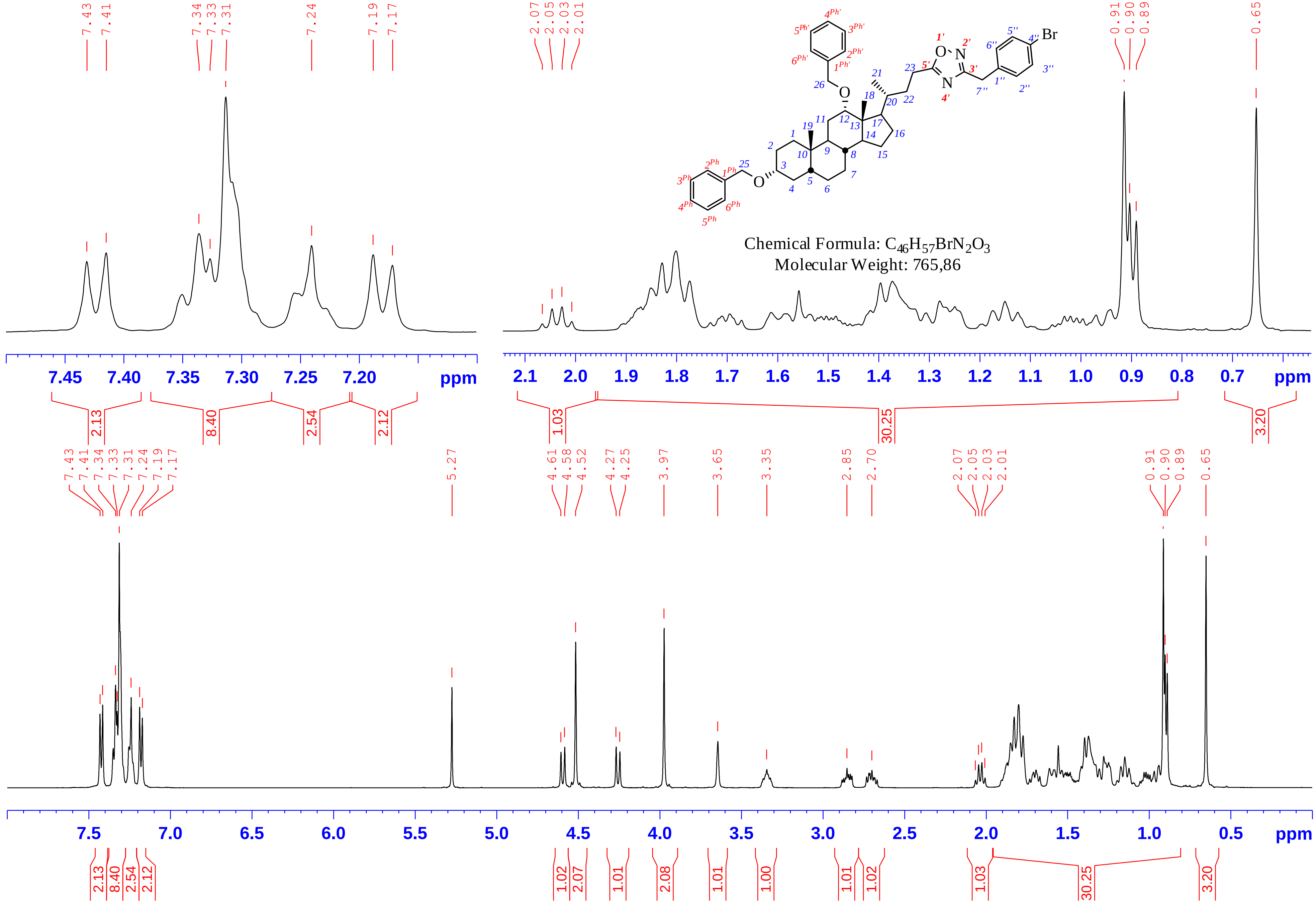

Spectrum of Compound **13**, ^{13}C NMR, JMOD, 125MHz, $CDCl_3$

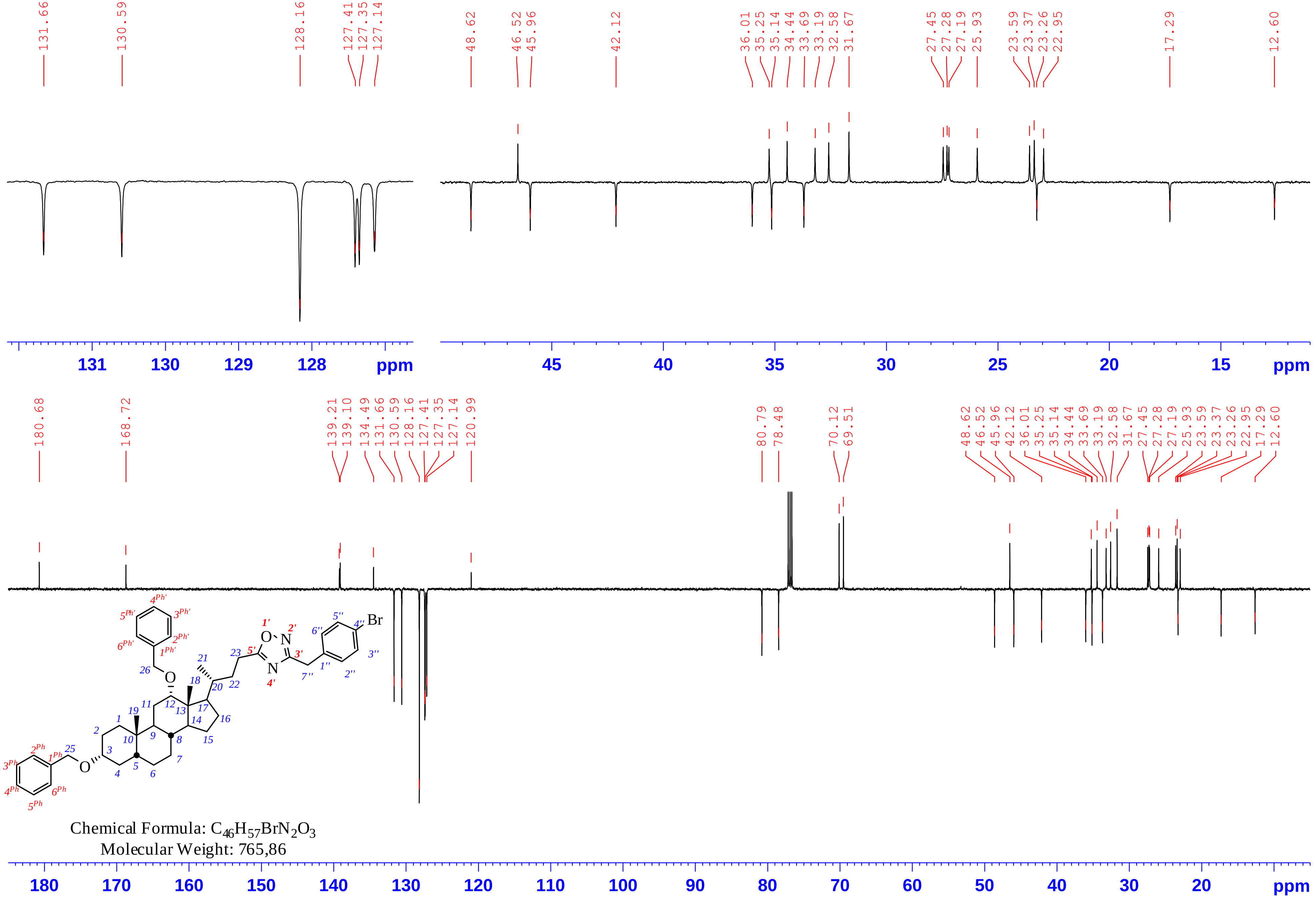

High resolution mass spectrum of compound **13**, Tsource=100°C, Tprobe=325°C

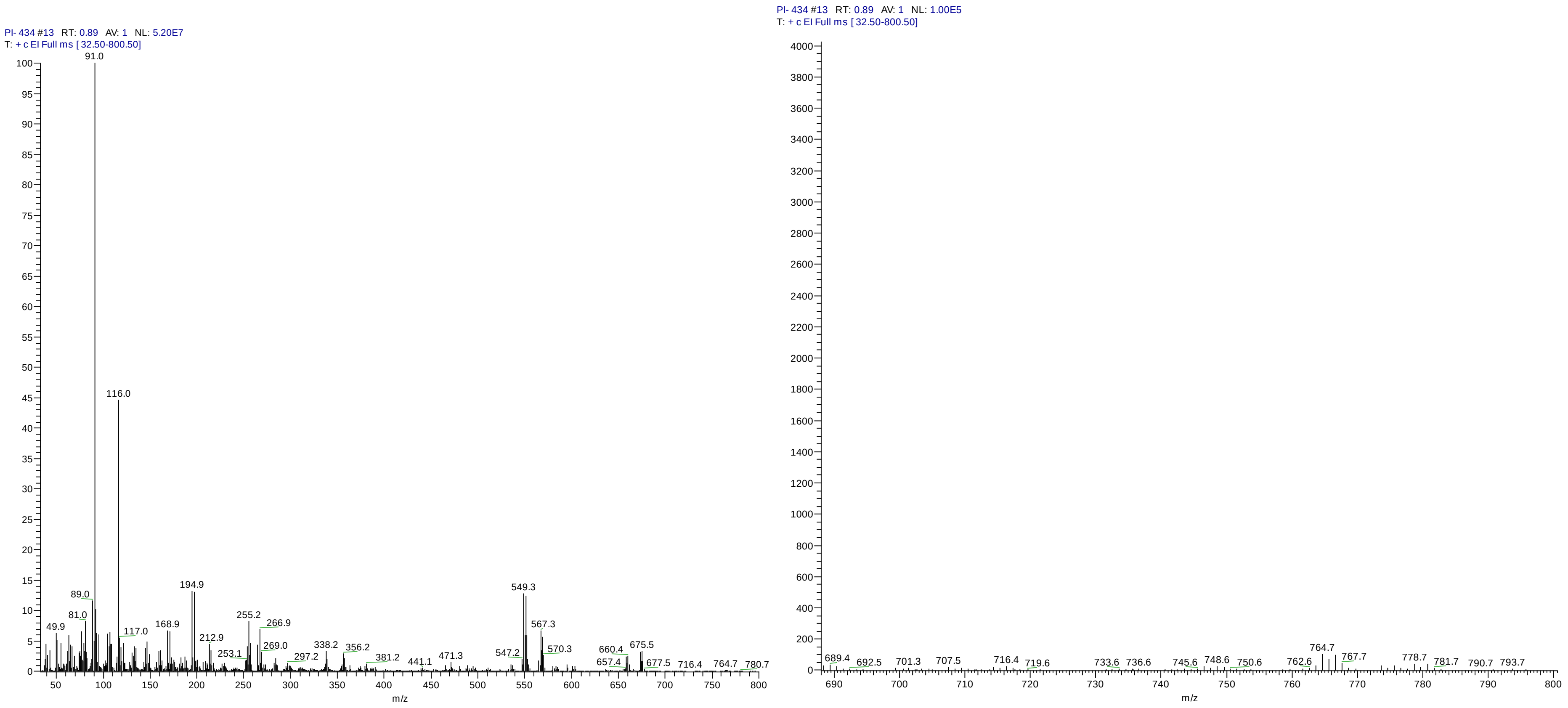

Calculated	m/z=764.3547 $(C_{46}H_{57}O_3N_2{}^{79}Br_1)^+$	$[M]^+$
Found	m/z=673.3014	
Calculated	m/z=673.3000 $(C_{39}H_{50}O_3N_2{}^{79}Br_1)^+$	$[M–PhCH_2]^+$

Spectrum of Compound **14**, ^{1}H NMR, 500MHz, $CDCl_3$

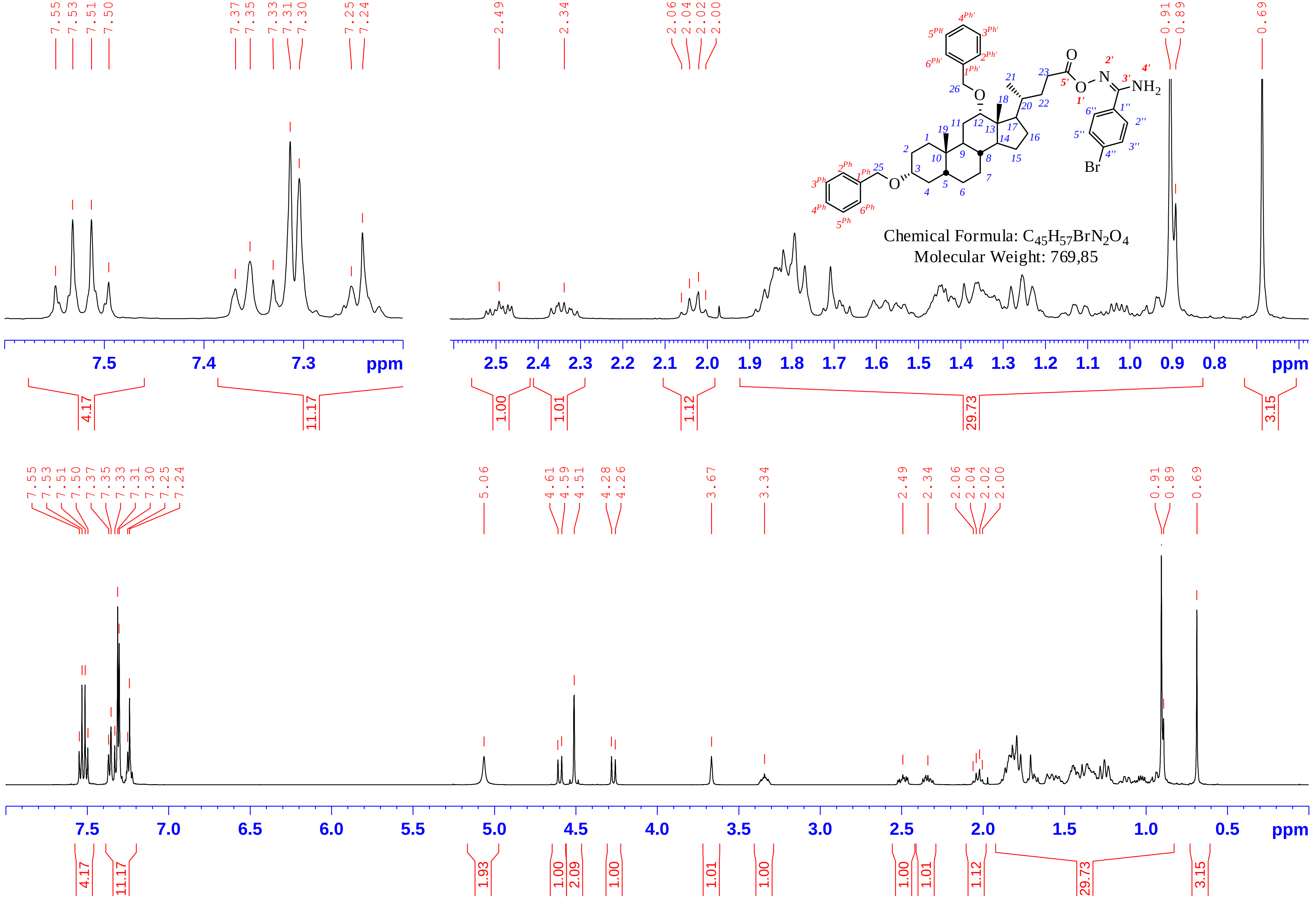

Spectrum of Compound **14**, ^{13}C NMR, JMOD, 125MHz, $CDCl_3$

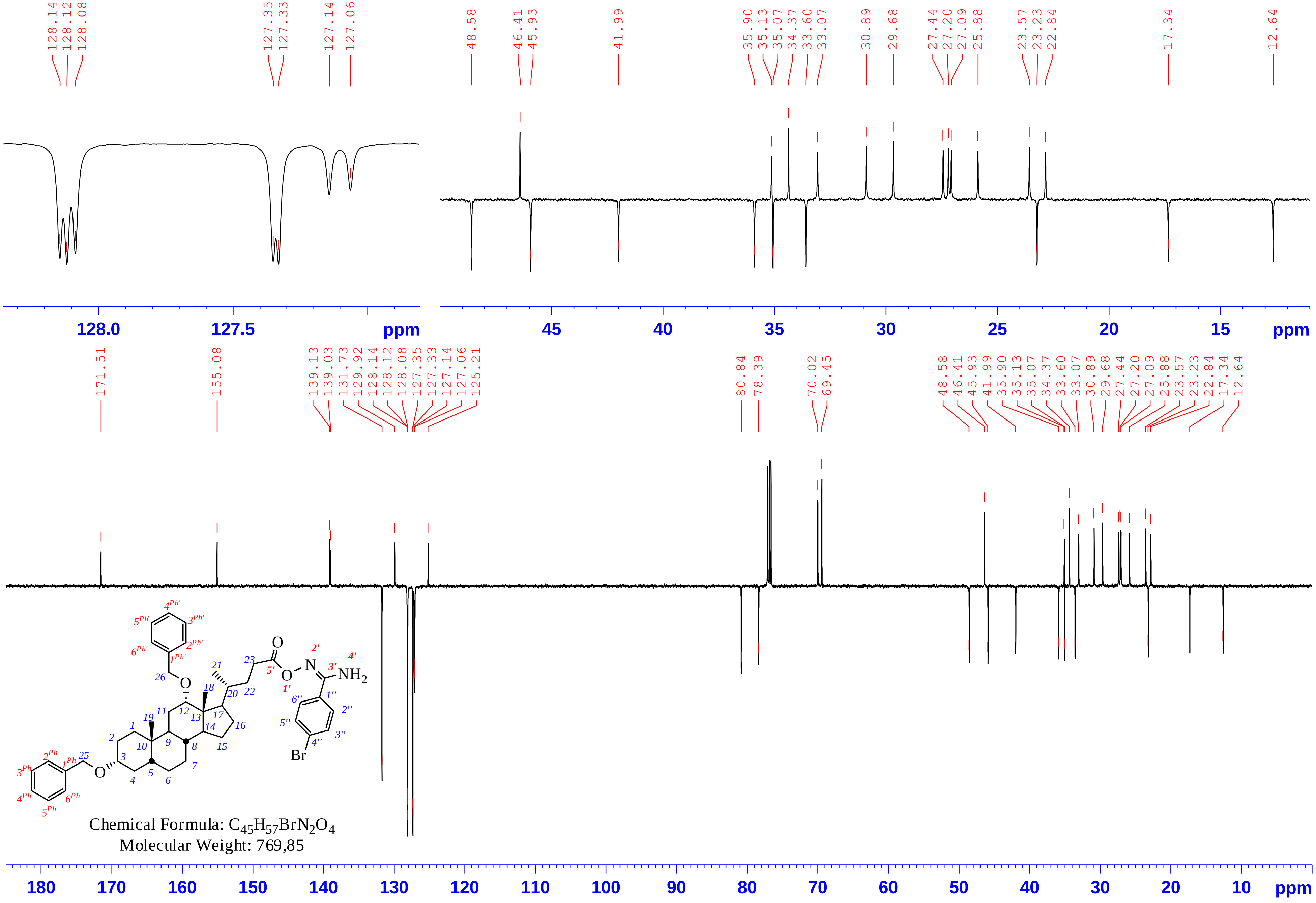

High resolution mass spectrum of compound **14**, Tsource=100°C, Tprobe=340°C

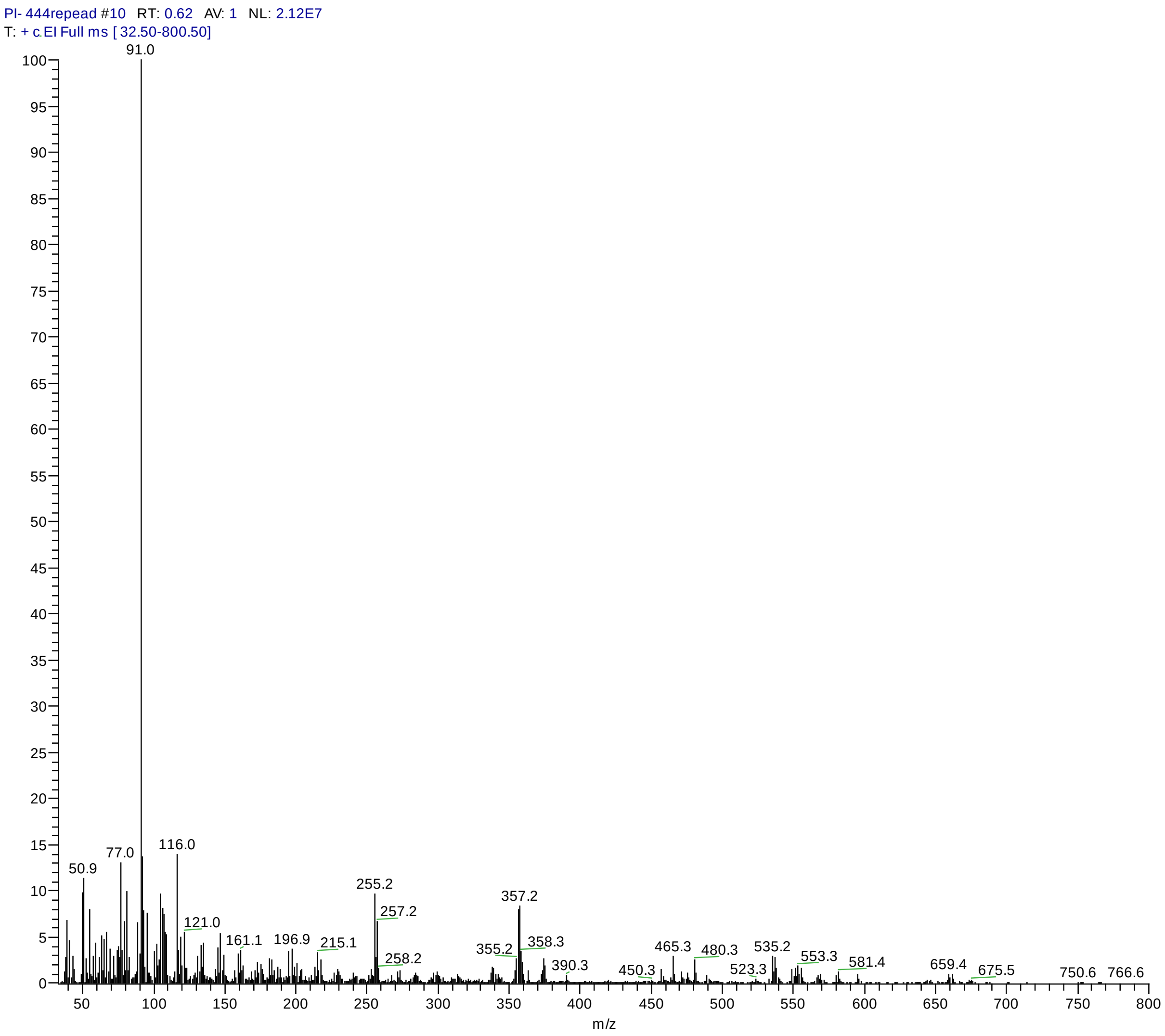

Calculated	m/z=768.3496 ($C_{45}H_{57}O_4N_2{}^{79}Br_1$)$^+$	$[M]^+$
Found	m/z=659.2835	
Calculated	m/z=659.2835 ($C_{38}H_{48}O_3N_2{}^{79}Br_1$)$^+$	$[M–PhCH_2–H_2O]^+$

Spectrum of Compound **15**, ^{1}H NMR, 400MHz, $CDCl_3$

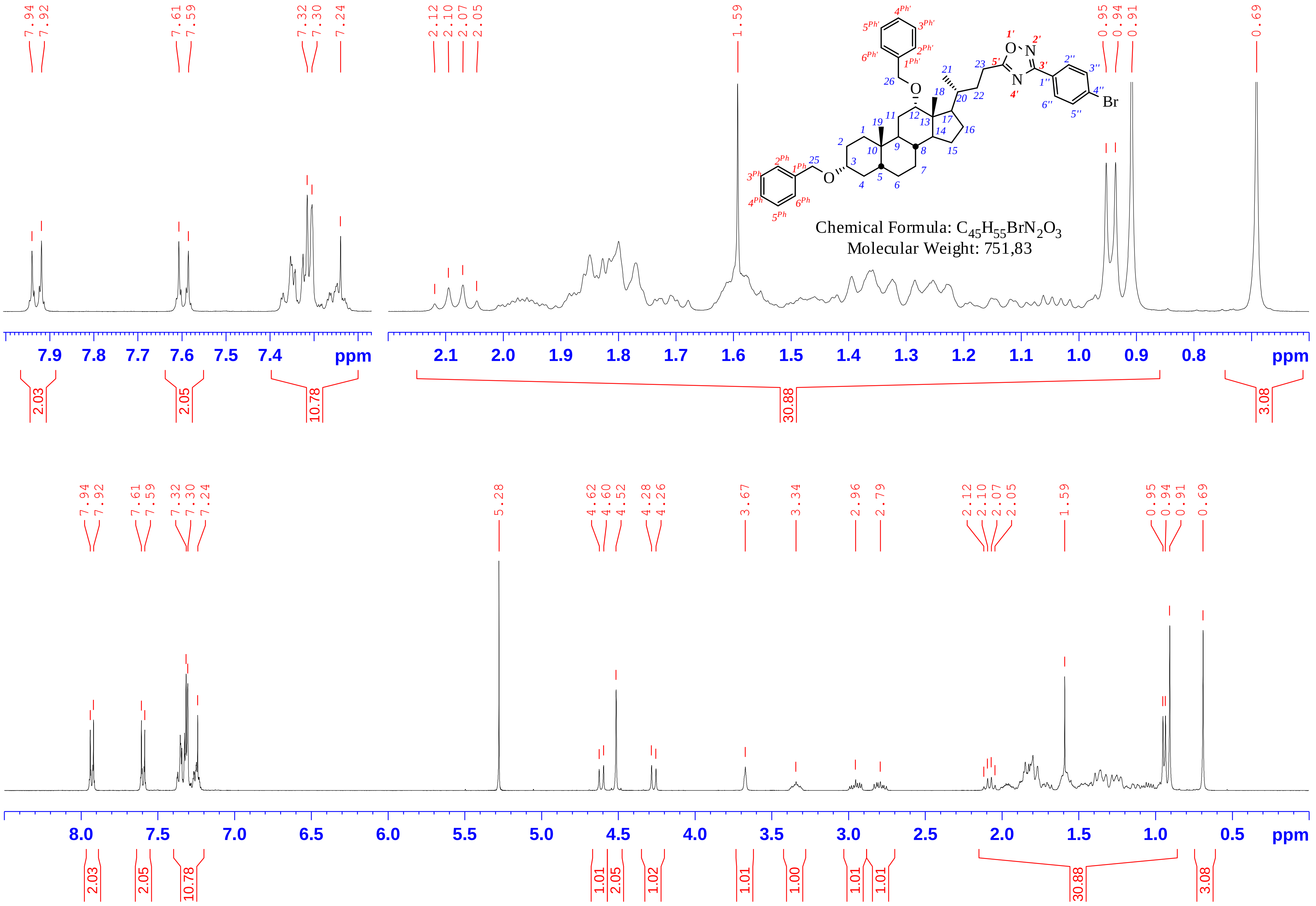

Spectrum of Compound **15**, ^{13}C NMR, JMOD, 100MHz, $CDCl_3$

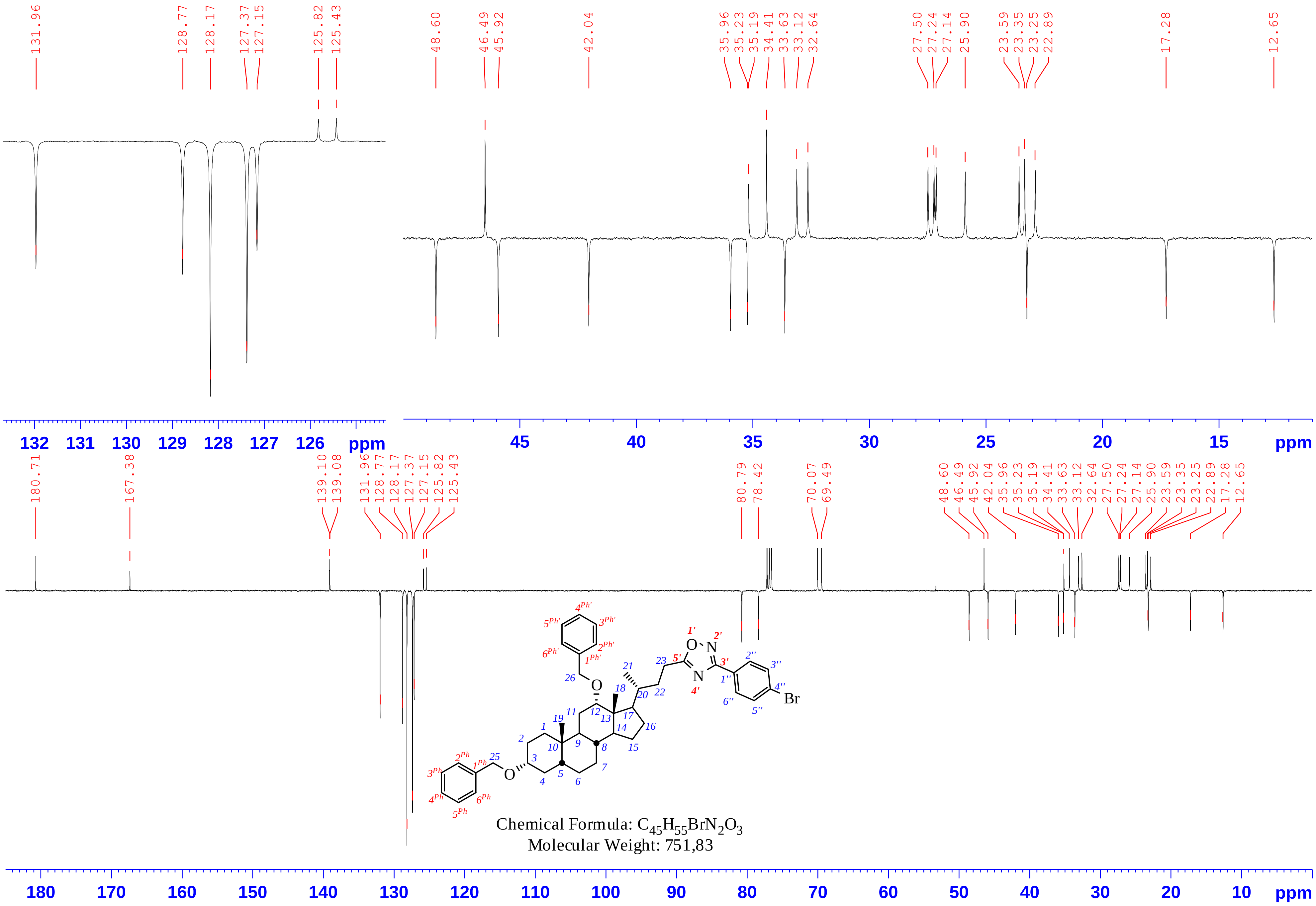

High resolution mass spectrum of compound **15**, Tsource=100°C, Tprobe=340°C

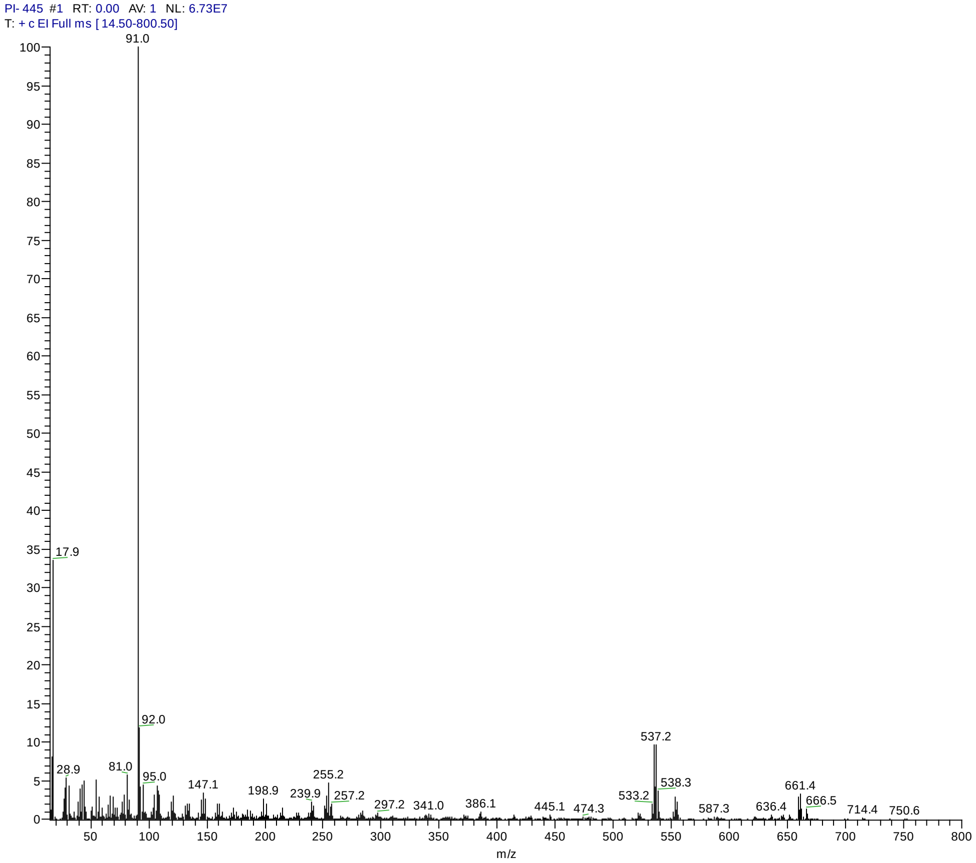

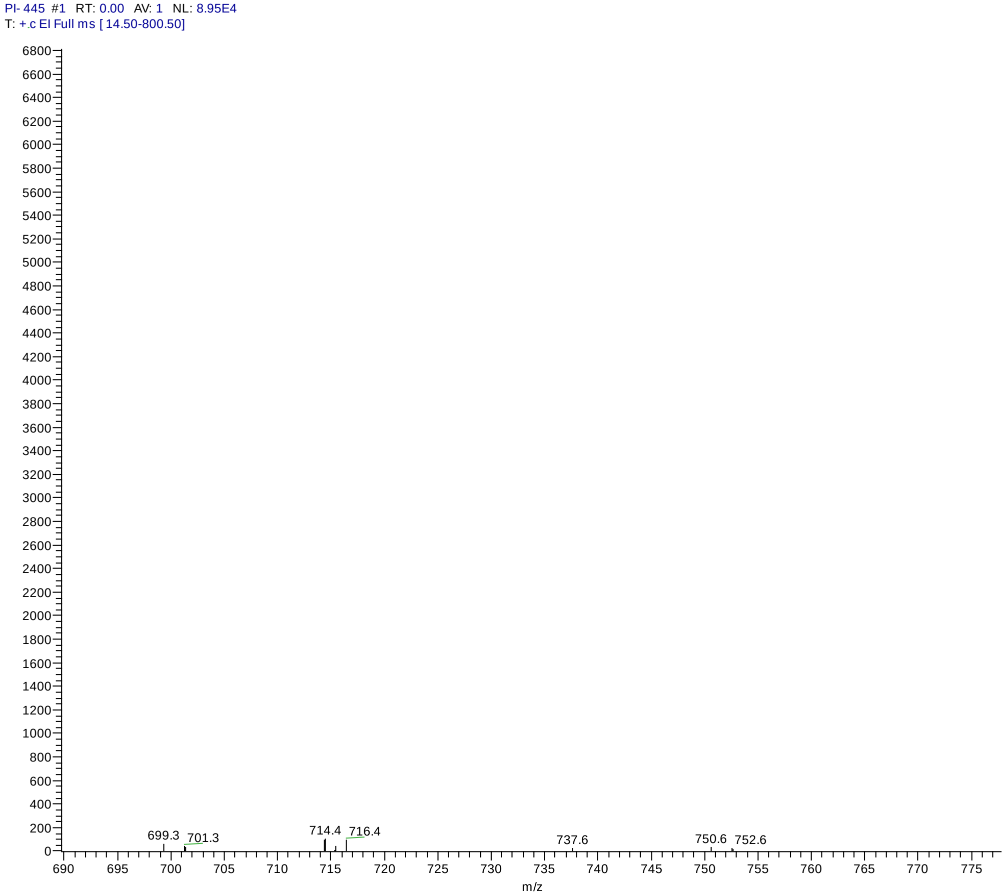

Calculated	m/z=750.3391 ($C_{45}H_{55}O_3N_2{}^{79}Br_1$) $^+$	$[M]^+$
Found	m/z=659.2839	
Calculated	m/z=659.2843 ($C_{38}H_{48}O_3N_2{}^{79}Br_1$) $^+$	$[M\text{–}PhCH_2]^+$

Spectrum of Compound **16**, ^{1}H NMR, 500MHz, $CDCl_3$

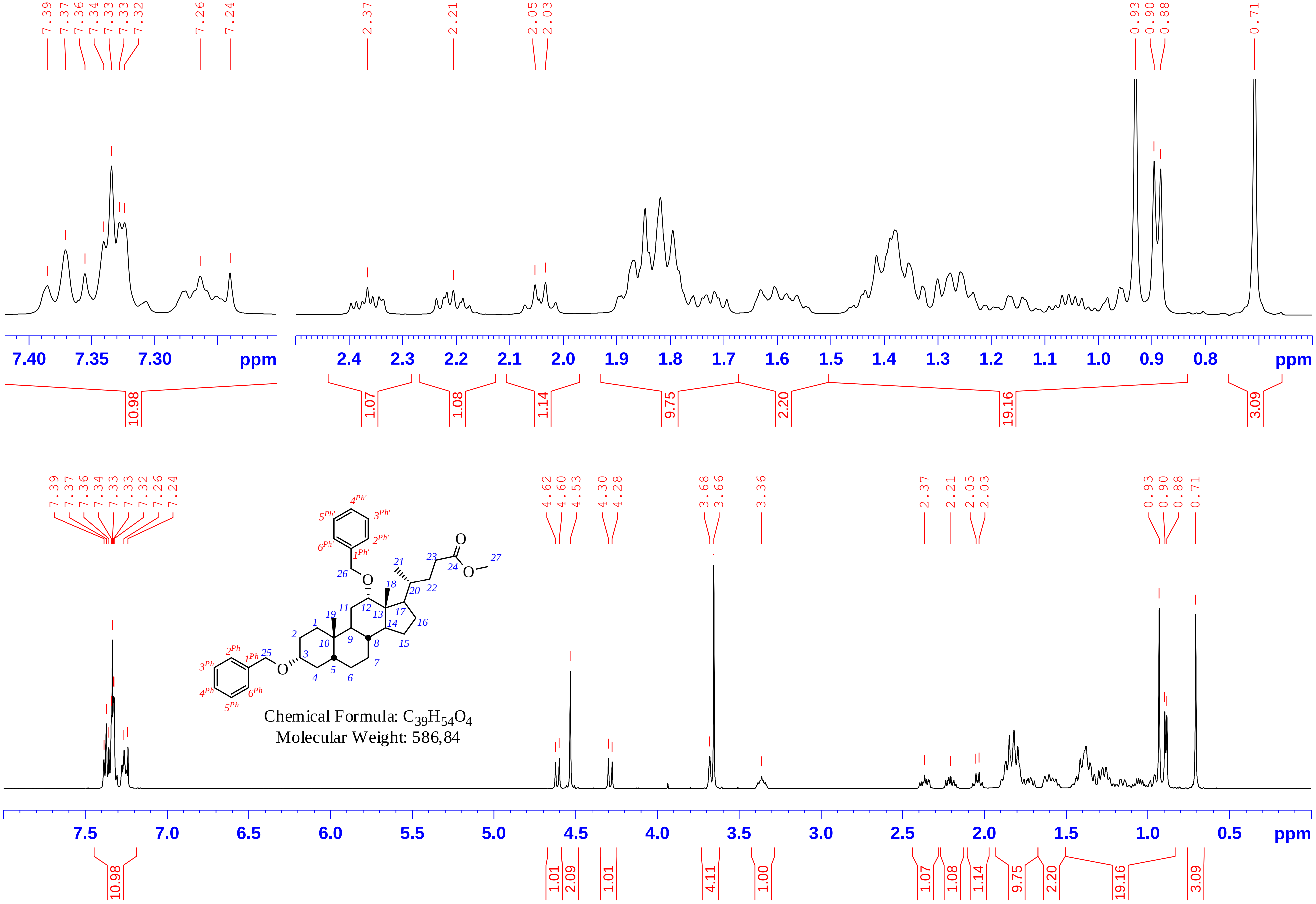

Spectrum of Compound **16**, ^{13}C NMR, JMOD, 125MHz, $CDCl_3$

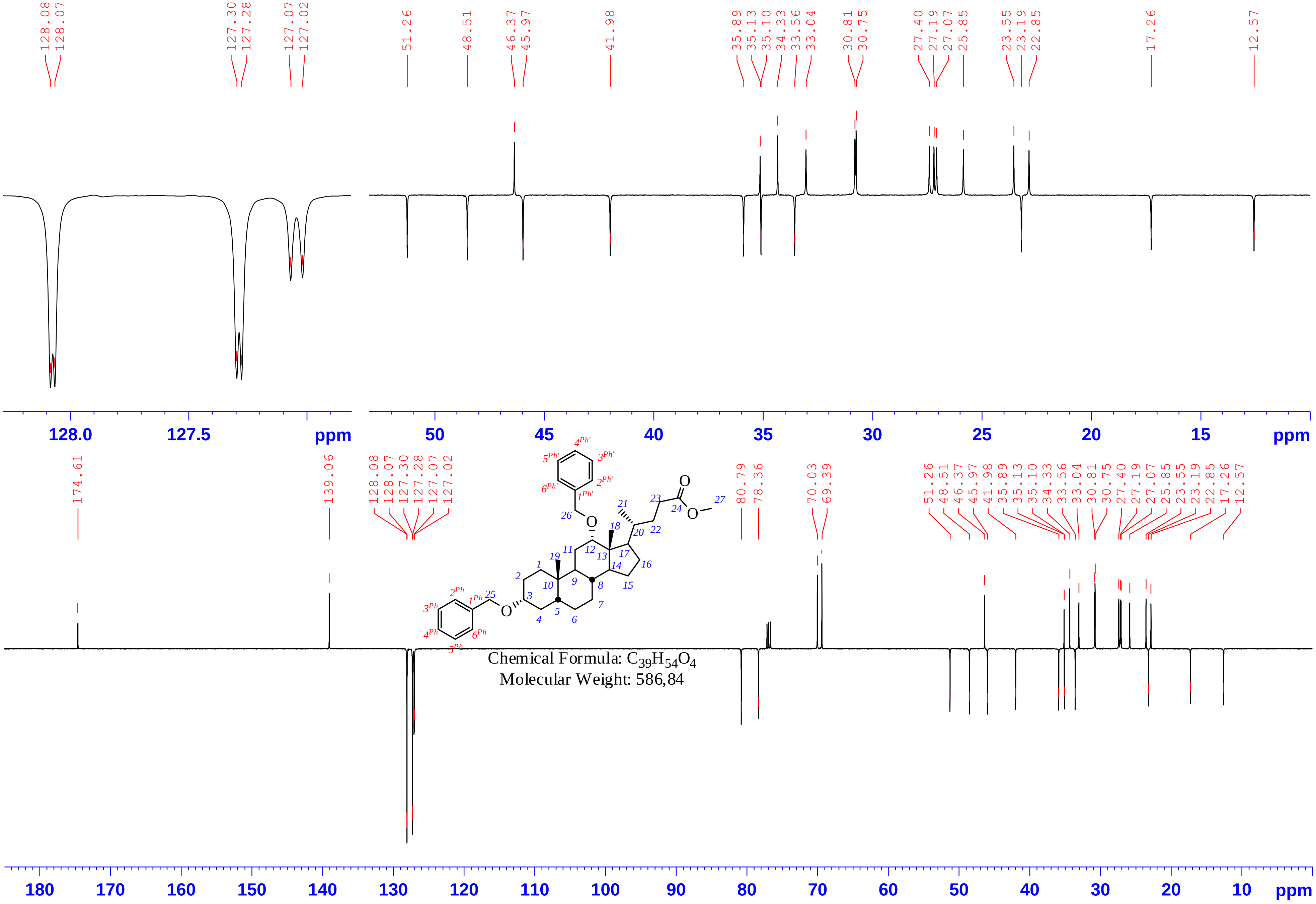

High resolution mass spectrum of compound **16**, Tsource=50°C, Tprobe=330°C

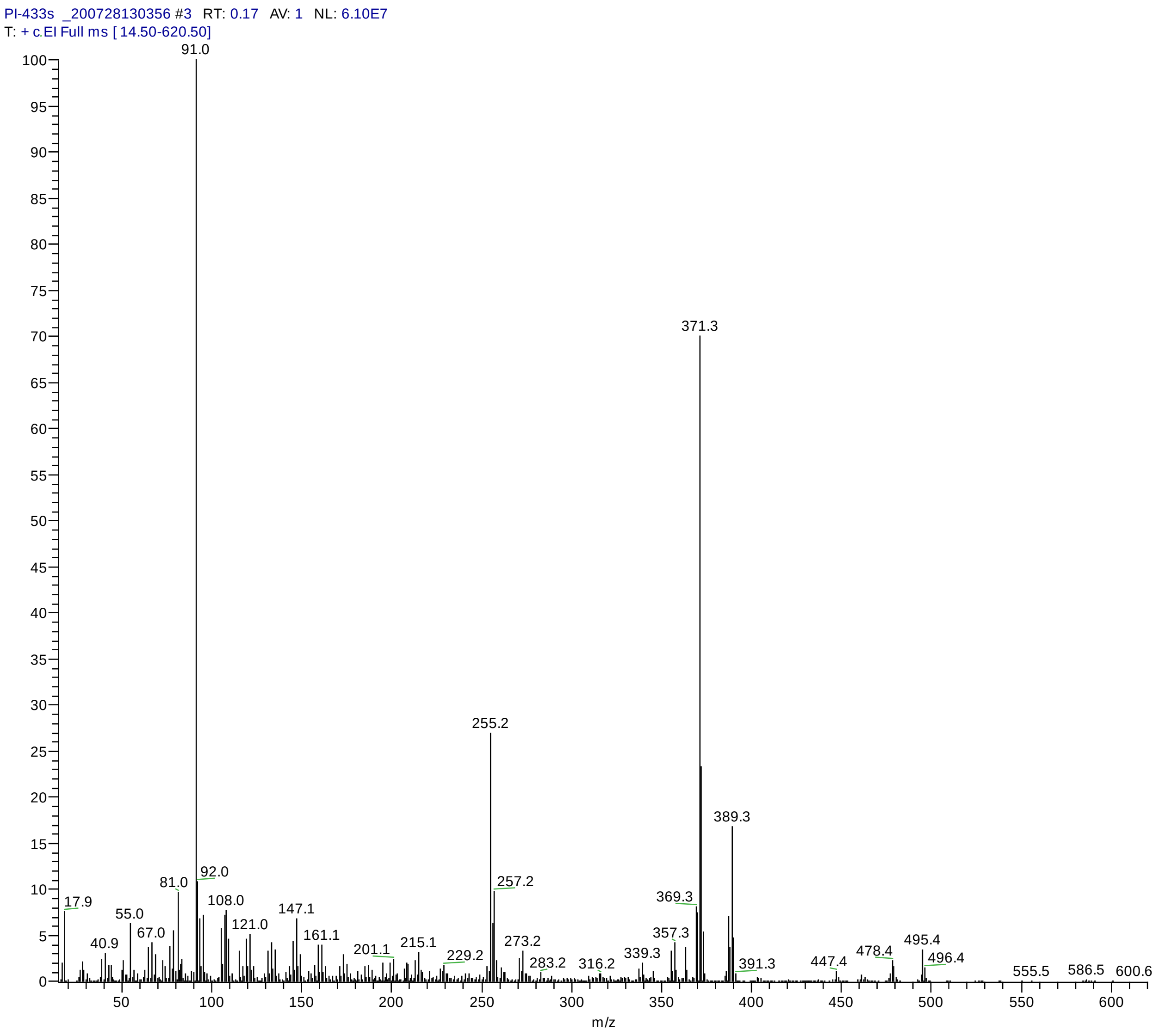

Calculated m/z= 586.4017 ($(C_{39}H_{54}O_4)^+$)

Found m/z= 586.4022

Spectrum of Compound **17**, ^{1}H NMR, 400MHz, $CDCl_3$

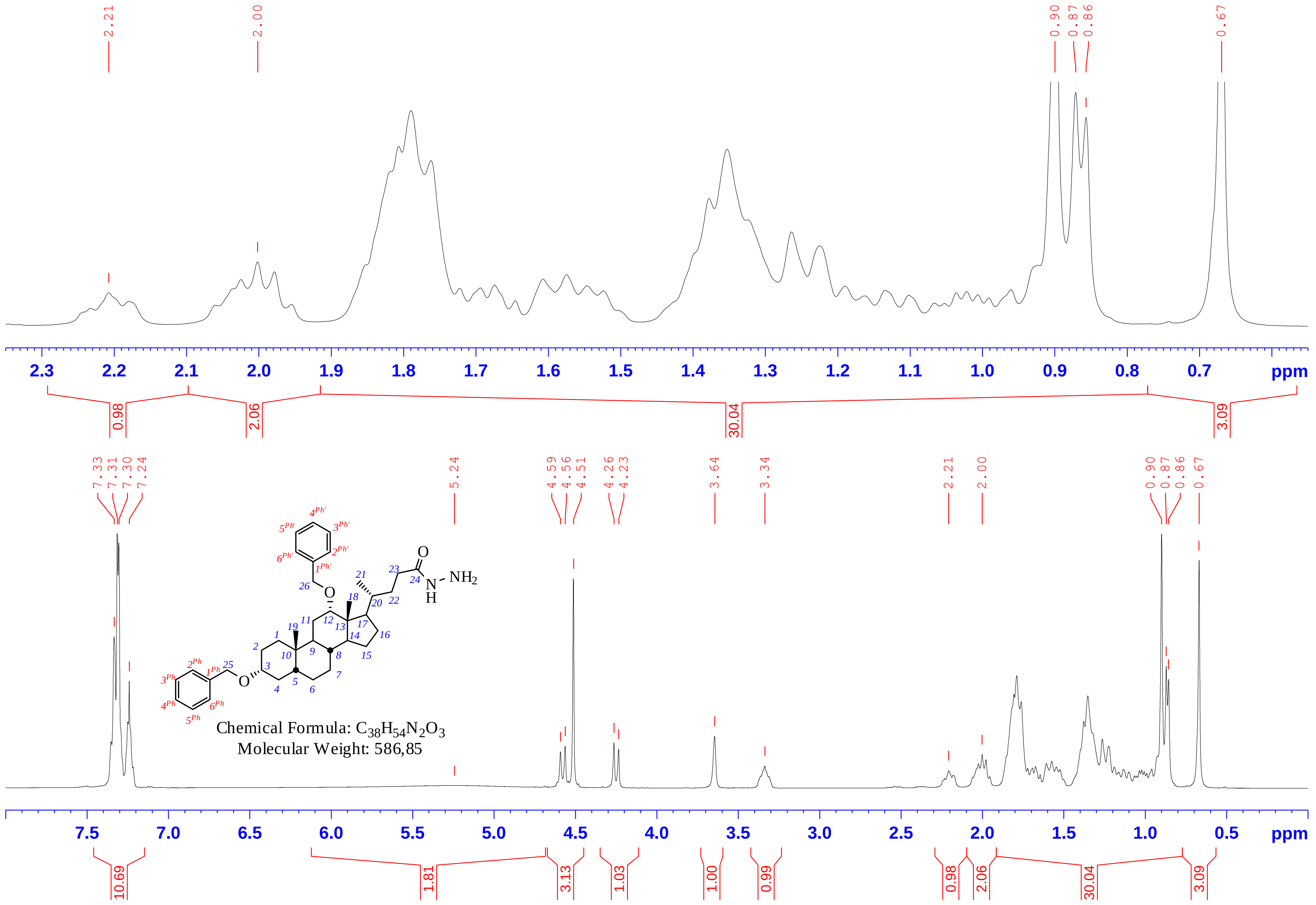

Spectrum of Compound **17**, ^{13}C NMR, JMOD, 100MHz, $CDCl_3$

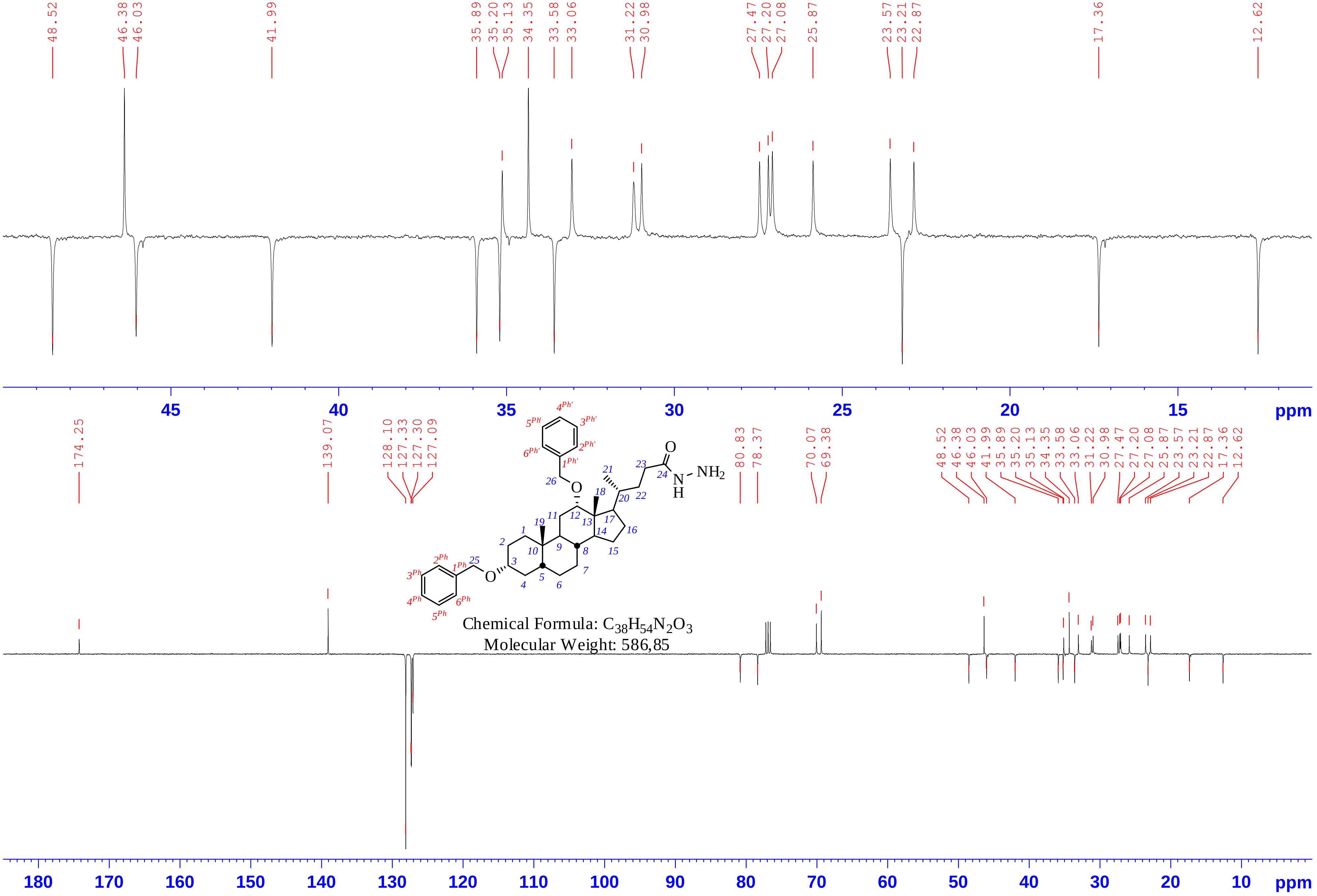

High resolution mass spectrum of compound **17**, Tsource=100°C, Tprobe=300°C

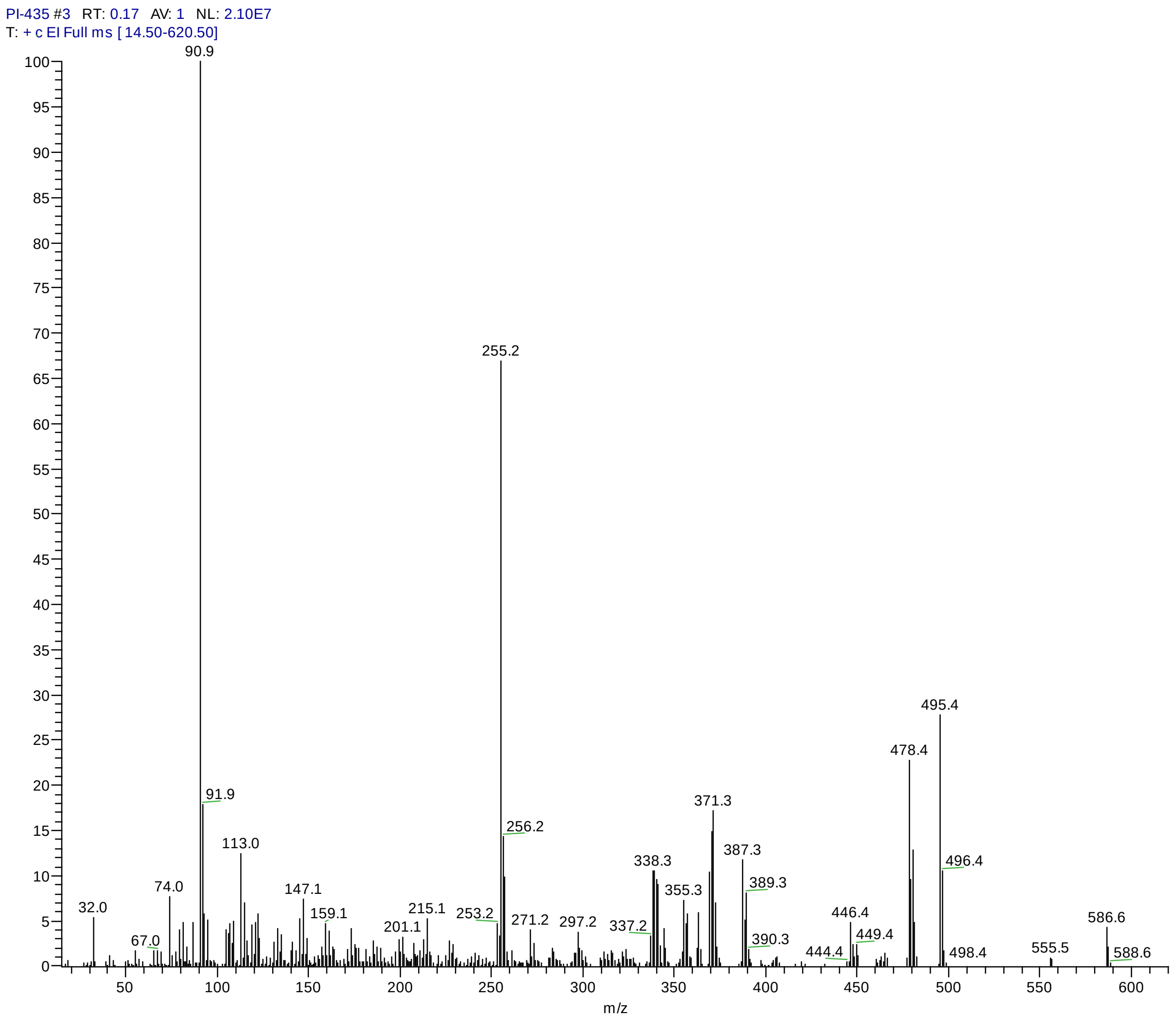

Calculated m/z= 586.4129 $(C_{38}H_{54}O_3N_2)^+$

Found m/z= 586.4131

Spectrum of Compound **18**, ^1H NMR, 300MHz, $CDCl_3$

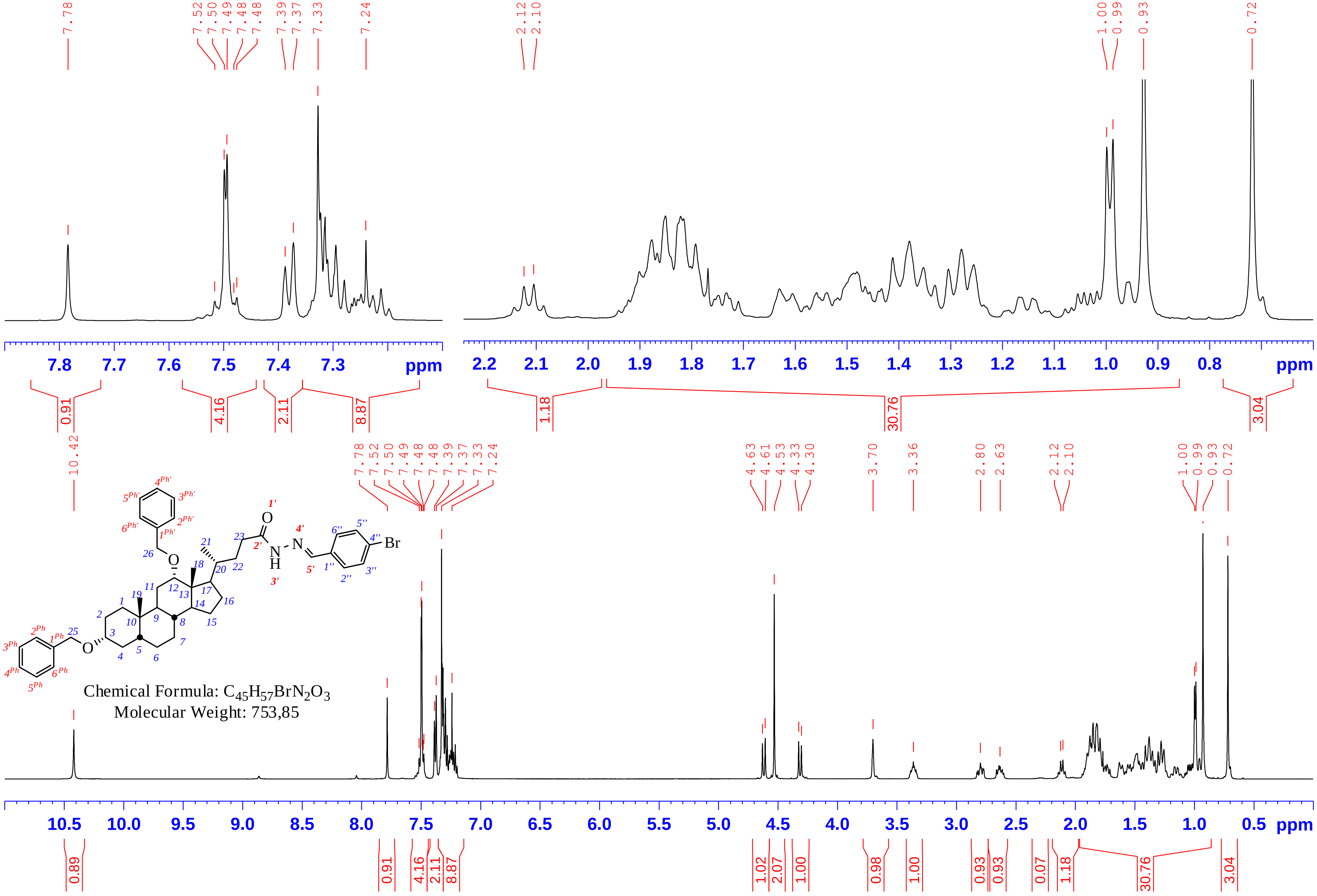

Spectrum of Compound **18**, ^{13}C NMR, JMOD, 75MHz, $CDCl_3$

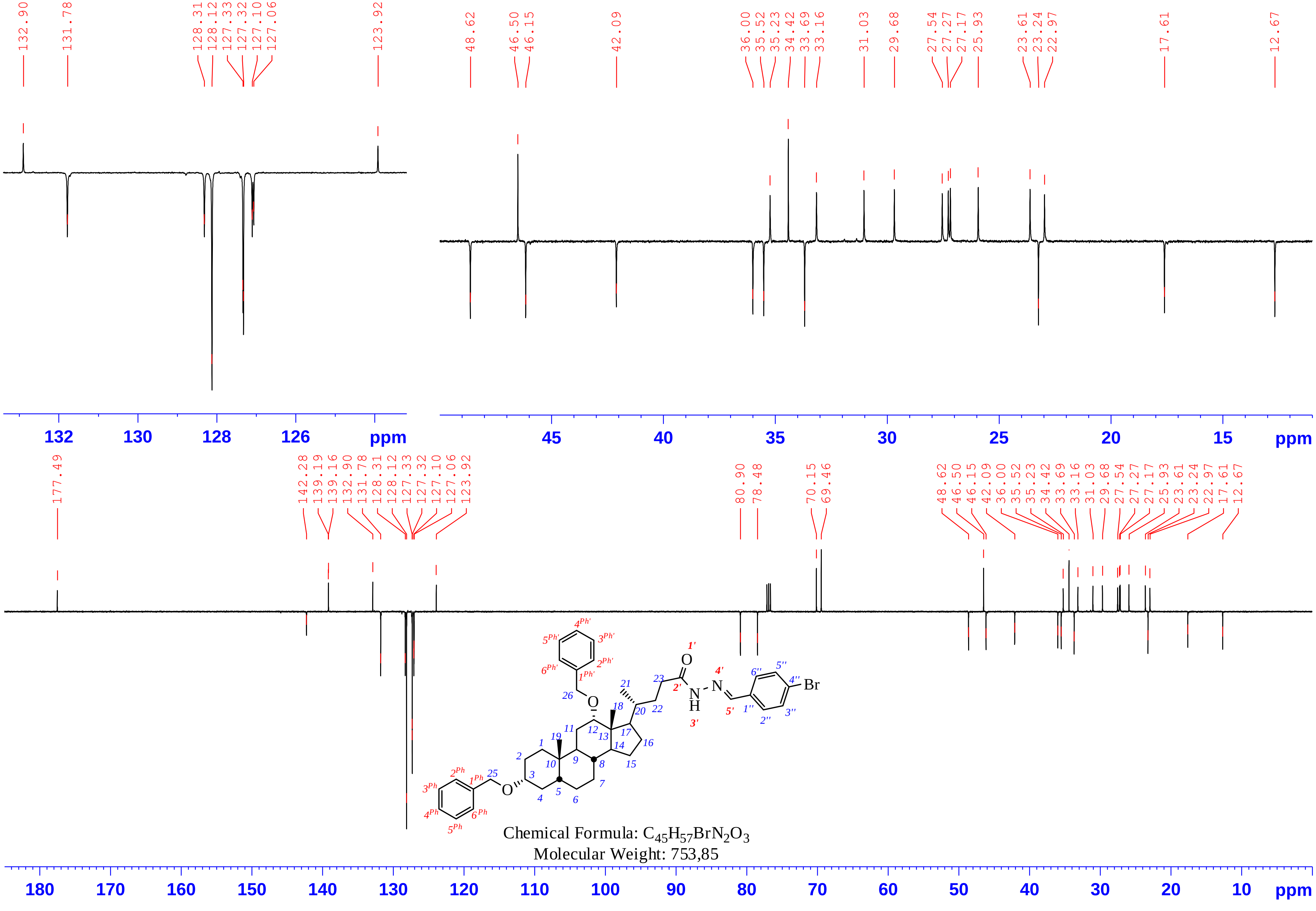

Chemical Formula: $C_{45}H_{57}BrN_2O_3$

Molecular Weight: 753,85

High resolution mass spectrum of compound **18**, Tsource=110°C, Tprobe=280°C

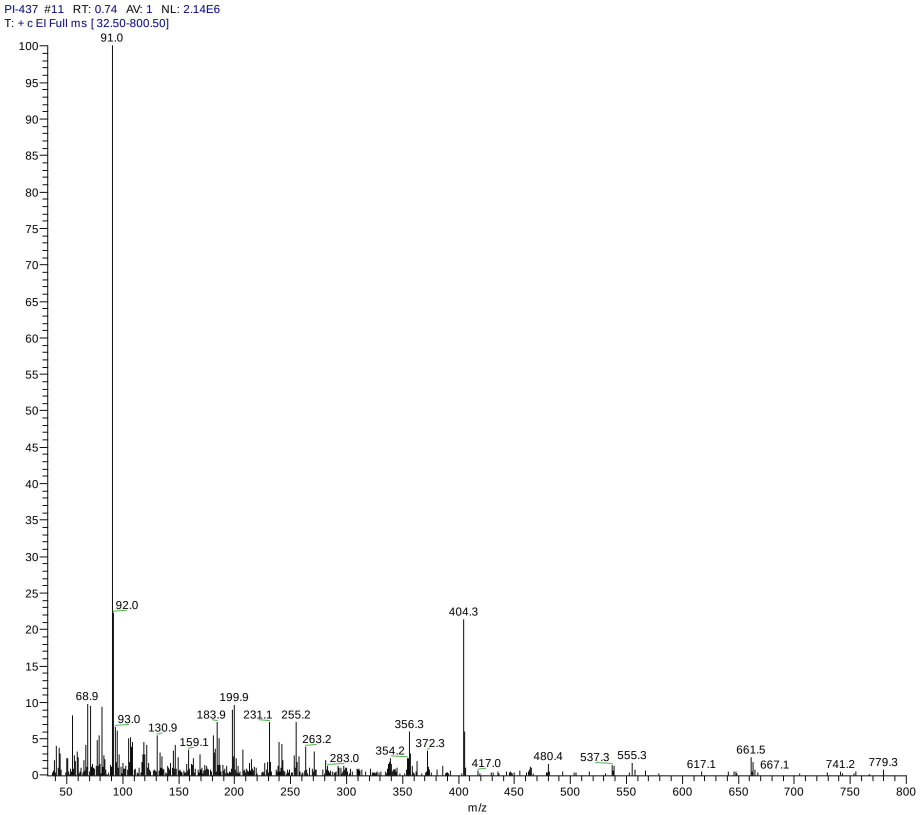

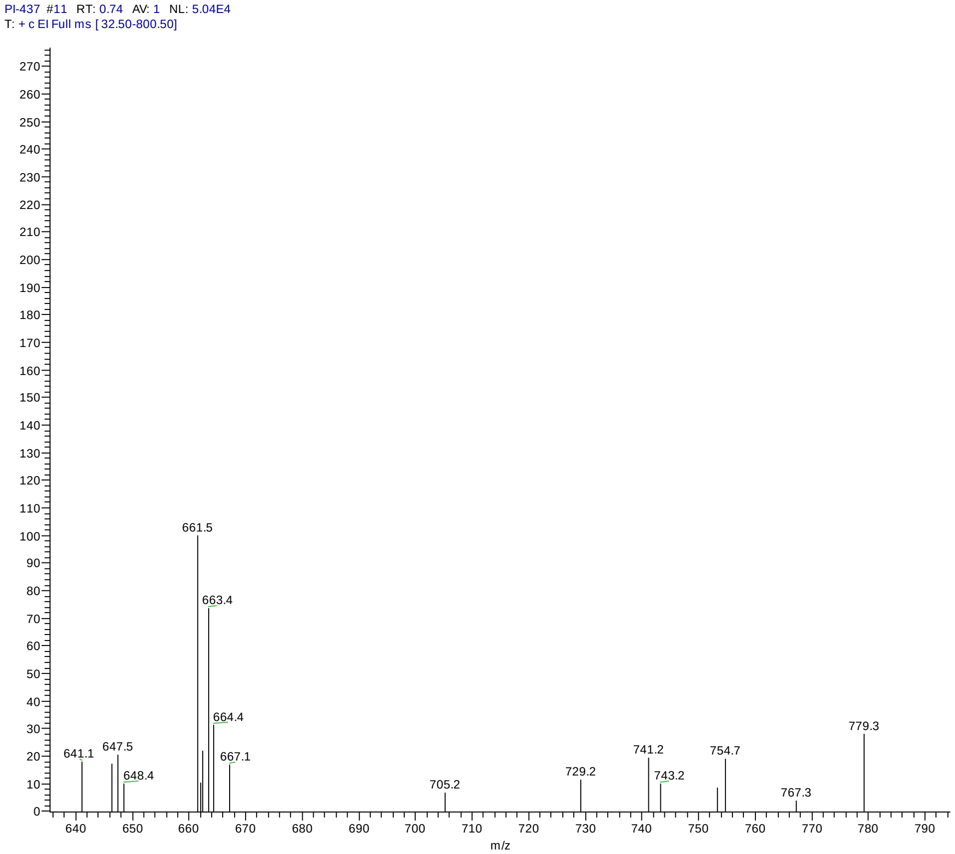

Calculated m/z=752.3547 $(C_{45}H_{57}O_3N_2{}^{79}Br_1)^+$ $[M]^+$

Found m/z=661.2988

Calculated m/z=661.2999 $(C_{38}H_{50}O_3N_2{}^{79}Br_1)^+$ $[M–PhCH_2]^+$

Spectrum of Compound **19**, ^{1}H NMR, 300MHz, $CDCl_3$

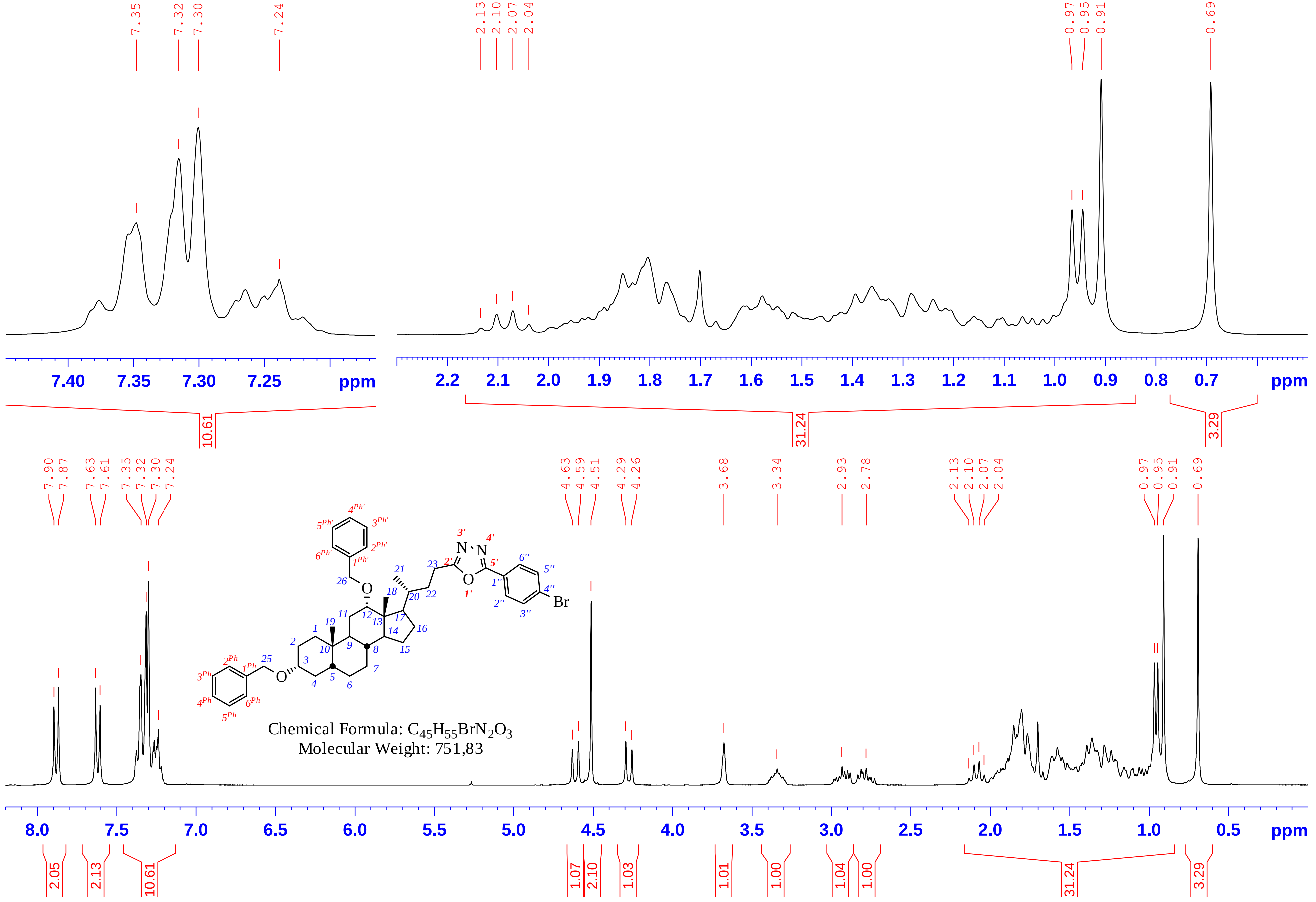

Spectrum of Compound **19**, ^{13}C NMR, JMOD, 100MHz, $CDCl_3$

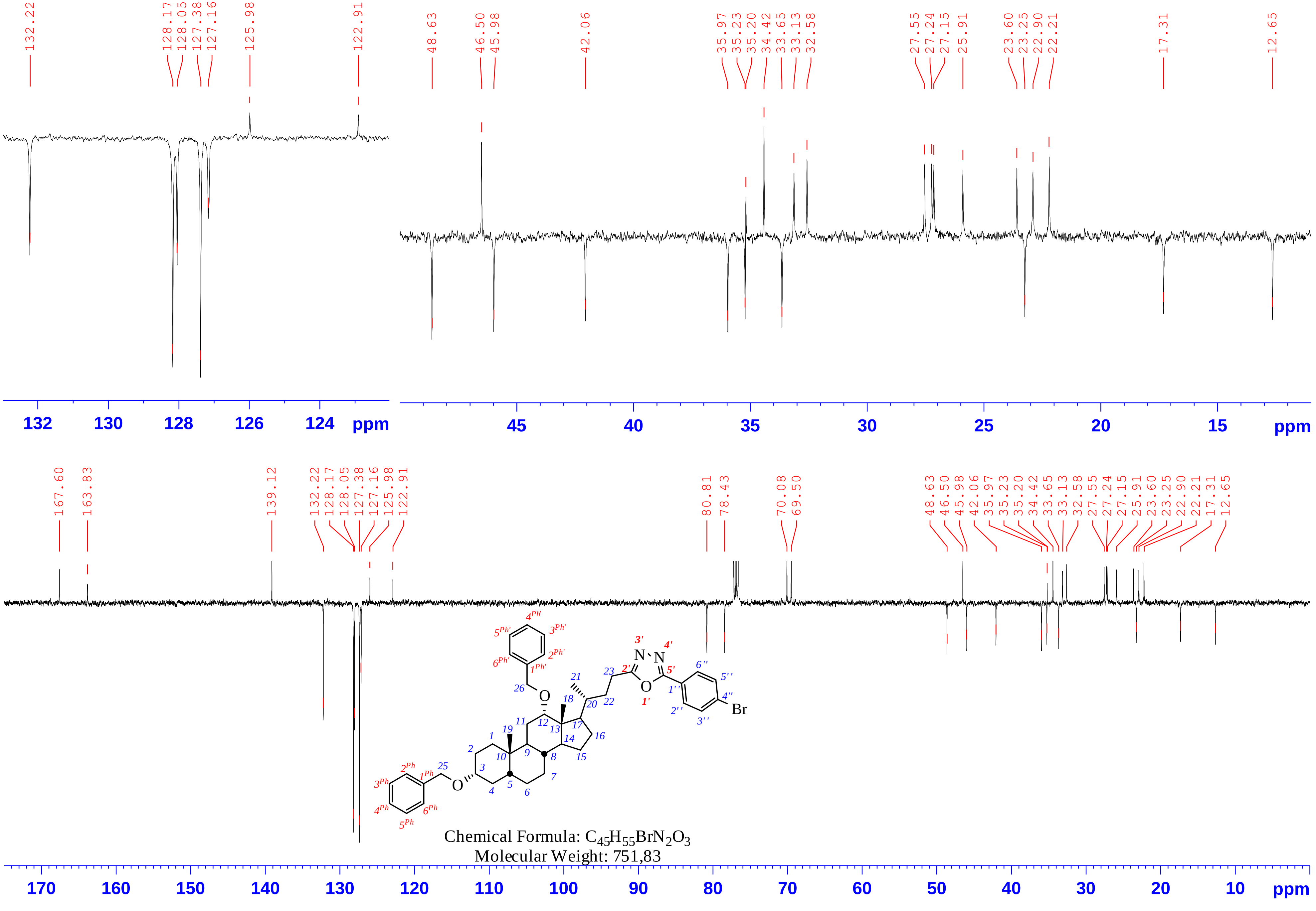

High resolution mass spectrum of compound **19**, Tsource=85°C, Tprobe=340°C

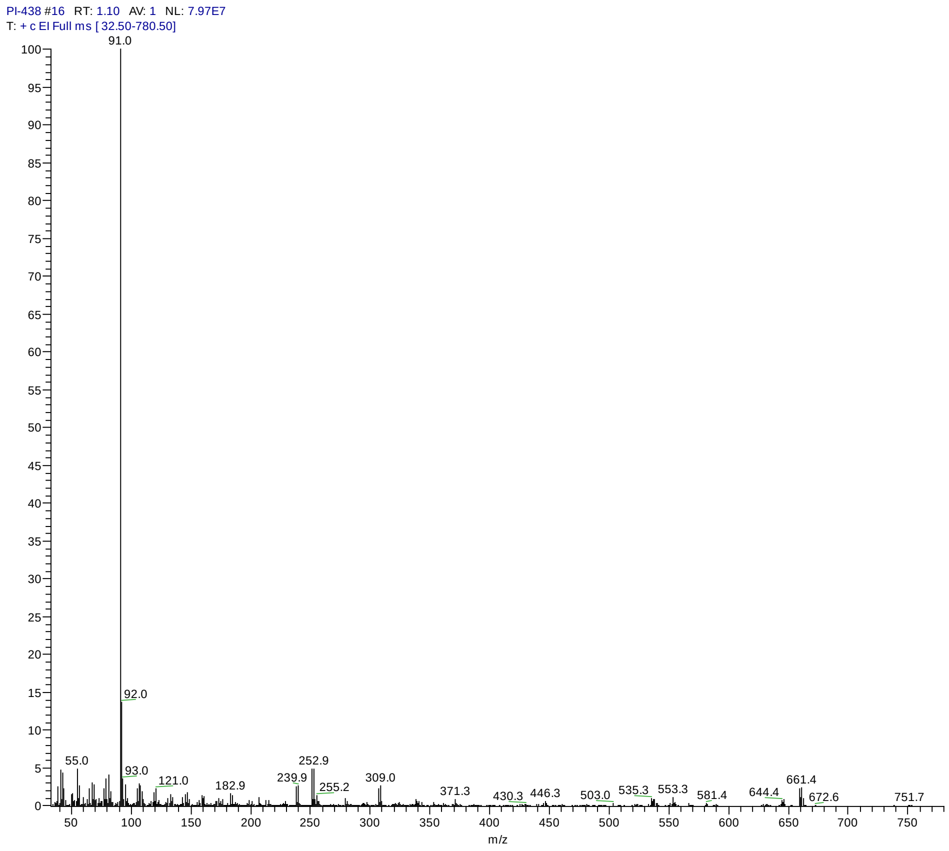

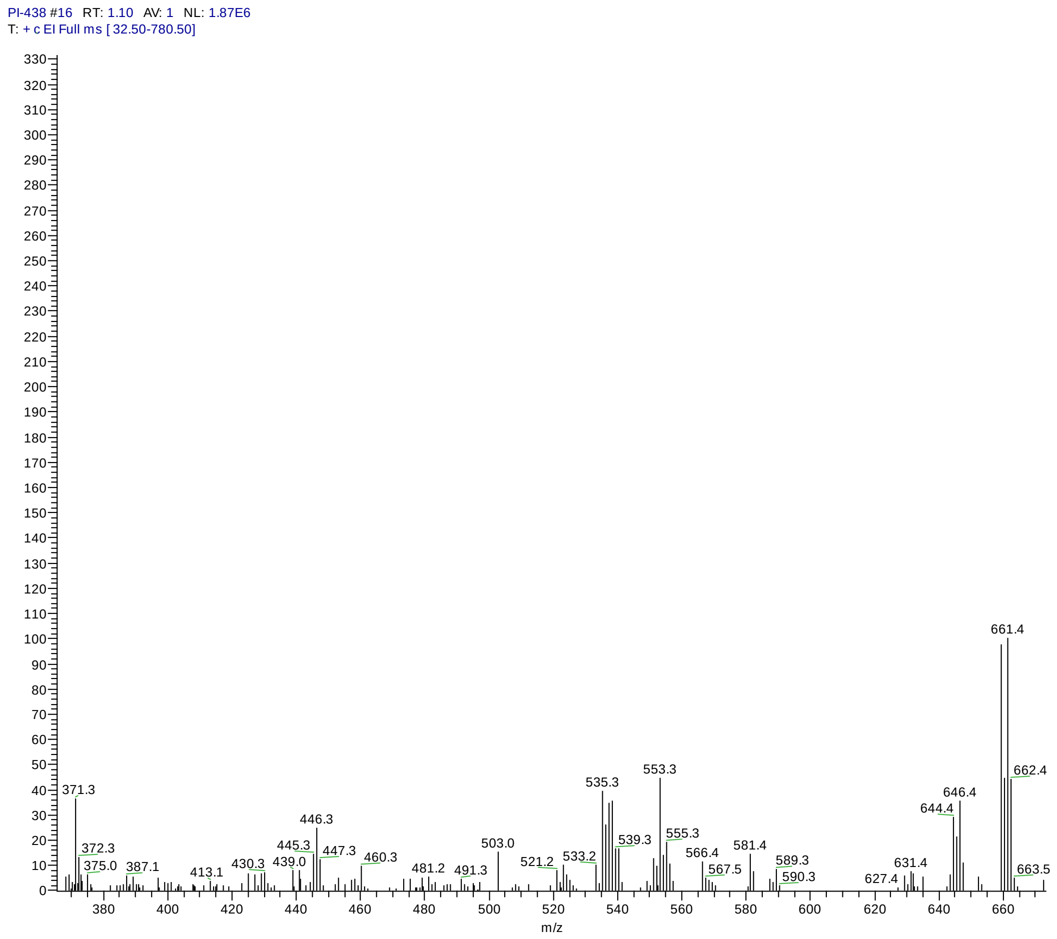

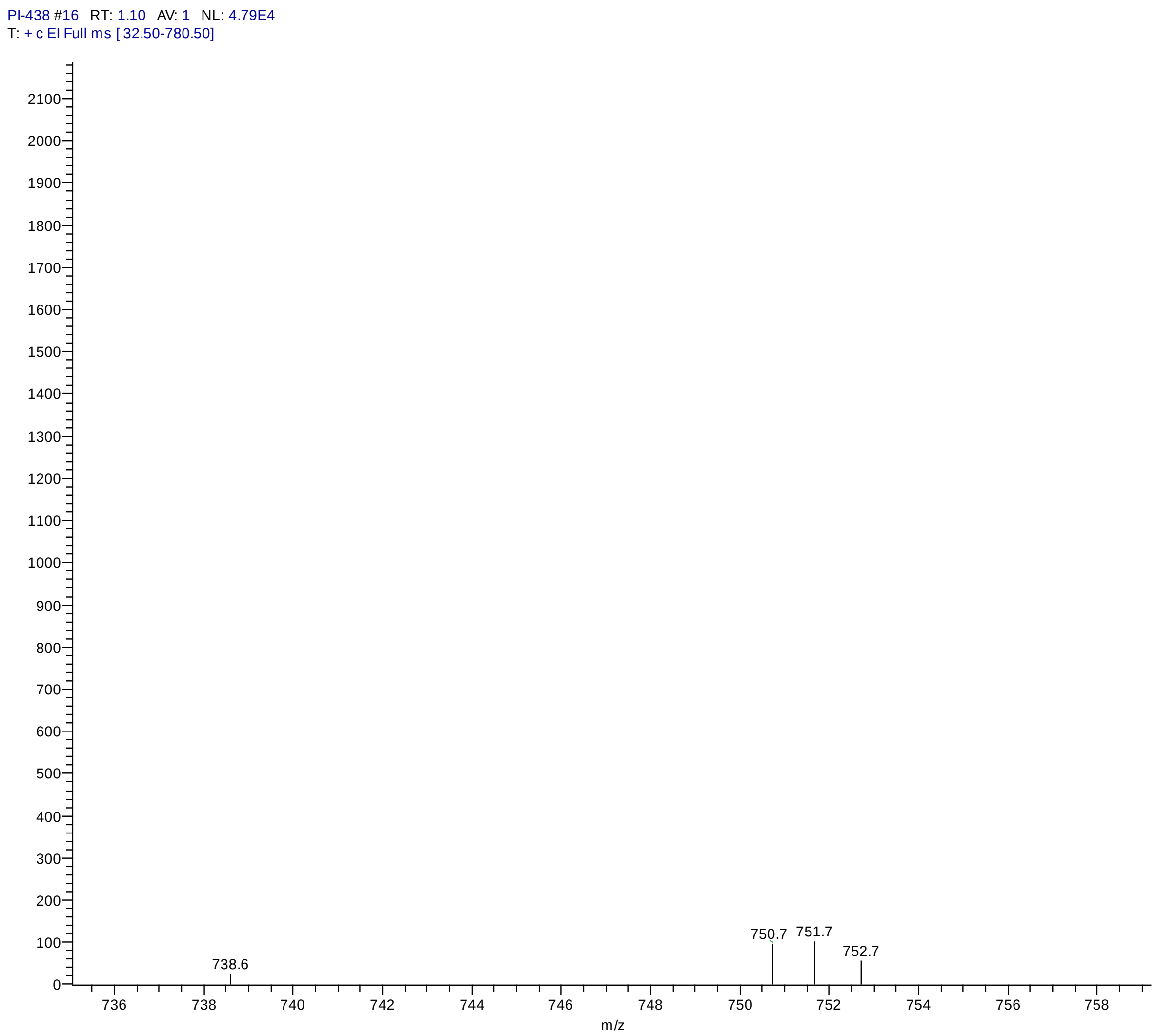

Calculated m/z=750.3391 ($(C_{45}H_{55}O_3N_2{}^{79}Br_1)^+$ $[M]^+$
Found m/z=659.2838
Found m/z=750.3354

Calculated m/z=659.2843 ($(C_{38}H_{48}O_3N_2{}^{79}Br_1)^+$ $[M–PhCH_2]^+$

Table S1. The influence of the deoxycholic acid derivative **8** at 10 μM on topotecan cytotoxicity

CI Data for Non-Constant Combo: (Tpc+**8** 10 μM)

**Dose Tpc**	**Dose 8**	**Effect**	**CI**
0.5	10.0	0.67	0.24464
1.0	10.0	0.78	0.28170
2.0	10.0	0.92	0.17568
4.0	10.0	0.93	0.30456

Table S2. The influence of the deoxycholic acid derivative **5** at 10 μM on topotecan cytotoxicity

CI Data for Non-Constant Combo: (Tpc+**5** 10 μM)

**Dose Tpc**	**Dose 5**	**Effect**	**CI**
0.3	10.0	0.61	0.44629
0.5	10.0	0.76	0.37480
1.0	10.0	0.86	0.39373
2.0	10.0	0.95	0.26285
4.0	10.0	0.96	0.41895

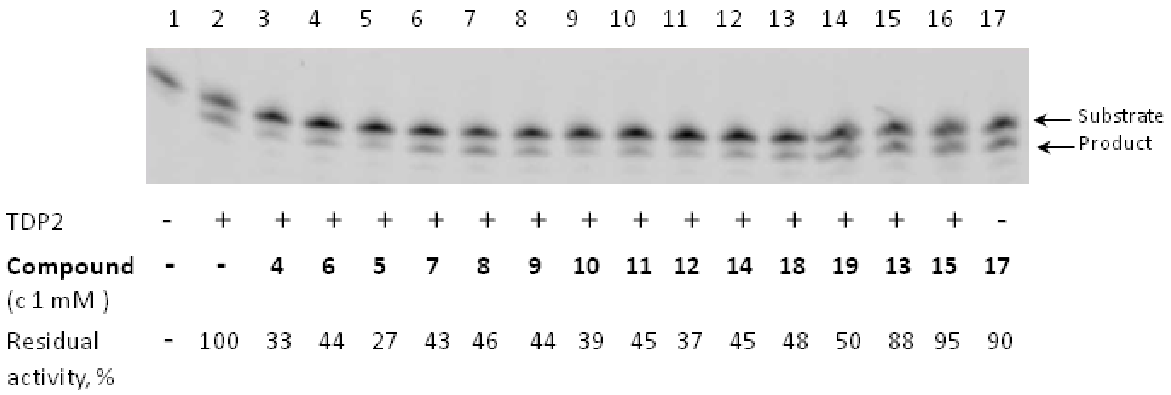

Figure S1. The compounds **4-19** inhibit TDP2 in 1 mM concentration

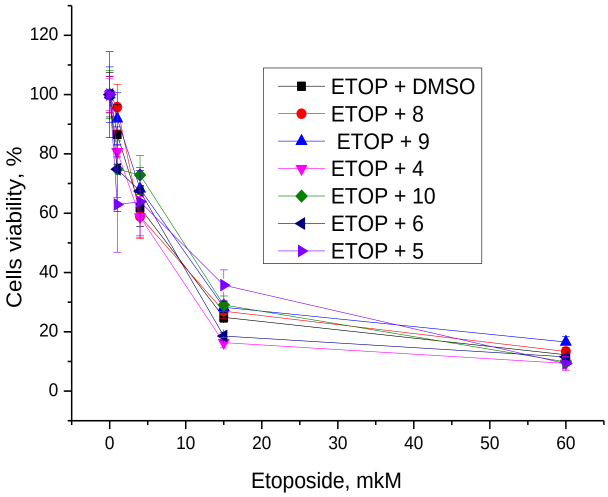

Figure S2. The influence of derivatives **4-6** and **8-10** (5 μM) on etoposide cytotoxicity against HeLa cells. Error bars show standard deviations.

Molecular modelling section

The co-crystallized ligand of TDP2 (6FQ) was removed and re-docked into the binding site to test the reliability of the docking scaffold and the scoring functions GoldScore (GS) [43], ChemScore (CS) [44-45], ChemPLP (Piecewise Linear Potential) [46] and ASP (Astex Statistical Potential) [47] in the GOLD (v2020.2.0)) docking algorithm were used. The GOLD docking algorithm is reported to be an excellent molecular modelling tool [51,52]. The predicted poses were overlain with the co-crystallized ligand and the root-mean-square deviations (RMSD) were calculated for the heavy atoms. Good values with < 2 Å were obtained for ChemPLP and GS, however ASP and CS had much poorer results (see Table S3). This can be explained by the relatively low resolution of the crystal structure (3.40 Å).

The binding scores, for the TDP1 catalytic pocket, are given in Table S4; all the ligands have reasonable values. No correlation is seen with their corresponding IC_{50} values for the scoring functions. All the ligands have relatively good IC_{50} values, and it can be argued that the scores are within the predictive capability of the scoring functions. The scoring results for TDP2 are shown in Table S3 and again reasonable values were predicted for all the ligands. For the scores there is no statistical difference between the averages of experimentally active vs inactive ligands. Interestingly, the ligands are predicted to have better binding than the co-crystallized ligand 6FQ. When the scores are compared between the TDP1 and TDP2 enzymes in all cases the former has slightly higher values indicating that the ligands bind preferably to TDP1.

[51] Wang, Z.; Sun, H.; Yao, X.; Li, D.; Xu, L.; Li, Y.; Tian, S.; Hou, T. Comprehensive Evaluation of Ten Docking Programs on a Diverse Set of Protein–ligand Complexes: The Prediction Accuracy of Sampling Power and Scoring Power. *Phys. Chem. Chem. Phys: PCCP* **2016**, *18*, 12964–12975, https://doi.org/10.1039/c6cp01555g.
[52] Bissantz, C.; Folkers, G.; Rognan, D. Protein-Based Virtual Screening of Chemical Databases. 1. Evaluation of Different Docking/Scoring Combinations. *J. Med. Chem.* **2000**, *43*, 4759–4767, https://doi.org/10.1021/jm001044

Table S3. The binding affinities as predicted by the scoring functions used to the Tdp2 binding site as well as the RMSD values for the co-crystallised ligand (6FQ).

**Ligands**	**ASP**	**ChemPLP**	**CS**	**GS**	***c* 200 μM**
**1**	37.8	68.6	30.5	58.9	+
**2**	35.7	59.7	28.1	52.0	+
**3**	37.4	54.6	26.5	46.0	+
**4**	40.4	80.4	41.4	59.6	+
**5**	39.3	71.2	34.6	45.7	+
**6**	39.8	70.6	36.4	58.9	+
**7**	37.8	76.9	35.2	56.4	+
**8**	35.6	64.1	32.7	51.8	+
**9**	37.2	65.2	31.2	53.0	+
**10**	44.8	88.5	43.0	58.6	+
**12**	41.0	84.0	37.8	63.8	+
**13**	40.9	80.7	33.6	65.1	-
**14**	45.2	82.3	38.5	63.7	+
**15**	38.9	75.4	34.2	57.5	-
**16**	33.4	69.2	33.5	54.1	-
**17**	38.3	71.3	33.4	54.5	+
**18**	38.0	78.0	33.5	55.9	+
**DCA**	29.5	46.5	24.1	37.2	
**6FQ**	31.0	46.3	22.5	45.7	
**RMSD (Å):**	8.257	1.598	5.244	1.778	

Table S4. The binding affinities as predicted by the scoring functions used to the catalytic Tdp1 binding pocket.

Ligands	ASP	ChemPLP	CS	GS	IC_{50} (μM)
**1**	41.8	48.3	34.8	57.7	0.44
**2**	33.2	62.8	29.4	47.6	18.3
**3**	34.3	59.6	28.7	43.6	0.78
**4**	47.8	94.5	43.3	63.6	0.75
**5**	42.1	83.0	39.5	54.6	0.24
**6**	45.4	82.9	42.1	62.5	0.59
**7**	38.0	74.3	38.7	56.4	0.97
**8**	39.8	68.7	34.1	50.4	0.57
**9**	38.8	65.5	36.4	58.4	0.71
**10**	48.2	94.9	43.0	64.5	0.37
**12**	44.6	91.2	40.8	63.7	0.32
**13**	39.1	83.4	35.7	68.0	0.53
**14**	40.2	82.6	42.1	61.3	0.33
**15**	39.3	81.2	38.3	64.3	0.42
**16**	39.7	79.5	33.7	47.0	0.23
**17**	44.6	81.7	38.4	60.6	0.51
**18**	43.0	81.6	40.0	62.7	1.01
**DCA**	27.9	49.8	24.0	48.9	5.6

Table S5. The molecular descriptors and their corresponding Known Drug Indexes 2a and 2b ($KDI_{2a/2b}$). The R^2 numbers derived do not contain derivatives **13** and **DCA** since they are outlier.

	RB	MW	HD	HA	Log P	PSA	KDI_{2A}	KDI_{2B}	Tdp1 IC_{50} (μM)	Tdp2 (inhibitor concentration 1 mM)
**1**	10	572.8	1	5.4	8.7	63.5	3.35	0.00	0.44	+
**2**	8	482.7	2	5.4	6.5	76.9	4.46	0.09	18.3	+
**3**	7	480.7	1	5.7	6.5	80.0	4.52	0.10	0.78	+
**4**	13	715.0	2	5.9	10.2	67.8	3.04	0.00	0.75	+
**5**	11	624.9	3	5.9	8.0	81.1	3.13	0.00	0.24	+
**6**	10	622.9	2	6.2	8.0	84.2	3.42	0.00	0.59	+
**7**	11	726.8	1	5.9	10.8	46.0	2.89	0.00	0.97	+
**8**	9	636.7	2	5.9	8.6	62.1	3.48	0.00	0.57	+
**9**	8	634.7	1	6.2	8.7	65.1	3.53	0.00	0.71	+
**10**	17	818.2	2	6.65	12.5	65.4	2.92	0.00	0.37	+
**12**	14	783.9	2	7.4	10.7	96.1	2.82	0.00	0.32	+
**13**	12	765.9	0	6.4	11.3	49.4	2.48	0.00	0.53	-
**14**	14	769.9	2	7.4	10.4	85.6	2.86	0.00	0.33	+
**15**	10	751.8	0	6.4	11.0	47.6	2.65	0.00	0.42	-
**16**	11	586.9	3	6.4	7.4	83.3	3.24	0.00	0.23	-
**17**	13	753.9	1	5.9	11.4	64.5	2.89	0.00	0.51	+
**18**	10	751.8	0	5.9	11.3	47.4	2.68	0.00	1.01	+
**DCA**	6	392.6	3	5.4	3.8	91.2	5.57	0.63	5.6	
**Tdp1 R^2:**	0.177(-)	0.004	0.306(-)	0.232(-)	0.008	0.303(-)	0.023	0.061		

Table S6. Definition of lead-like, drug-like and Known Drug Space (KDS) in terms of molecular descriptors. The values given are the maxima for each descriptor for the volumes of chemical space used.

	**Lead-like Space**	**Drug-like Space**	**Known Drug Space**
Molecular weight (g mol^{-1})	300	500	800
Lipophilicity (Log P)	3	5	6.5
Hydrogen bond donors (HD)	3	5	7
Hydrogen bond acceptors (HA)	3	10	15
Polar surface area ($Å^2$) (PSA)	60	140	180
Rotatable bonds (RB)	3	10	17

Table S7. Structures of DCA derivatives. Effect of benzyloxy *vs* acetoxy groups in the steroid scaffold on Tdp1.

	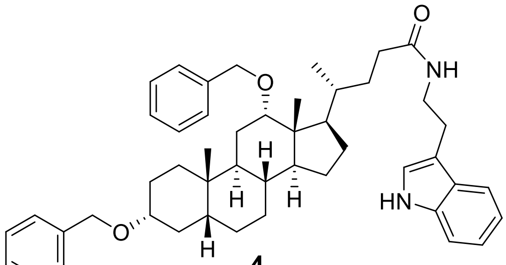 **4**<br><br>**IC_{50}, µM** (Tdp1) 0.75±0.17	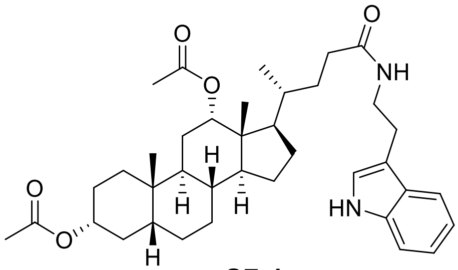 **S7-1**<br><br>**IC_{50}, µM** (Tdp1) 0.65±0.16
	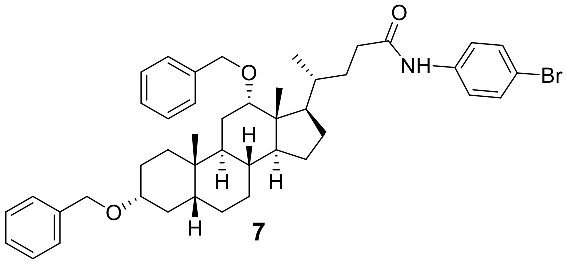 **7**<br><br>**IC_{50}, µM** (Tdp1) 0.97±0.46	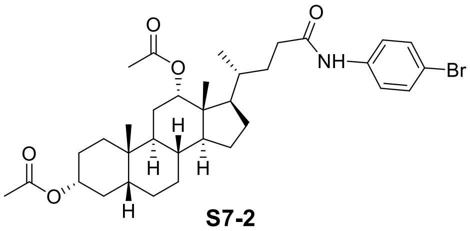 **S7-2**<br><br>**IC_{50}, µM** (Tdp1) 0.42±0.01
	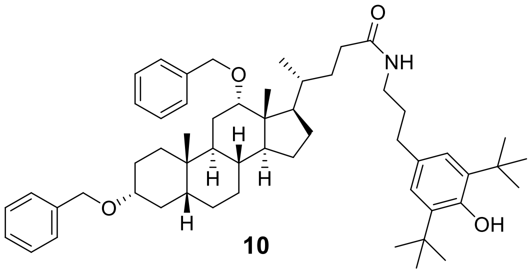 **10**<br><br>**IC_{50}, µM** (Tdp1) 0.37±0	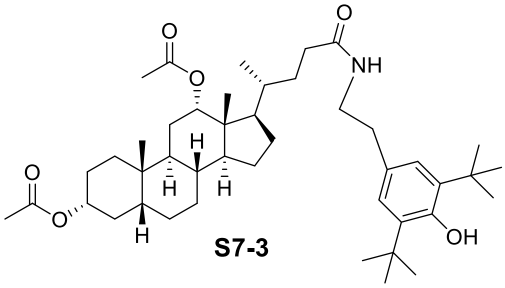 **S7-3**<br><br>**IC_{50}, µM** (Tdp1) 0.29±0.12

Table S8. Structures of DCA derivatives. Effect of benzyloxy *vs* methoxy groups in the steroid scaffold on Tdp1.

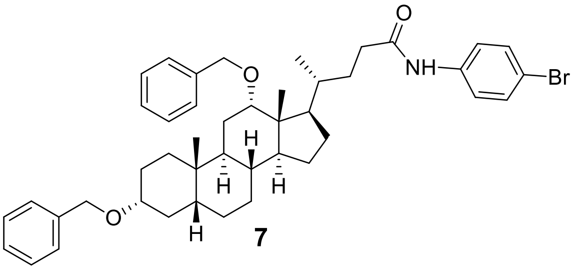 **7**<br><br>**IC_{50}, µM** (Tdp1) 0.97±0.46	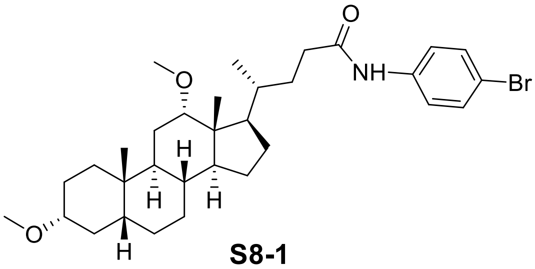 **S8-1**<br><br>**IC_{50}, µM** (Tdp1) 0.27 ±0.01
